# Genome-Wide Identification of the Cyclic Nucleotide-Gated Ion Channel Gene Family and Expression Profiles Under Low-Temperature Stress in *Luffa cylindrica* L.

**DOI:** 10.3390/ijms252011330

**Published:** 2024-10-21

**Authors:** Jianting Liu, Yuqian Wang, Lijuan Peng, Mindong Chen, Xinru Ye, Yongping Li, Zuliang Li, Qingfang Wen, Haisheng Zhu

**Affiliations:** 1 Fujian Key Laboratory of Vegetable Genetics and Breeding, Crops Research Institute, Fujian Academy of Agricultural Sciences, Fuzhou 350013, China; ljt338625@163.com (J.L.); 15059483846@163.com (M.C.); zwsyexr@163.com (X.Y.); lyp_lily@163.com (Y.L.); lzuliang@163.com (Z.L.); 2 Vegetable Research Center, Fujian Academy of Agricultural Sciences, Fuzhou 350013, China; 3 College of Horticulture, Fujian Agriculture and Forestry University, Fuzhou 350003, China; 15136739206@163.com (Y.W.); plj_678@163.com (L.P.)

**Keywords:** *Luffa cylindrica* L., *CNGC*, genome-wide identification, low temperature, expression analysis

## Abstract

Cyclic nucleotide-gated ion channels (CNGCs) are cell membrane channel proteins for calcium ions. They have been reported to play important roles in survival and in the responses to environmental factors in various plants. However, little is known about the CNGC family and its functions in luffa (*Luffa cylindrica* L.). In this study, a bioinformatics-based method was used to identify members of the *CNGC* gene family in *L. cylindrica*. In total, 20 *LcCNGCs* were detected, and they were grouped into five subfamilies (I, II, Ⅲ, IV-a, and IV-b) in a phylogenetic analysis with CNGCs from *Arabidopsis thaliana* (20 *AtCNGCs*) and *Momordica charantia* (17 *McCNGCs*). The 20 *LcCNGC* genes were unevenly distributed on 11 of the 13 chromosomes in luffa, with none on Chromosomes 1 and 5. The members of each subfamily encoded proteins with highly conserved functional domains. An evolutionary analysis of CNGCs in luffa revealed three gene losses and a motif deletion. An examination of gene replication events during evolution indicated that two tandemly duplicated gene pairs were the primary driving force behind the evolution of the *LcCNGC* gene family. PlantCARE analyses of the *LcCNGC* promoter regions revealed various *cis*-regulatory elements, including those responsive to plant hormones (abscisic acid, methyl jasmonate, and salicylic acid) and abiotic stresses (light, drought, and low temperature). The presence of these *cis*-acting elements suggested that the encoded CNGC proteins may be involved in stress responses, as well as growth and development. Transcriptome sequencing (RNA-seq) analyses revealed tissue-specific expression patterns of *LcCNGCs* in various plant parts (roots, stems, leaves, flowers, and fruit) and the upregulation of some *LcCNGCs* under low-temperature stress. To confirm the accuracy of the RNA-seq data, 10 cold-responsive *LcCNGC* genes were selected for verification by quantitative real-time polymerase chain reaction (RT-qPCR) analysis. Under cold conditions, *LcCNGC4* was highly upregulated (>50-fold increase in its transcript levels), and *LcCNGC3*, *LcCNGC6*, and *LcCNGC13* were upregulated approximately 10-fold. Our findings provide new information about the evolution of the *CNGC* family in *L. cylindrica* and provide insights into the functions of the encoded CNGC proteins.

## 1. Introduction

The cyclic nucleotide-gated channel (CNGC) gene family encodes non-selective cation channels, which are mainly distributed on the plasma membrane. These proteins are distributed in animals and plants and are important components of the eukaryotic signaling cascade. They are known to play important roles in physiological processes such as plant signal transduction [1,2,3], growth and development [4], and responses to environmental stresses [5,6]. A *CNGC* gene in the barley (*Triticum aestivum*) aleurone layer was the first one to be identified [7]. Subsequently, whole-genome sequencing and functional genomics studies on various plants have revealed that the *CNGC* gene family is one of the most important gene families in plants [8,9]. Many *CNGC* genes have been identified from various plants such as *Triticum aestivum* [10], *Arabidopsis thaliana* [11], *Oryza sativa* [12], *Brassica oleracea* [13], *Solanum melongena* [14], *Brassica rapa* [15], *Malus domestica* [16], *Nicotiana tabacum* [17], *Ziziphus jujuba* [9], *Hordeum vulgare* [18], *Saccharum spontaneum* [19], and *Mangifera indica* [20]. Plant CNGCs can be divided into five groups (i.e., I, II, III, IV-a, and IV-b) according to their phylogenetic relationships. The CNGCs are mainly characterized by their core transmembrane domains (i.e., CNBD, CaMBD, and IQD motifs) [9,18,20,21]. These motifs have special properties that are necessary for protein function and are widely used to identify plant CNGCs. Because the CNBD motif is highly conserved, it can be used to identify *CNGC* genes in various plant species [22,23].

There have been many studies on the regulation of *DREB1* expression and the expression of genes downstream of *DREB1*. However, few studies have focused on the roles of genes upstream of *DREB1* in the perception of decreased temperature in plants [24]. In *Arabidopsis*, a mutant with a defective *AtCNGC20* gene showed a low-temperature-sensitive phenotype, whereas *CNGC20*-overexpressing plants showed a cold-tolerant phenotype [25]. According to studies on rice (*O. sativa*), OsCNGC9 positively regulates cold tolerance by mediating an increase in calcium levels in the cytoplasm. OsCNGC9-deficient mutants showed deficient calcium inflow under low-temperature conditions, making them more sensitive to a prolonged low-temperature treatment, whereas those overexpressing OsCNGC9 showed increased cold tolerance [26]. Moreover, two closely related CNGC proteins in rice, OsCNGC14 and OsCNGC16, were found to enhance plant growth during low-temperature stress and increase cold tolerance [27].

Luffa (*Luffa cylindrica* L.) is an important vegetable worldwide. It grows best in a warm and light growing environment, and it is a short-day plant [28]. Studies have shown that continuous low temperature in cucurbit crops before flowering can cause damage such as flower topping, poor fertilization, and reduced fruit set, and low-temperature encountered in the seedling stage tends to slow down the root growth and shorten the plants, which seriously affects the distribution of the crops, their yields, and their quality [26,29,30,31,32]. During cultivation, luffa plants are sensitive to damage by extreme weather conditions, such as low temperature and sparse sunlight. Such conditions cause flowers and fruits to fall and can result in seedling death and the incidence of various diseases, leading to reduced luffa production [28,33]. Consequently, in the process of luffa production, low-temperature disasters can lead to a luffa yield reduction of 20% to 50%, and in severe cases, it can be up to 70% and even cause the extinction of the harvest. To address these problems, the best solution is to cultivate cold-resistant luffa varieties. At present, only a few cold-tolerant luffa varieties have been bred, possibly because of a lack of efficient breeding techniques. Additionally, there are few reports on the gene resources and molecular mechanisms underlying cold tolerance in luffa, and this has seriously hindered the breeding and cultivation of stress-tolerant varieties [28].

With the release of reference genome sequences for many plants, the body of research on the *CNGC* gene family has increased. Members of this gene family have been detected and characterized from diverse plants including *Arabidopsis* [34], *Ziziphus jujuba* [9], *O. sativa* [12], *Zea mays* [8], *Gossypium* [35], *Lycopersicon esculentum* [36], *Saccharum spontaneum* [19], *Solanum melongena* [14], *Brassica rapa* [23], and *Malus domestica* [16]. The recently released genome sequences of Cucurbitaceae crops, such as *Benincasa hispida* [37], *Citrullus lanatus* [38], *Cucurbita máxima* [39], *Cucumis melo* [40], *Cucurbita pepo* [41], *L. acutangula* Roxb. [42], and *L. cylindrica* Roem. [43], are useful resources for genome-wide analyses of Cucurbitaceae species. However, no previous studies have identified and characterized the *CNGC* family in luffa.

## 2. Results

### 2.1. Identification and Classification of CNGC Genes in L. cylindrica

Twenty candidate *CNGC* sequences were detected in the whole luffa genome using BLAST searches (Supplementary File S1), and domain composition analyses confirmed that all of the candidate genes encoded CNGC proteins (Supplementary File S2). On the basis of published classification criteria for CNGCs, the amino acid sequences of 18 LcCNGCs (all except for LcCNGC19 and LcCNGC20) contained both an ITP domain and a CNBD [9]. Twenty potential CNGC proteins were detected in the luffa protein database using the CNGC HMM search. These protein-coding genes were named *LcCNGC1*–*LcCNGC20* based on their distribution on luffa chromosomes (Table 1). The length of the open reading frame of the *LcCNGC* genes ranged from 1230 to 2310 bp. Each LcCNGC has an average of four exons (range: 1–12). The LcCNGC proteins range in length from 410 to 770 amino acids with predicted molecular weights of 42.27–88.68 kDa. The predicted isoelectric points ranged from 8.53 to 9.58. Except for LcCNGC3, the other 19 LcCNGC proteins were predicted to localize to the cell membrane.

### 2.2. Chromosome Mapping and Replication of L. cylindrica CNGC Genes

**Figure 1:**
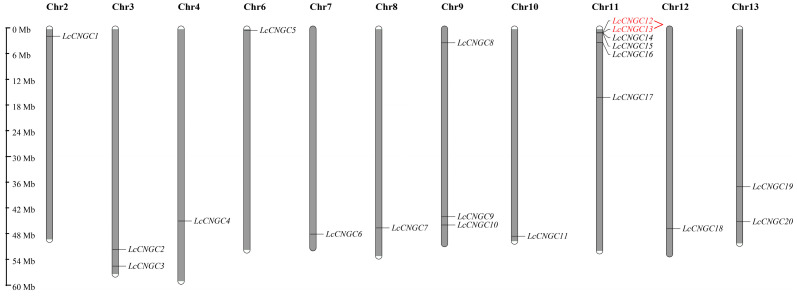
Chromosomal location of *L. cylindrica CNGC* genes. The scale is shown on the left. Tandemly repeated genes are marked in red.

The physical locations of the 20 *LcCNGC* genes on 11 luffa chromosomes (all except Chromosomes 1 and 5) were determined. Previous studies have shown that gene clusters are considered if there were three or more genes from the same family in a 200 kb region [44]. In this study, we also found a luffa *LcCNGC* gene cluster consisting of four genes on Chromosome 11, (i.e., *LcCNGC12*, *LcCNGC13*, *LcCNGC14*, and *LcCNGC15*) (Table 1). In addition, two luffa *LcCNGC* gene duplication events were identified in this study. These duplication events involved two groups containing four genes (i.e., *LcCNGC1*/*LcCNGC12* and *LcCNGC11*/*LcCNGC15*), which accounted for 20% of all *LcCNGCs*, and both groups of duplication events were segmental duplications (Figure 1) [45]. Since 80% of *LcCNGC* genes are not associated with replication events, we hypothesized that the formation of the *LcCNGC* gene family may not be dependent on gene replication. The *Ka*/*Ks* ratio is often used as a measure of selection pressure at the gene sequence level and consequently reflects selection during the evolution of genes [46]. A *Ka*/*Ks* value greater than 1 indicates positive selection, 1 indicates neutral selection, and 0 to 1 indicates purifying selection. The *Ka*/*Ks* value of the tandemly duplicated gene pair *LcCNGC12*/*LcCNGC13* in luffa was 0.435, suggesting that this pair of *LcCNGC* genes was under significant purifying selection pressure and conservation of gene function during the evolutionary process (Supplementary File S3, Table S1).

### 2.3. Analysis of L. cylindrica CNGC Promoter Sequences and Gene Structures

**Figure 2:**
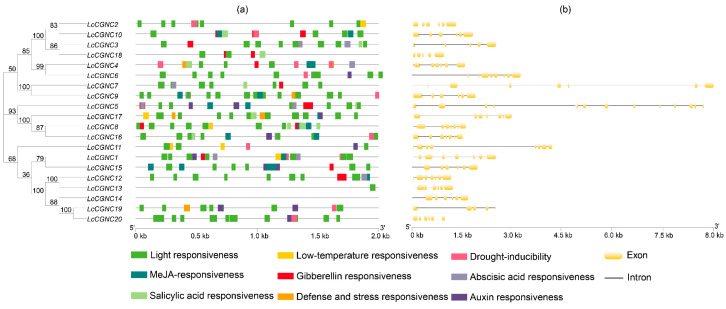
Analysis of *L. cylindrica CNGC* gene promoter regions and gene structures. (**a**) *CNGC* gene promoters in *L. cylindrica*. (**b**) *CNGC* gene structures in *L. cylindrica*.

In order to clarify the *cis*-acting element situation in the promoters of these *LcCNGC* genes, the study examined the sequences 2000 bp upstream of the initiation codon (ATG) of each luffa *LcCNGC* gene (*LcCNGC1*–*LcCNGC20*) using the PlantsCARE online software (http://bioinformatics.psb.ugent.be/webtools/plantcare/html/ accessed on 25 June 2024) (Supplementary File S4 and Figure 2a), in which a large number of hormone- and abiotic stress-related corresponding *cis*-acting elements were identified. Analysis revealed that most *LcCNGC* gene promoters contained multiple *cis*-acting elements, suggesting that the *LcCNGC* gene may be participating in the regulation of various physiological and metabolic pathways in luffa. In total, 2790 *cis*-acting elements were detected in the *LcCNGC* gene promoters. There were 732 common *cis*-acting elements in promoter and enhancer regions (CAAT-box), 429 unknown *cis*-acting elements, 160 light response elements, 103 hormone response elements, and 81 core promoter elements located around −30 relative to the transcription start site (TATA-box), accounting for 53.94% of all *cis*-acting elements. Further analyses revealed 288 hormone response or stress response elements (Figure 2a). Of those, the most abundant *cis*-acting elements were methyl jasmonate (MeJA) response and abscisic acid (ABA) response elements (38 and 24, respectively). This analysis indicated that 50% and 60% of all *cis*-acting elements in the *LcCNGC* genes were associated with MeJA and ABA, respectively. We also detected 17 gibberellin response elements. Moreover, there were 13 drought response elements, 12 salicylic acid response elements, and 12 auxin response elements, accounting for 4.5%, 4.1%, and 4.1% of all *cis*-acting elements, respectively. Furthermore, six defense and stress response elements and six low-temperature response elements were detected in the *CNGC* gene promoters. The functional diversity of the *LcCNGC* genes is likely due to the diversity of *cis*-acting elements in their promoter regions (Supplementary File S5). In addition, 33 MYB binding sites and 15 W-box elements were found in the *CNGC* gene promoters. These findings suggest that the *LcCNGC* genes in luffa are multifunctional, probably because they contain corresponding *cis*-acting elements that can be transcriptionally regulated by MYB or WRKY transcription factors, which are responsive to a variety of stresses including drought and low temperature.

In order to investigate the evolutionary conservation of *CNGC* family genes in luffa, we analyzed the conserved structure of the *LcCNGC* gene using the genome (Genbank accession number: PRJNA596077) annotation files. The results showed that the *LcCNGC* gene family members comprise 7–14 introns and 6–13 exons (Figure 2b). The most common genetic structure was seven exons and eight introns. Five *LcCNGC* genes (*LcCNGC2*, *LcCNGC3*, *LcCNGC9*, *LcCNGC10,* and *LcCNGC20*) contained six exons, three (*LcCNGC7*, *LcCNGC8*, and *LcCNGC11*) consisted of eight exons, and *LcCNGC5*, *LcCNGC6*, *LcCNGC18*, and *LcCNGC*19 contained 13, 9, 10, and 5 exons, respectively.

### 2.4. Phylogenetic Analysis of L. cylindrica CNGC Genes and Their Encoded CNGC Domains

**Figure 3:**
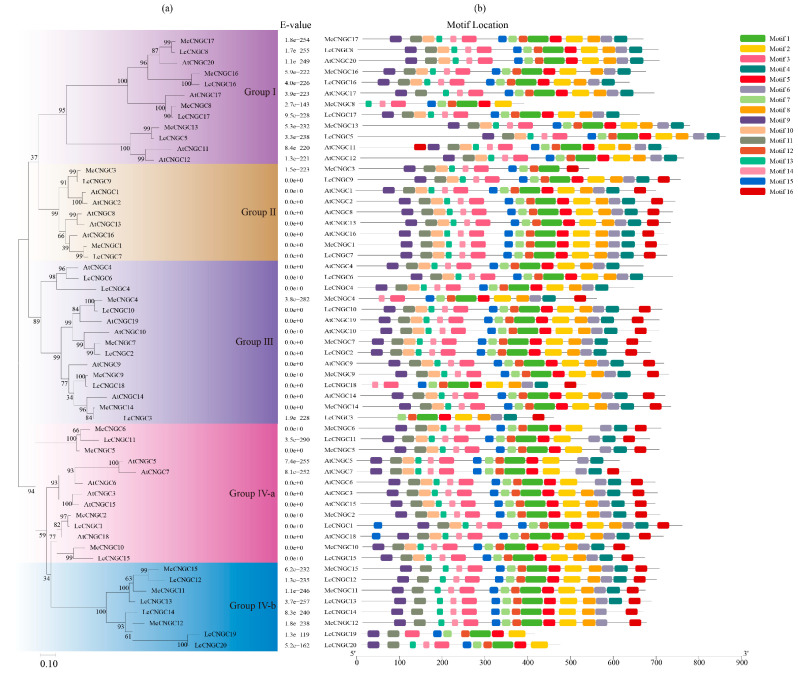
Phylogenetic tree and motif analysis of *CNGC* genes in *L. cylindrica*. (**a**) Phylogenetic tree constructed using the maximum-likelihood method using MEGA 7.0. (**b**) Motifs in luffa *CNGC* genes predicted using MEME; the sequence and length of each motif are shown.

A phylogenetic tree was constructed for the luffa *CNGC* genes according to the maximum-likelihood method using MEGA 7.0. As shown in Figure 3a, the tree contained five (Group Ⅰ, Ⅱ, Ⅲ, Ⅳ-a, and Ⅳ-b) subfamilies on the basis of the classification of the 20 AtCNGC, 17 McCNGC, and 20 LcCNGC protein sequences [34,47]. For each group of AtCNGCs and McCNGCs, the corresponding homologous genes were present in luffa, and the number of genes differed among the groups. Six LcCNGCs (LcCNGC2, LcCNGC3, LcCNGC4, LcCNGC6, LcCNGC10, and LcCNGC18) were clustered into group III, which formed the largest group. There were similar numbers of LcCNGCs and CNGCs from *Ziziphus jujuba* in Group III [9]. Group I contained four LcCNGCs (LcCNGC5, LcCNGC8, LcCNGC16, and LcCNGC17), and Group II contained LcCGNC7 and LcCGNC9. The remaining seven LcCGNCs belonged to Group IV-a (LcCNGC7 and LcCNGC9), and Group IV-b (LcCNGC12, LcCNGC13, LcCNGC14, LcCNGC19, and LcCNGC20). Some LcCGNCs were located on the same chromosome, such as *LcCGNC12* and *LcCGNC13*, and *LcCGNC14* and *LcCGNC15*. These two pairs showed little divergence and thus clustered into the same group, which might indicate that some segmental duplication of LcCGNCs has occurred during the evolution of *L. cylindrica*.

Divergences in protein motifs can provide insights into the evolutionary history of proteins [35]. Sixteen motifs in LcCNGCs were predicted with MEME (Figure 2b and Table 2). Except for LcCGNC14, LcCGNC19, and LcCGNC20, the other LcCGNCs all contained Motif 4 with the IQ domain ([RN]QWRTWAA[CV] FIQ [AL] AW [RH]RY) and Motif 8 with the CNBD. Motif 8 was located in the middle of the amino acid sequence. Motifs 1, 2, and 3 were TM domains located at the N- and C-terminals. These results indicated that the TM domain and CNBD are specific to plant CNGCs. Among them, the CNBD, which binds cAMP/cGMP, is the characteristic structure of plant CNGCs [48]. The CNBD contains two regions: the PBC and hinge regions. As shown in Figure 2b, a >90% conserved motif was found in the 20 LcCGNCs: [LIV] -X(2) -[GD] -[DG] -F-[CFYL] -X-G-[ED] -E-L-[LV] -X-W-X-[LM] -X(8)-[PD] -X(11)-[EQ] -[AP] -F-X- [LAF]. This motif contained the PBC and hinge regions. All of the LcCGNCs contained the conserved glycine (G) and aliphatic leucine (L) residues in the PBC motif. This conserved motif was consistent with those detected in CGNCs of *Gossypium* [35], *Solanum melongena* [14], and *Mangifera indica* [20].

### 2.5. Analysis of CNGC Gene Transcript Profiles in L. cylindrica

#### 2.5.1. Tissue-Specific Gene Transcript Profiles

**Figure 4:**
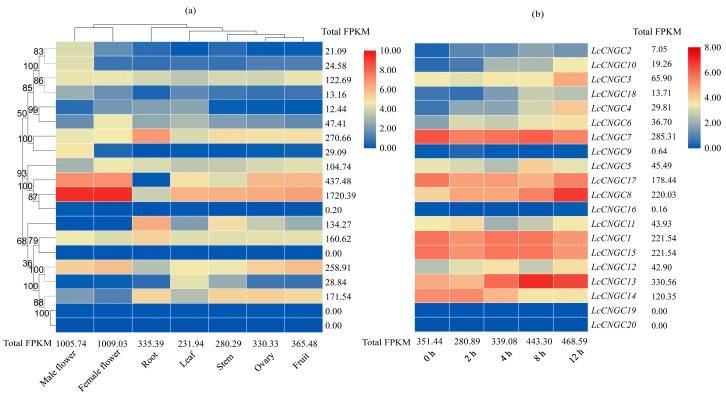
*CNGC* gene transcript profiles in *L. cylindrica*. (**a**) Tissue-specific transcript profiles of *L. cylindrica CNGC* genes. (**b**) *CNGC* transcript profiles in *L. cylindrica* in response to cold stress.

Analyses of transcript profiles of 20 *LcCNGC* genes in different tissues (roots, stems, leaves, male and female flowers, fruit, and ovaries) based on transcriptome data (Supplementary File S6) revealed the tissue-specific expression patterns of the *LcCNGC* gene family members. FPKM values determined from transcriptome sequencing data showed differences in *LcCNGC* transcript levels among the seven selected tissues. Specifically, the highest *LcCNGC* transcript levels were found in female flowers, followed by male flowers, fruit, roots, ovaries, stems, and leaves, with FPKM values of 1009.03, 1005.74, 365.48, 335.39, 330.33, 280.29, and 231.91, respectively (Figure 4a). However, among the 20 luffa *LcCNGC* genes, *LcCNGC15* and *LcCNGC20* had undetectable transcript levels in all tissues (FPKM value of 0). The FPKM values for two *LcCNGC* genes (*LcCNGC16* and *LcCNGC19*) were lower than 1 in the seven examined tissues, whereas those for seven *LcCNGC* genes (*LcCNGC2*, *LcCNGC4*, *LcCNGC6*, *LcCNGC9*, *LcCNGC10*, *LcCNGC13*, and *LcCNGC18*), they ranged from 12.44 to 47.41 in the selected tissues, with an average of 25.23. The FPKM values for the other 11 *LcCNGC* genes were also determined for all tissues. Three *LcCNGC* genes showed the highest transcript levels in the roots, especially *LcCNGC7* (FPKM value of 109.63) and *LcCNGC11* (FPKM value of 79.99). Four *LcCNGC* genes (*LcCNGC2*, *LcCNGC9*, *LcCNGC10*, and *LcCNGC17*) were predominantly expressed in male flowers (FPKM values of 17.22, 28.04, 18.56, and 130.00, respectively), and four were predominantly expressed in female flowers (*LcCNGC8*, *LcCNGC5*, *LcCNGC6*, and *LcCNGC3*; FPKM values of 699.74, 23.48, 23.46, and 22.20, respectively).

#### 2.5.2. Changes in CNGC Transcript Levels in *L. cylindrica* Exposed to Cold Stress

The *LcCNGC* transcript levels’ response to low-temperature stress was analyzed using Illumina RNA sequencing data (Figure 4b and Supplementary File S7). The results reflect the diverse effects of cold stress on *LcCNGC* expression. As shown in the heat map (Figure 4b), the transcript levels of the 20 *LcCNGC* genes in *L. cylindrica* varied among the selected time points (0, 2, 4, 8, and 12 h). The FPKM values indicated the *LcCNGC* transcript levels gradually increased, with FPKM values of 351.44, 280.89, 339.08, 443.30, and 468.59, at 0, 2, 4, 8, and 12 h, respectively (Figure 4b and Supplementary File S7). Notably, *LcCNGC8* and *LcCNGC13* showed the highest transcript levels at all time points during the low-temperature treatment, implying that they are highly responsive to cold stress.

**Figure 5:**
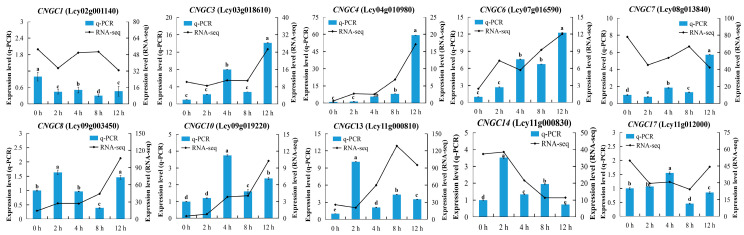
Quantitative real-time polymerase chain reaction (RT-qPCR) analysis of transcript levels (RNA-seq) of selected *LcCNGC* genes in luffa leaf under low-temperature stress. The *18s rRNA* gene was used as an internal control. Error bars represent the standard error of three biological replicates. Lowercase letters indicate RT-qPCR analyses’ significant differences (*p* < 0.05).

**Figure 6:**
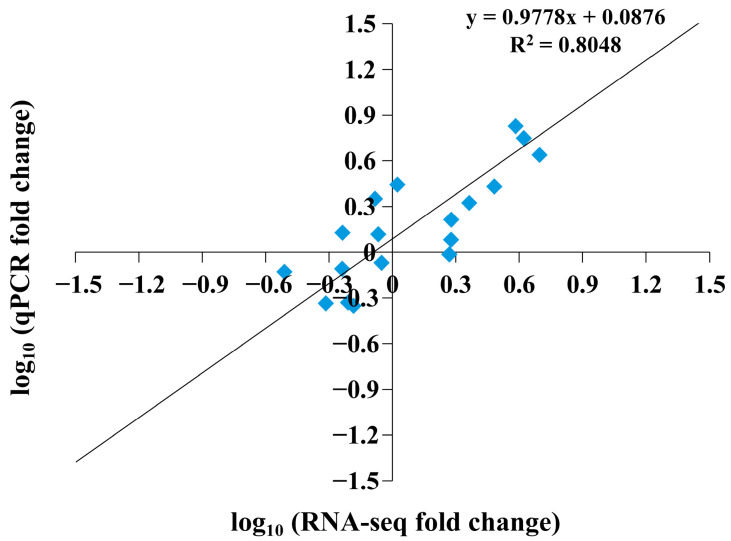
Regression analysis of fold-change values determined using RNA sequencing and RT-qPCR analyses. Regression analyses of transcript levels of *CNGC* genes after low-temperature stress as determined from RNA sequencing data and RT-qPCR analyses. For RNA sequencing data, the fold-change value was calculated as the ratio of the FPKM value for the stress-treated sample. For RT-qPCR data, the fold-change value was calculated by normalizing the transcript level in the stress-treated sample against that in the control.

To verify the results obtained from the transcriptome data, we used the Primer 5.0 software to design 21 pairs of RT-qPCR primers (Supplementary File S8, Table S2) to detect *LcCNGC* gene transcript levels in luffa leaves at 0, 2, 4, 8, and 12 h during the low-temperature treatment (Figure 5). As determined by RT-qPCR analyses, the transcript levels of *LcCNGC1*, *LcCNGC3*, *LcCNGC4*, *LcCNGC6*, and *LcCNGC13* gradually increased during the low-temperature treatment, reaching peak levels at 12 h. The transcript levels of *LcCNGC13* and *LcCNGC14* gradually increased during the first 2 h of the low-temperature treatment and then gradually decreased, and those of *LcCNGC10* and *LcCNGC17* increased to peak at 4 h and then gradually decreased. The RT-qPCR data for most of the analyzed *LcCNGC* genes were consistent with the results based on the transcriptome data, confirming the reliability of the transcriptome analysis. A correlation analysis (Figure 6) confirmed the strong positive correlation between the RT-qPCR results and those determined from the RNA sequence data (*R*^2^ = 0.8048).

### 2.6. Subcellular Localization of Luffa CNGC Proteins

**Figure 7:**
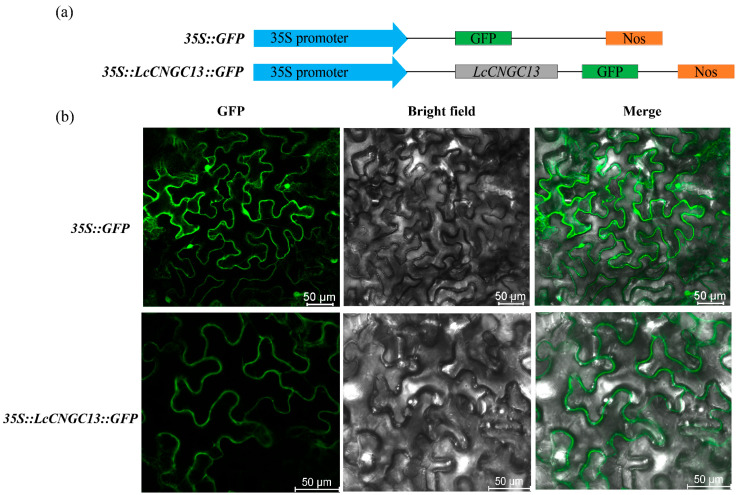
Subcellular localization of the LcCNGC protein. (**a**) Vector construction of pCAMBIA1300-*LcCNGC13-GFP*. (**b**) Green fluorescence, visible light, and merged green fluorescence and visible light images are shown. *35S*::*GFP*: *Agrobacterium tumefaciens* strain carrying the empty vector (pCAMBIA1300-*GFP*); *35S*::*LcCNGC*::*GFP*: *A. tumefaciens* strain carrying a recombinant vector (pCAMBIA1300-*LcCNGC13-GFP*). Scale bars = 50 µM.

To investigate the subcellular localization of LcCNGC proteins, we constructed transient recombinant expression vectors for LcCNGC13 fused with GFP. These constructs were then injected into tobacco (*Nicotiana benthamiana*) by *Agrobacterium*-mediated transformation and analyzed for transient expression and subcellular localization. In controls containing the empty vector (*35S*::*GFP*), green fluorescent signals were detected in the cell membrane, cytoplasm, and nucleus of the lower epidermis of tobacco, whereas in cells containing the recombinant vector for *35S*::*LcCNGC*::*GFP*, the green fluorescent signals were detected only at the plasma membrane. Accordingly, LcCNGC13 was localized to the plasma membrane, consistent with its predicted subcellular localization (Table 1 and Figure 7). The plasma membrane localization of this LcCNGC protein was in accordance with its role as a channel cation transporter.

## 3. Discussion

### 3.1. Characterization of the LcCNGC Gene Family in L. cylindrica

The *CNGC* gene family encodes channel proteins for calcium ions. These proteins have been reported to play important roles in the responses of plants, and their survival, under environmental stress [18]. Previous studies have shown that CNGCs are involved in resistance to low temperatures [26]. However, the luffa *CNGC* gene family had not been comprehensively identified or functionally annotated. Hence, we aimed to systematically characterize and clarify the evolutionary relationships of the luffa *CNGC* family members. The tissue-specific transcript profiles of the luffa *CNGC* genes and the effects of low-temperature stress on their transcript levels were determined on the basis of the luffa transcriptome data. The number of *CNGC* family members varies substantially among plant species. There are 20 luffa *CNGC* genes in luffa (this study); 20 in *Arabidopsis* [11]; 18 in tomato [49], 16 in rice [12], 30 in Chinese cabbage [15], 35 in tobacco [17], and 47 in wheat [10]. Differences in the number of *CNGC* gene family members among diverse plant species may be related to genome duplication events during evolution.

All the CNGC family members in luffa contained typical CNBD, CaMBD, and IQ motifs, except for LcCNGC19 and LcCNGC20, which lacked Motif 4 (the IQ domain) and Motif 8 (the CNBD) (Figure 3b). Similar to CNGCs in other plant species, members of the CNGC family in *L. cylindrica* were categorized into four groups, although the distribution among groups differed from that in other plants [13,22]. LcCNGCs in Groups II, III, and IV-a shared similar gene structures and patterns of conserved motifs, but those in Groups I and IV-b were more diverse in their structures and conserved motif patterns (Table 1). The high conservation of several motifs in LcCNGCs implies that they have similar modes of interaction with their target proteins. Subcellular localization analyses revealed that all 20 luffa LcCNGC proteins (except for LcCNGC3) localize to the plasma membrane, consistent with their putative function as channel cation transporters.

### 3.2. Phylogenetic Relationships and Evolution of LcCNGC Genes in L. cylindrica

The sequence alignment analysis showed that all the *LcCNGC* genes contained a highly conserved motif. Members of all four groups (Ⅰ–Ⅳ) contained the conserved motif ([RN]QWRTWAA[CV] FIQ [AL] AW [RH]RY), [LIV] - X(2) - [GD] -[DG] -F-[CFYL] -X-G-[ED] -E-L-[LV] -X-W-X-[LM] -X(8) -[PD] -X(11) -[EQ] -[AP] -F-X-[LAF]. This was only slightly different from the conserved motifs of CNGCs of other species, such as Gossypium ([LI] -X(2) -G -X-F-X-G-[ED] -X- L -L-X(25) -E-A-F- [AG] -L), wheat ([LI] -X(2) -[GS] -X -[FCV] -X -G -[ED] -E -L -L -[TGS] -W -X -[LF] -X(7,17) -[LFR] -[PL] -X-[SA]X(2) -[TS] -X(6) -[VAT] -[EQ] -X -F -X -L -X -[AS] -X -[DE]-[LV]), and rice ([LI ]-X(2) -[GS] -X-[FV] -XG-[DE] -ELL -X -W -X(12,22) -SA -X(2)-T-X(7)-[EQ]-AF-X-L) [10,21,35]. The high conservation of this motif among CNGCs in different species suggests that these proteins possess similar functions.

Gene duplication events can lead to the significant expansion of plant gene families. Tandemly repeated genes and fragment repeat genes play crucial roles in plant development and in responses to environmental stimuli [50]. We detected only one pair of replicated *LcCNGC* genes (*LcCNGC12/13*) resulting from tandem duplication, suggesting that tandem duplication events were not the primary contributor to the expansion of the *LcCNGC* gene family. We calculated *Ka*, *Ks*, and *Ka*/*Ks* values, and found that one tandemly duplicated *CNGC* gene pair in luffa had a *Ka*/*Ks* value of less than 1. This indicated that the duplicated gene pair *LcCNGC12/13* has been under strong purifying selection pressure during the evolution of the *LcCNGC* family. Thus, harmful mutations have been actively eliminated, resulting in relatively highly conserved gene functions.

### 3.3. Analysis of L. cylindrica CNGC Gene Promoters

*Cis*-acting elements in the promoter regions of genes can participate in the regulatory control of gene expression through synergistic interactions with the core promoter units of genes and also serve as important links between genes [51]. In the present study, analysis of the promoter of the *CNGC* gene of luffa revealed a number of *cis*-acting elements responsive to phytohormones and various stresses (Supplementary File S5), including elements responsive to hormones (ABA, MeJA, and salicylic acid) and environmental factors (light, drought, and low temperature). This suggested that CNGC proteins likely modulate luffa stress responses, as well as growth and development. Accordingly, the expression of *LcCNGC* genes may be mediated by plant hormone (e.g., ABA, salicylic acid, and MeJA) response pathways. In this study, low-temperature response elements were present in the promoter regions of several *LcCNGCs* (i.e., *LcCNGC1*, *LcCNGC2*, *LcCNGC8*, *LcCNGC11,* and *LcCNGC17*) (Figure 2a and Supplementary File S5). Moreover, MYB binding sites and W-box elements were also detected in the *CNGC* gene promoters, implying that *CNGC* genes are regulated by WRKY and MYB transcription factors. Both WRKY and MYB transcription factors can form cascades or networks with other functional genes to regulate the response to low-temperature stress [52,53].

### 3.4. Transcript Profiles of LcCNGC Genes in L. cylindrica and Changes Induced by Low-Temperature Stress

Analyses of gene transcript profiles can provide insights into gene functions [54]. We analyzed the tissue-specific transcript profiles of *LcCNGCs* in the roots, stems, leaves, flowers, and fruits on the basis of the available transcriptome data. The *LcCNGC* transcript levels (i.e., FPKM values) were lower in the leaves than in the other tissues (except for the stems) (Figure 4a). The transcript levels of some *LcCNGC* genes were affected by a low-temperature treatment. This transcriptional response suggests that there is functional differentiation among the *LcCNGC*s, with some of them playing specific roles in responses to various abiotic stresses. Studies in rice have revealed that *OsCNGC14* and *OsCNGC16* also play important roles in plant heat tolerance [27]. Under high- and low-temperature stress, rice mutants *cngc14* and *cngc16* showed significantly lower survival rates, and the expression levels of some high- and low-temperature-stress-related genes were altered in the *cngc16* mutant. In addition, deletion of the *OsCNGC14* or *OsCNGC16* genes significantly reduced or eliminated temperature-stress-induced cell membrane Ca^2+^ signaling. In Chinese jujube, *ZjCNGC4* was highly induced in response to cold treatments after 1 and 6 h [9]. In mango, *MiCNGC9* and *MiCNGC13* were found to be significantly upregulated by a low-temperature treatment (6 °C), suggesting that their encoded proteins participate in the response to cold stress.

After analyzing the transcriptome data, we selected 10 genes potentially affected by abiotic stress from the luffa *LcCNGC* family. To explore their possible roles in the response to low temperature, the transcript levels of these 10 *LcCNGC* genes during a low-temperature treatment were verified by RT-qPCR (Figure 5 and Supplementary File S5). There were clear differences in the expression patterns of these 10 *LcCNGC* genes. As determined by RT-qPCR, there was a gradual increase in *LcCNGC1*, *LcCNGC3*, *LcCNGC4*, *LcCNGC6*, and *LcCNGC13* transcript levels at 0, 2, 4, 8, and 12 h during the low-temperature treatment period, with peak levels at 12 h. The transcript levels of *LcCNGC13* and *LcCNGC14* increased soon after the start of the low-temperature treatment, peaked at 2 h, and then gradually decreased at the later time points. In eggplant, *SmCNGC1a* was significantly upregulated under cold stress (4 °C) [14]. Both *LcCNGC10* and *LcCNGC17* were significantly upregulated at 4 h of cold stress, suggesting that they encode proteins that play crucial roles in the response of young luffa seedlings to cold stress. In the present study, many LTR and abscisic acid response elements were detected in the promoters of *LcCNGC8* and *LcCNGC17* (Subfamily I); *LcCNGC4*, *LcCNGC6*, and *LcCNGC10* (Subfamily III); *LcCNGC1* (Subfamily IV-a); and *LcCNGC13* (Subfamily IV-b). The presence of these elements suggests that these genes play a role in the response of luffa to low-temperature conditions.

## 4. Materials and Methods

### 4.1. Plant Materials, Treatments, RNA Extraction, and RNA Sequencing

Seeds of the cold-resistant variety *L*. *cylindrica* Fusi-1 were sown in an incubator equipped with a LED cold light source. The seedlings were grown under the following conditions: 16 h day (28 °C)/8 h night (20 °C) cycle, 300 μmol photons m^−2^ s^−1^ light intensity, and 70% relative humidity. Fifteen-day-old seedlings were incubated at 25 °C (control) or 5 °C (low-temperature treatment) under light conditions (80 μmol photons m^−2^ s^−1^) for 12 h, with samples collected at 0, 2, 4, 8, and 12 h as previously described, and the 2nd true leaf was collected as material for subsequent experimental study, the 0 h samples without low-temperature treatment were used as controls (Supplementary File S9, Figure S1) [28,55]. Three biological replicates were prepared for all control and treated samples. At each time point, the collected leaf samples were combined, immediately frozen in liquid nitrogen, and stored at −80 °C until use.

Total RNA was extracted, mRNA was purified, and cDNA libraries were constructed by Guangzhou Gene Denovo Technologies Co. (Guangzhou, China). The cDNA libraries were constructed as previously described [56] and then sequenced on an Illumina HiSeq 2500 instrument (Illumina, San Diego, CA, USA). High-quality reads were aligned to the luffa cultivar P93075 (PRJNA596077) reference genome [43,57]. For RT-qPCR analysis, total RNA was extracted from each sample, then cDNA was synthesized using previously described methods [28,58].

### 4.2. Identification of CNGC Gene Family Members in L. cylindrica

The *LcCNGC* gene family members were identified on the basis of the reference genome of *L. cylindrica* cultivar P93075 and downloaded from the NCBI database (accessed on 27 June 2024) [43]. In addition, 20 *Arabidopsis* (*Arabidopsis thaliana*) and 17 bitter gourd (*Momordica charantia*) CNGC protein sequences were obtained from the TAIR database (https://www.arabidopsis.org/ accessed on 27 June 2024) and the bitter gourd genome database (https://www.ncbi.nlm.nih.gov/datasets/genome/?taxon=3673) (accessed on 28 June 2024) [47], respectively. Information related to the luffa genome assembly was downloaded to construct a local database. This information included *LcCNGC* genes, their promoter and coding sequences, and the amino acid sequences of their encoded proteins.

### 4.3. Bioinformatics Analysis of the LcCNGC Gene Family

All potential *LcCNGC* genes were confirmed by HMMER analysis, i.e., those with both the CNBD [cNMP_binding family] and the ITP domain [Ion_trans family] [21,49]. The protein sequences with six membrane-spanning regions, a pore region, the CNBD, and the CaMBD or the IQD motif were recognized as LcCNGC proteins [59]. The *LcCNGC* gene structures, promoter sequences, and *Ka*/*Ks* ratio for the duplicated genes [60] and the phylogenetic relationships, conserved motifs, isoelectric point, molecular weight, and subcellular localization of the putative LcCNGC proteins were analyzed as previously described [28,58].

### 4.4. Analysis of CNGC Gene Transcript Profiles in L. cylindrica Tissues

The tissue-specific *LcCNGC* transcript profiles were determined using the transcriptome data from Fusi-1 (PRJNA1044273) for the following tissues: roots, stems, leaves, flowers, and fruit [28]. StringTie2 software (v.2.1.5) was used to assemble sequences and calculate the fragments per kilobase of transcript per million mapped reads (FPKM) values. TBtools software (v.1.09876) was then used to visualize the gene transcript profiles in various tissues and in response to abiotic stress.

### 4.5. Analysis and Validation of CNGC Gene Transcript Profiles in L. cylindrica Under Low-Temperature Stress

The Fusi-1 (PRJNA1044273) tissue transcriptome data obtained in this study were used to examine *LcCNGC* transcript profiles in seedling leaves at five time points during a short-term low-temperature treatment: 0 h, 2 h, 4 h, 8 h, and 12 h [28].

The FPKM values were calculated using StringTie2 software (v.2.1.5). For the low-temperature treatment, the control plants were incubated at 25 °C, whereas the treated plants were incubated at 5 °C, with samples collected at 2, 4, 8, and 12 h. The transcript level of each gene was calculated relative to its corresponding transcript level in the control plants.

For the RT-qPCR analyses, primers (i.e., *LcCNGC*-Fq and *LcCNGC*-Rq) were designed according to the sequences determined in this study (Supplementary File S8). The RT-qPCR program used to amplify the internal control gene *Lc18S* rRNA and the calculation of relative gene transcript levels were as previously described [55,58].

### 4.6. Subcellular Localization of LcCGNC Proteins

To investigate the subcellular localization of an LcCGNC protein, the full-length sequence of *LcCGNC13* without the stop codon was amplified by PCR using the primers listed in Supplementary File S8. The *CNGC* gene sequence was introduced into the pCAMBIA1300 vector for expression as a green fluorescent protein (GFP) fusion protein. The generated recombinant plasmid pCAMBIA1300-*LcCNGC13* (*35S*::*LcCNGC13*::*GFP*) was introduced into the leaves of *N. benthamiana* seedlings for subcellular localization analysis. The empty vector (*35S*::*GFP*) was similarly introduced as the control. The leaves were examined to detect GFP fluorescence under a confocal laser scanning microscope as previously described [28].

## 5. Conclusions

In this study, we detected 20 *LcCNGC* genes and found that they were differentially regulated under low temperature. A comprehensive analysis of phylogenetic relationships, chromosomal locations, gene structures, gene duplication events, and conserved protein motifs was performed. On the basis of the tissue-specific and low-temperature-responsive *LcCNGC* transcript profiles, several *LcCNGC* genes (e.g., *LcCNGC3*, *LcCNGC4*, *LcCNGC6*, and *LcCNGC13*) were identified as candidate regulators of the response to low temperature in *L. cylindrica*.

## Figures and Tables

**Table 1:** *CNGC* family genes identified in *L. cylindrica*.

Gene Name	Gene ID	Position	Coding Sequence Length/bp	Protein Length/aa	Conserved Domains’ Location	Relative Molecular Weight/kDa	Theoretical Isoelectric Point (pI)	Group	Subcellular Localization
*LcCNGC1*	Lcy02g001140	Chr02: 1,532,606–1,539,547 (+)	2310	770	539–670	88.68	9.41	IV-a	Plasma membrane
*LcCNGC2*	Lcy03g014490	Chr03: 48,631,670–48,635,414 (−)	2079	693	438–569	79.51	9.09	Ⅲ	Plasma membrane
*LcCNGC3*	Lcy03g018610	Chr03: 52,325,676–52,332,121 (−)	1392	464	226–357	53.47	9.58	Ⅲ	Chloroplast
*LcCNGC4*	Lcy04g010980	Chr04: 42,360,506–42,365,299 (−)	1962	654	85–460	75.05	8.53	Ⅲ	Plasma membrane
*LcCNGC5*	Lcy06g000140	Chr06: 250,105–272,944 (−)	2610	870	698–816	99.48	8.89	Ⅰ	Plasma membrane
*LcCNGC6*	Lcy07g016590	Chr07: 45,284,867–45,293,470 (−)	2235	745	524–655	84.89	9.32	Ⅲ	Plasma membrane
*LcCNGC7*	Lcy08g013840	Chr08: 43,879,866–43,903,445 (+)	2196	732	156–554	83.93	9.27	Ⅱ-a	Plasma membrane
*LcCNGC8*	Lcy09g003450	Chr09: 2,996,960–3,002,381 (+)	2133	711	523–630	81.60	9.62	Ⅰ	Plasma membrane
*LcCNGC9*	Lcy09g018640	Chr09: 41,407,813–41,413,401 (−)	2292	764	539–670	87.60	9.51	Ⅱ-a	Plasma membrane
*LcCNGC10*	Lcy09g019220	Chr09: 43,242,303–43,247,104 (+)	2133	711	458–589	81.32	8.82	Ⅲ	Plasma membrane
*LcCNGC11*	Lcy10g017690	Chr10: 45,735,353–45,746,103 (+)	2052	684	101–522	78.84	9.31	IV-a	Plasma membrane
*LcCNGC12*	Lcy11g000780	Chr11: 777,524–780,531 (−)	2094	698	473–587	80.00	9.36	IV-b	Plasma membrane
*LcCNGC13*	Lcy11g000810	Chr11: 799,059–802,200 (−)	2058	686	465–581	78.38	9.43	IV-b	Plasma membrane
*LcCNGC14*	Lcy11g000830	Chr11: 807,730–812,348 (−)	1995	665	463–579	76.41	9.45	IV-b	Plasma membrane
*LcCNGC15*	Lcy11g000840	Chr11: 817,566–823,121 (−)	2010	670	443–562	77.05	9.32	IV-a	Plasma membrane
*LcCNGC16*	Lcy11g003110	Chr11: 2,989,382–2,993,545 (−)	1902	634	450–533	73.12	9.36	Ⅰ	Plasma membrane
*LcCNGC17*	Lcy11g012000	Chr11: 15,061,686–15,069,510 (+)	1974	658	466–566	76.02	9.28	Ⅰ	Plasma membrane
*LcCNGC18*	Lcy12g016140	Chr12: 44,065,281–44,067,927 (−)	1593	531	290–421	61.30	8.21	Ⅲ	Plasma membrane
*LcCNGC19*	Lcy13g014940	Chr13: 34,786,898–34,793,306 (−)	1230	410	346–407	47.27	9.02	IV-b	Plasma membrane
*LcCNGC20*	Lcy13g015690	Chr13: 42,524,448–42,526,976 (−)	1410	470	397–468	53.91	9.07	IV-b	Plasma membrane

Note: (+) and (−) indicate forward and reverse orientations, respectively, of the genes on chromosomes.

**Table 2:** Consistent sequences of predicted CNGC motifs in *L. cylindrica*.

Motif	*E*-*Value*	Sites	Width	Best Possible Match
Motif1	2.8 × 10^−1949^	57	50	IGNMQTYLQSTTVRLEEMRLKRRDTEZWMRHRQLPZDLRERVRRYEQYKW
Motif2	5.9 × 10^−1478^	56	41	MDEQLLDAICERLKPVLYTEGTYIVREGDPVBEMLFIIRGK
Motif3	1.4 × 10^−1298^	56	36	ETAWAGAAYNLLLYMLASHVVGAFWYLLSIERQASC
Motif4	5.2 × 10^−1075^	47	32	LHSKQLQHTFRFYSHQWRTWAACFIQAAWRRY
Motif5	4.1 × 10^−1031^	56	29	TRGVDEENJLQNLPKDLRRDIKRHLCLDL
Motif6	1.2 × 10^−831^	52	28	SSTRTVRALTEVEAFALRAEDLKFVASQ
Motif7	1.3 × 10^−716^	56	21	KYFYCLWWGLQNLSSLGQNLE
Motif8	4.9 × 10^−689^	42	31	FFNSITLKPGDFCGEELLTWALDPKSSSNLP
Motif9	9.6 × 10^−738^	53	29	PQDKFLQRWNKIFVJSCVIALFVDPLFFY
Motif10	3.1 × 10^−632^	42	29	SSRVFGRGELVIDPKAIAKRYLRSYFJID
Motif11	3.2 × 10^−677^	53	29	IVVTVLRTFIDVFYLJHIVLQFRTAYVAP
Motif12	9.3 × 10^−553^	57	21	IGEVLFAILIAISGLVLFALL
Motif13	3.6 × 10^−372^	53	15	AVLPLPQIVIWLVIP
Motif14	4.1 × 10^−351^	55	15	VLFQYIPRLYRIYPL
Motif15	3.2 × 10^−359^	56	21	BNTPFBFGIFLDALTSGVVSS
Motif16	2.9 × 10^−332^	37	29	AKTSGSSPSLGATJLASRFAANALRGVRR

## Data Availability

The transcriptome data are available in the NCBI database (project ID: PRJNA1044273). Further inquiries can be directed to the corresponding authors.

## Supplementary Materials

The following supporting information can be downloaded at: https://www.mdpi.com/article/10.3390/ijms252011330/s1.

## References

[1] Zhang Z., Wen Y., Yuan L., Zhang Y., Liu J., Zhou F., Wang Q., Hu X. (2022). Genome-Wide Identification, Characterization, and Expression Analysis Related to Low-Temperature Stress of the *CmGLP* Gene Family in *Cucumis melo* L. Int. J. Mol. Sci..

[2] Chen L., Zhao Y., Xu S., Zhang Z., Xu Y., Zhang J., Chong K. (2018). OsMADS57 together with OsTB1 coordinates transcription of its target *OsWRKY94* and *D14* to switch its organogenesis to defense for cold adaptation in rice. New Phytol..

[3] Duszyn M., Świeżawska B., Szmidt-Jaworska A., Jaworski K. (2019). Cyclic nucleotide gated channels (CNGCs) in plant signalling—Current knowledge and perspectives. J. Plant Physiol..

[4] Zhao J., Peng S., Cui H., Li P., Li T., Liu L., Zhang H., Tian Z., Shang H., Xu R. (2022). Dynamic Expression, Differential Regulation and Functional Diversity of the CNGC Family Genes in Cotton. Int. J. Mol. Sci..

[5] Tian W., Hou C., Ren Z., Wang C., Zhao F., Dahlbeck D., Hu S., Zhang L., Niu Q., Li L. (2019). A calmodulin-gated calcium channel links pathogen patterns to plant immunity. Nature.

[6] Moon J.Y., Belloeil C., Ianna M.L., Shin R. (2019). *Arabidopsis* CNGC Family Members Contribute to Heavy Metal Ion Uptake in Plants. Int. J. Mol. Sci..

[7] Schuurink R.C., Shartzer S.F., Fath A., Jones R.L. (1998). Characterization of a calmodulin-binding transporter from the plasma membrane of barley aleurone. Proc. Natl. Acad. Sci. USA.

[8] Hao L., Qiao X. (2018). Genome-wide identification and analysis of the *CNGC* gene family in maize. PeerJ.

[9] Wang L., Li M., Liu Z., Dai L., Zhang M., Wang L., Zhao J., Liu M. (2020). Genome-wide identification of CNGC genes in Chinese jujube (*Ziziphus jujuba* Mill.) and ZjCNGC2 mediated signalling cascades in response to cold stress. BMC Genom..

[10] Guo J., Islam A., Lin H., Ji C., Duan Y., Liu P., Zeng Q., Day B., Kang Z., Guo J. (2018). Genome-Wide Identification of Cyclic Nucleotide-Gated Ion Channel Gene Family in Wheat and Functional Analyses of TaCNGC14 and TaCNGC16. Front. Plant Sci..

[11] Gong J., Shi T., Li Y., Wang H., Li F. (2021). Genome-Wide Identification and Characterization of Calcium Metabolism Related Gene Families in *Arabidopsis thaliana* and Their Regulation by *Bacillus amyloliquefaciens* Under High Calcium Stress. Front. Plant Sci..

[12] Lee S.-K., Lee S.-M., Kim M.-H., Park S.-K., Jung K.-H. (2022). Genome-Wide Analysis of Cyclic Nucleotide-Gated Channel Genes Related to Pollen Development in Rice. Plants.

[13] Kakar K.U., Nawaz Z., Kakar K., Ali E., Almoneafy A.A., Ullah R., Ren X.-L., Shu Q.-Y. (2017). Comprehensive genomic analysis of the CNGC gene family in Brassica oleracea: Novel insights into synteny, structures, and transcript profiles. BMC Genom..

[14] Jiang Z., Du L., Shen L., He J., Xia X., Zhang L., Yang X. (2023). Genome-Wide Exploration and Expression Analysis of the CNGC Gene Family in Eggplant (*Solanum melongena* L.) under Cold Stress, with Functional Characterization of *SmCNGC1a*. Int. J. Mol. Sci..

[15] Li Q., Yang S., Ren J., Ye X., Jiang X., Liu Z. (2019). Genome-wide identification and functional analysis of the cyclic nucleotide-gated channel gene family in Chinese cabbage. 3 Biotech.

[16] Mao X., Wang C., Lv Q., Tian Y., Wang D., Chen B., Mao J., Li W., Chu M., Zuo C. (2021). Cyclic nucleotide gated channel genes (CNGCs) in *Rosaceae*: Genome-wide annotation, evolution and the roles on Valsa canker resistance. Plant Cell Rep..

[17] Nawaz Z., Kakar K.U., Ullah R., Yu S., Zhang J., Shu Q.-Y., Ren X.-L. (2019). Genome-wide identification, evolution and expression analysis of cyclic nucleotide-gated channels in tobacco (*Nicotiana tabacum* L.). Genomics.

[18] Zhang L., Cui Y., An L., Li J., Yao Y., Bai Y., Li X., Yao X., Wu K. (2024). Genome-wide identification of the CNGC gene family and negative regulation of drought tolerance by HvCNGC3 and HvCNGC16 in transgenic *Arabidopsis thaliana*. Plant Physiol. Biochem..

[19] Zhang N., Lin H., Zeng Q., Fu D., Gao X., Wu J., Feng X., Wang Q., Ling Q., Wu Z. (2023). Genome-wide identification and expression analysis of the cyclic nucleotide-gated ion channel (CNGC) gene family in *Saccharum spontaneum*. BMC Genom..

[20] Zhang Y., Li Y., Yang J., Yang X., Chen S., Xie Z., Zhang M., Huang Y., Zhang J., Huang X. (2023). Genome-Wide Analysis and Expression of *Cyclic Nucleotide–Gated Ion Channel* (*CNGC*) Family Genes under Cold Stress in Mango (*Mangifera indica*). Plants.

[21] Nawaz Z., Kakar K.U., Saand M.A., Shu Q.-Y. (2014). Cyclic nucleotide-gated ion channel gene family in rice, identification, characterization and experimental analysis of expression response to plant hormones, biotic and abiotic stresses. BMC Genom..

[22] Baloch A.A., Kakar K.U., Nawaz Z., Mushtaq M., Abro A., Khan S., Latif A. (2022). Comparative genomics and evolutionary analysis of plant CNGCs. Biol. Methods Protoc..

[23] Baloch A.A., Raza A.M., Rana S.S.A., Ullah S., Khan S., Nisa Z.U., Zahid H., Malghani G.K., Kakar K.U. (2021). *BrCNGC* gene family in field mustard: Genome-wide identification, characterization, comparative synteny, evolution and expression profiling. Sci. Rep..

[24] Kidokoro S., Shinozaki K., Yamaguchi-Shinozaki K. (2022). Transcriptional regulatory network of plant cold-stress responses. Trends Plant Sci..

[25] Peng Y., Ming Y., Jiang B., Zhang X., Fu D., Lin Q., Zhang X., Wang Y., Shi Y., Gong Z. (2024). Differential phosphorylation of Ca^2+^-permeable channel CYCLIC NUCLEOTIDE–GATED CHANNEL20 modulates calcium-mediated freezing tolerance in Arabidopsis. Plant Cell.

[26] Wang J., Ren Y., Luo S., Zhang X., Liu X., Lin Q., Zhu S., Wan H., Yang Y., Zhang Y. (2020). Transcriptional activation and phosphorylation of OsCNGC9 confer enhanced chilling tolerance in rice. Mol. Plant.

[27] Cui Y., Lu S., Li Z., Cheng J., Hu P., Zhu T., Wang X., Jin M., Wang X., Li L. (2020). Cyclic Nucleotide-Gated Ion Channels 14 and 16 Promote Tolerance to Heat and Chilling in Rice. Plant Physiol..

[28] Liu J., Peng L., Cao C., Bai C., Wang Y., Li Z., Zhu H., Wen Q., He S. (2024). Identification of *WRKY* Family Members and Characterization of the Low-Temperature-Stress-Responsive *WRKY* Genes in Luffa (*Luffa cylindrica* L.). Plants.

[29] Wang J., Wang Y., Wu X., Wang B., Lu Z., Zhong L., Li G., Wu X. (2023). Insight into the bZIP gene family in *Lagenaria siceraria*: Genome and transcriptome analysis to understand gene diversification in Cucurbitaceae and the roles of *LsbZIP* gene expression and function under cold stress. Front. Plant Sci..

[30] Meng D., Li S., Feng X., Di Q., Zhou M., Yu X., He C., Yan Y., Wang J., Sun M. (2023). *CsBPC2* is essential for cucumber survival under cold stress. BMC Plant Biol..

[31] Cheng X., Qin M., Chen R., Jia Y., Zhu Q., Chen G., Wang A., Ling B., Rong W. (2023). *Citrullus colocynthis* (L.) Schrad.: A Promising Pharmaceutical Resource for Multiple Diseases. Molecules.

[32] Tang L., He Y., Liu B., Xu Y., Zhao G. (2023). Genome-Wide Identification and Characterization Analysis of WUSCHEL-Related Homeobox Family in Melon (*Cucumis melo* L.). Int. J. Mol. Sci..

[33] Li H., Kong F., Tang T., Luo Y., Gao H., Xu J., Xing G., Li L. (2023). Physiological and Transcriptomic Analyses Revealed That Humic Acids Improve Low-Temperature Stress Tolerance in Zucchini (*Cucurbita pepo* L.) Seedlings. Plants.

[34] Abdel-Hamid H., Chin K., Moeder W., Yoshioka K. (2011). High throughput chemical screening supports the involvement of Ca^2+^ in cyclic nucleotide-gated ion channel-mediated programmed cell death in Arabidopsis. Plant Signal. Behav..

[35] Chen L., Wang W., He H., Yang P., Sun X., Zhang Z. (2023). Genome-Wide Identification, Characterization and Experimental Expression Analysis of CNGC Gene Family in *Gossypium*. Int. J. Mol. Sci..

[36] Saand M.A., Xu Y.-P., Munyampundu J.-P., Li W., Zhang X.-R., Cai X.-Z. (2015). Phylogeny and evolution of plant cyclic nucleotide-gated ion channel (CNGC) gene family and functional analyses of tomato*CNGCs*. DNA Res..

[37] Xie D., Xu Y., Wang J., Liu W., Zhou Q., Luo S., Huang W., He X., Li Q., Peng Q. (2019). The wax gourd genomes offer insights into the genetic diversity and ancestral cucurbit karyotype. Nat. Commun..

[38] Guo S., Zhang J., Sun H., Salse J., Lucas W.J., Zhang H., Zheng Y., Mao L., Ren Y., Wang Z. (2012). The draft genome of watermelon (*Citrullus lanatus*) and resequencing of 20 diverse accessions. Nat. Genet..

[39] Sun H., Wu S., Zhang G., Jiao C., Guo S., Ren Y., Zhang J., Zhang H., Gong G., Jia Z. (2017). Karyotype Stability and Unbiased Fractionation in the Paleo-Allotetraploid *Cucurbita* Genomes. Mol. Plant.

[40] Garcia-Mas J., Benjak A., Sanseverino W., Bourgeois M., Mir G., González V.M., Hénaff E., Câmara F., Cozzuto L., Lowy E. (2012). The genome of melon (*Cucumis melo* L.). Proc. Natl. Acad. Sci. USA.

[41] Montero-Pau J., Bao K., Reddy U.K., Bai Y., Hammar S.A., Jiao C., Wehner T.C., Ramírez-Madera A.O., Weng Y., Grumet R. (2018). The USDA cucumber (*Cucumis sativus* L.) collection: Genetic diversity, population structure, genome-wide association studies, and core collection development. Hortic. Res..

[42] Saensuk C., Ruangnam S., Pitaloka M.K., Dumhai R., Mahatheeranont S., de Hoop S.J., Balatero C., Riangwong K., Ruanjaichon V., Toojinda T. (2022). A SNP of betaine aldehyde dehydrogenase (BADH) enhances an aroma (2-acetyl-1-pyrroline) in sponge gourd (*Luffa cylindrica*) and ridge gourd (*Luffa acutangula*). Sci. Rep..

[43] Wu H., Zhao G., Gong H., Li J., Luo C., He X., Luo S., Zheng X., Liu X., Guo J. (2020). A high-quality sponge gourd (*Luffa cylindrica*) genome. Hortic. Res..

[44] Holub E.B. (2001). The arms race is ancient history in Arabidopsis, the wildflower. Nat. Rev. Genet..

[45] Bao F., Ding A., Cheng T., Wang J., Zhang Q. (2019). Genome-Wide Analysis of Members of the WRKY Gene Family and Their Cold Stress Response in *Prunus mume*. Genes.

[46] Chen H., Li X., Li F., Li D., Dong Y., Fan Y. (2022). Bioinformatics Analysis of *WRKY* Family Genes in *Erianthus fulvus* Ness. Genes.

[47] Cui J., Cheng J., Nong D., Peng J., Hu Y., He W., Zhou Q., Dhillon N.P.S., Hu K. (2017). Genome-Wide Analysis of Simple Sequence Repeats in Bitter Gourd (Momordica charantia). Front. Plant Sci..

[48] DiFrancesco D., Tortora P. (1991). Direct activation of cardiac pacemaker channels by intracellular cyclic AMP. Nature.

[49] Saand M.A., Xu Y.-P., Li W., Wang J.-P., Cai X.-Z. (2015). Cyclic nucleotide gated channel gene family in tomato: Genome-wide identification and functional analyses in disease resistance. Front. Plant Sci..

[50] Guan K., Yang Z., Zhan M., Zheng M., You J., Meng X., Li H., Gao J. (2023). Two Sweet Sorghum (*Sorghum bicolor* L.) WRKY Transcription Factors Promote Aluminum Tolerance via the Reduction in Callose Deposition. Int. J. Mol. Sci..

[51] Wang X., Liu H., Yu Z., Zhu W., Zhang L., Wang B. (2023). Correction to: Characterization of wheat Wrab18 gene promoter and expression analysis under abiotic stress. Mol. Biol. Rep..

[52] Mahiwal S., Pahuja S., Pandey G.K. (2024). Review: Structural-functional relationship of WRKY transcription factors: Unfolding the role of WRKY in plants. Int. J. Biol. Macromol..

[53] Wang X., Niu Y., Zheng Y. (2021). Multiple Functions of MYB Transcription Factors in Abiotic Stress Responses. Int. J. Mol. Sci..

[54] Zhang L., Fu J., Dong T., Zhang M., Wu J., Liu C. (2023). Promoter cloning and activities analysis of JmLFY, a key gene for flowering in *Juglans mandshurica*. Front. Plant Sci..

[55] Liu J., Wang B., Li Y., Huang L., Zhang Q., Zhu H., Wen Q. (2020). RNA sequencing analysis of low temperature and low light intensity-responsive transcriptomes of zucchini (*Cucurbita pepo* L.). Sci. Hortic..

[56] Zou Z., Yang J. (2019). Genomic analysis of Dof transcription factors in *Hevea brasiliensis*, a rubber-producing tree. Ind. Crops Prod..

[57] Langdon W.B. (2015). Performance of genetic programming optimised Bowtie2 on genome comparison and analytic testing (GCAT) benchmarks. BioData Min..

[58] Liu J., Wang Y., Ye X., Zhang Q., Li Y., Chen M., Wang B., Bai C., Li Z., Wen Q. (2024). Genome-wide identification and expression analysis of the WRKY gene family in response to low-temperature and drought stresses in *Cucurbita pepo* L. Sci. Hortic..

[59] Chen J., Yin H., Gu J., Li L., Liu Z., Jiang X., Zhou H., Wei S., Zhang S., Wu J. (2015). Genomic characterization, phylogenetic comparison and differential expression of the cyclic nucleotide-gated channels gene family in pear (*Pyrus bretchneideri* Rehd.). Genomics.

[60] Wang D., Zhang Y., Zhang Z., Zhu J., Yu J. (2010). KaKs_Calculator 2.0: A Toolkit Incorporating Gamma-Series Methods and Sliding Window Strategies. Genom. Proteom. Bioinform..

