# Supplementary material for: Genome-Wide Identification of the Cyclic Nucleotide-Gated Ion Channel Gene Family and Expression Profiles Under Low-Temperature Stress in Luffa cylindrica L
Source: Int J Mol Sci. 2024 Oct 21;25(20):11330. doi: 10.3390/ijms252011330 (PMC11508470; doi:10.3390/ijms252011330)
Supplement: Supplementary file 1 [file ijms-25-11330-s001.zip › Supplementary File S1.pdf]

>*LcCNGC1* Chr02:1532606-1539547 PREDICTED: cyclic nucleotide-gated ion channel 1

ATGCCCCTTTTTGGAATGTTAGGAAGGTTGTGTTCAACTTTACCATACAGTGGACTCTCTTACTTGAAGTTCT
CTAACTGTTTTCCCCTCTTTTTCAGGAGTTCCCTCAAGAAAGGCGTGTCAACCAAAGAAGTATTTGGACTGTA
TGACATTACAATTCAGATAGTTATCATGACTTATCTGCAGGAGAAGATTGTGAGGTTTCAGGACTGGAGTTCA
GATAAAACTTCAAGGGGGTTATATTCTGCAGACAACACTTTAAATACTGGAAAGAATGGGACAAGAGCTGA
CTTAGTTTCAGAAAAGCCTCTCAAAGAATTGGAAACTGGTTCTTACAGGATTAATAGAATAAAGAAATCCTT
GAAGTCGTCTTCTTTCAATAAGTTTATGTCCAGAGGTTTTGGAACCGGAAAAAAAGTACTTGACCCACAGG
GGCCCTTTCTCCAGAAGTGGAATAAGATATTTGTGTTGTCTTGTGTGATTGCTGTTTCATTGGATCCTTTGTTC
TTTTATGTCCCTGTGATTGATGATGAGAAGAAATGTCTTGGGTTGGACAAGAAGATGGAGATTACAGCCAGT
GTATTACGTTCATTCACTGACATATTTTATATATTGCATATTGTTTTTCAATTCCGTACGGGATTTATCGCTCCTTC
TTCTCGAGTATTTGGAAGAGGTGTTCTAGTTGAAGATGCTTGGGCAATTGCAAAGAGATATCTGTCATCATAC
TTTTTGATCGACATTCTTGCAGTCCTCCCACTTCCACAAGTAGTGATTCTGATAATTATTCCAAACATGAAAGG
CTCAAGGTCGTTGAATACTAAGGACTTGCTGAAATTTGTTGTTTTCTTCCAATATGTACCAAGGTTCATTAGAA
TCTATCCTTTATATAAAGAAGTTACAAGAACATCGGTACTCACTGAAACTGCATGGGCTGGAGCTGCATTTAA
TCTCTTTCTCTACATGCTGGCAAGTCATGTCTTTGGAGCATTTTGGTACTTGTTTTCTATAGAGCGGGAAACTA
CATGCTGGCAGAGGGCTTGTCATAATCATACTGGCTGTGTTTCTAATTCACTATATTGTGATGTCAGTCCTGGA
AACAACGCATTTTTAAATACTTCATGTCCTACAGTTGGTGGAGACAACCCACCTTTTGATTTTGGAATATTTCT
TGATGCTCTTAATTCTGGTGTTGTGGAGTCGATGGATTTTCCACAGAAGTTCTTTTACTGTTTCTGGTGGGGG
CTGAGAAATCTAAGTTCCCTGGGTCAAAATCTCCAAACAAGCACATACGTATGGGAAATTTGCTTTGCTGTTT
TCATCTCCATCTCTGGCTTAGTTCTGTTTTCATTTCTCATCGGGAACATGCAGACATATTTGCAATCCACCACTA
CAAGATTGGAAGAGATGAGGGTTAGAAGAAGAGATGCAGAACAATGGATGTCCCACCGTTTACTCCCTGAA
AGCCTCCGAGAGCGAATCAGGAGGTATGAACAGTACAAGTGGCAGGAAACCAGAGGTGTTGATGAAGAG
AATTTGGTTCGAAATCTTCCAAAAGATCTCAGAAGGGACATAAAACGCCATCTTTGTTTGTCTCTGCTCATGA
GGGTACCGATATTTGAAAAAATGGACGAACAATTGTTGGATGCTATGTGTGACCGTCTCAAGCCAGTACTATA
CACAGAAGAAAGTTACATCGTTAGGGAAGGAGACCCGGTAGACGAGATGATCTTCATTATGCGAGGCAAGT
TATTGTCTGTAACTACAAATGGTGGAAGAACCGGTTTCTTTAATTCTGAACACCTCAAGGCAGGTGATTTCTG
TGGAGAAGAGTTACTGACATGGGCTCTGGATCCACATTCCTCATCCAATCTACCAATTTCGACAAGAACCGT
GCGAACTCTTTCAGAAGTTGAAGCATTTGCTTTAAAGGCAGATGACTTGAAGTTTGTAGCCTCACAGTTCCG
ACGGCTTCACAGCAAGCAGCTACGGCACACTTTCAGGTTCTATTCACAGCAATGGAGAACATGGGCAGCGT
GTTTCATACAAGCAGCATGGCGTCGATACAGAAGAAAGAAGCATGAACAGGCCCTCTTGGAAGAAGAGAA
CAGATTAAAAGATGCATTGGCCAAGACAGGTGGGAATTCTCCTAGTTTAGGTGCCACCATCTACGCATCCAG
ATTTGCAGCTAACATCCTTAGAACGATTCGACGCACAAGCTCGCGGAAGGCCAGGATTCCAGAGAGAATAC
CGCCCCTGTTGCTTCAGAAACCAGCAGAGCCTGATTTTACCTCTGAAGAGAATAGTTGA

>*LcCNGC2* Chr03:48631670-48635414 PREDICTED: probable cyclic nucleotide-gated ion channel 16

ATGCATAACATTGCTTATTCTCGATTGGGCGGCCTTCAAAAATCCCTTTCTCTGTACCGGAAAGTGCCTTGGT
GGGATCAAATTCTCGAACCAGATTCCGATTTCGTCATCCGATGGAATCGCATCTTCCTCGTCACTTGCTTGATC
GCGCTCTTCGTCGATCCTCTCTACTTTTATGTCTCTGTCATCGGCGGGCCTGCTTGTATGCGATTCCACATCGA
ACTTTGCATCGTCGTCACTTTCTTCCGCTCCGTCGTCGATTTGTTCTCCCTCTTTCATATACTTATGAAGTTTCG
CACCGCCTTCGTTGCGCCCAATTCTAGGGTTTTTGGCCGTGGGGAGCTTGTTAAGGAGCCTAGGGATATTGC
TATGCGCTACCTCAAGAAGGATTTTGTCATCGATCTTGCCGCTACGCTTCCCCTGCCTCAGATTGTGATTTGGT
TCGTGATTCCAGCATTGAAAAACCCAACAGCGACCCACGCTAACCACACCCTTGCTCTGATTGTGCTCATTCA

ATATGCTCCCCGACTGTTTGTTATATTTCCTCTGAACCGACAGATCAATAAGACCACAGGAGCGATAGCCAAA
ACTGCGTGGGCTGGTGCAGCTTATAATCTCCTACTCTATTTGCTTGCTAGCCATGTTATAGGATCTGCATGGTA
TGTGGCATCCATTCAGCGCCAGGATGAGTGTTGGAAGTTACAATGTAGAAAGGAGATGAATACGACACATTC
TCCTTCTTGCAGCCCCCTTTTTCTGGACTGTGAAAGTTTGAATGATCCAGAACGACAAGCTTGGCTGAGAGT
TACTAGTGTTCTTACGAACTGTGATACGCTAAATGATGAAAAGAGTTTTGAGTTTGGGATGTTTGCTGATGCG
TTTACTGATGAAGTTGCTTCAGCAAACTTTTTCGAGAAGTACTTCTACTGCCTTTGGTGGGGTTTGAAGAGT
TTGAGTGCATATGGACAAAATTTAACGACGAGCACTTATGTTGGTGAAATTTTATTTAGCATTTTGATTTGCTC
AGCGGGGTTGGTCCTCTTCTCGCACCTCATCGGACAAGTGCAGAGCTATCTGCAATCCACGACTGCTAGACT
TGAGCAATGGAGAGTAAAACGAAGGGACACAGAGGAATGGATGACGCATCGACAATTACCTCTTCATTTGC
AAGAGCGTGTTCGTCGATTCGTTCAATATAAATGGATTGCAACCCGAGGTGTAGACGAAGAATCGATCTTGC
GCTCTTTACCTTTGGATCTTCGACGTCAAATTCAGAGACATCTTTCTCTTGCACTTGTTCGCCGTGTTCCTTTT
TTTGCACAAATGGATGCTCAGCTATTGGACGCCATATGCGAGCGTCTAGTCTCATCCTTGAACACTAAAGACA
CATTCATAACAAGGGAGGGAGATCCAGTAAATGAGATGCTCTTCATCATCAGAGGCCAACTAGAGAGCTCCA
CAACAAATGGGGGCAGGTCAGGATTCTTCAACTCCATCACTCTCAGACCGGGCGACTTCTGCGGCGAAGAG
CTACTAACATGGGCCTTAGTCCCATCCCCAAGCCTAAGCTTTCCGTCCTCAACGCGAACGGTGAAGTCGCTC
ACCGAAGTCGAGGCATTCGCACTTCGAGCAGAAGATCTCAAGTTTGTAGCTAGCCAGTTCAAGCGCCTCCA
CAGCAAGAAACTGCAGCATGCTTTCAGATACTATTCCCACCAATGGAGGACATGGGGATCTTGCTTCATACA
AGCTGCTTGGAGAAGATATGTGAAAAGAAAGCTAGCTATGGAGTTGGCAAGACAAGAGGAGCTCTTTTAC
ACAACCATCTTGGACCAAGAACAGAGCCATGGCAGTGAAATGGAAGACCAAGAAGATGGTCAAACCAGTG
GCTCCAAACCTAAAATTGCAAACAAACCCAAACATCTTGGAATAACAATGTTGGCTTCAAAGTTTGCAGCAA
ACACAAGAAGAGGGATTCACCAGAAGTTGTCTGCGCTTGAATCTGATGCCACCAGCTTGAAAATGCCCAAG
CTGTTTAAGCCTGATGAACCAGACTTCTCTGCTTTCCAACATGGAAGTTGA

>*LcCNGC3* Chr03:52325676-52332121 PREDICTED: probable cyclic nucleotide-gated ion channel 17

ATGACTGAGCGATGGCACAGCACCACCTCCAAGGCTCCAAGTCACCAACACAGTGTTGCGAGAAGATCGAT
GAAAAGGGTCAAGAGTCAAGAGGGGAGCTCCCAAAAGTCCAAAATCATTCTGAAGGTGGGAACTGGAGG
CGGTGCGGCGCCCTTCTACAGAAGGCTGGAGGGAGGGTCGTCTAAAGTGGATGGTTGGTGGAAAAGCTTA
AAGTTCATGAGAAAGTTAGCTGCTCTTAAGCCTATTCTGAGAGAGTTGAACTTTGGAGTGTTAGGTGATACT
CTTTTCAGGGAGTGGGAACAGGATTTCAGTATCTTATGTTCATATGGACAGAATTTGGAAACAACCACTTTCA
TTGGTGAGACACTGTTTGCTGTGCTTATTGCCATCTTAGGCCTTGTATTATTTGCCCATCTGATTGGGAATATG
CAGACATATCTGCAATCCCTCACTGTGAGGCTTGAAGAATGGAGACTAAAGCGAAGAGACACTGAAGAGTG
GATGAGACATCGTCAACTCCCTGAAGACTTGAAACGACGTGTTCGACGGTTTGTACAATATAAGTGGGTAGC
AACTCGAGGAGTTGATGAAGAAGCCATCTTGCAGAGCTTGCCTGCTGATCTTCGACGAGATATTCAATGCCA
CCTATGTCTAGATCTTGTCCGAAGAGTCCCTTTCTTTGCGCAGATGGATGATCAGCTACTTGATGCAATGTGT
GAACGCCTAGTATCCTCCTTGAGCACCGAAGGTACCTATATTGTACGAGAGGGTGACCCAGTAACAGAGATG
CTGTTCATTATCCGAGGCAGATTGGAGAGTTCTACTACAAATGGAGGTCGGTCTGGTTTCTTCAACTCCATAA
TGTTGCGTCCTGGAGACTTTTGTGGGGAGGAACTACTTTCTTGGGCCTTGCACCCAAAATCCACCACCAACT
TGCCCTCCTCAACTAGAACAGTCAGAGCGCTCAATGAAGTTGAAGCTTTTGCACTCCGCGCTGAAGATCTTA
AATTTGTGGCCAACCAGTTTAGACGTCTGCACAGTAAAAAGTTGCAGCACACATTCCGCTTTTACTCTCATCA
TTGGAGAACATGGGCAGCTTGCTTTATTCAGGCTGCTTGGCGTCGCCATAAGAAAAGGATGATGGCAAAAG
ATCTCTTGATGAAGGAATCATTTACATTATCTGAAGAAGTGGCTGATGAAACTGGCCAAGGAGAAGGGGAA
CAATTTTCTGTTGTTTCAAATCCTTCTCAGACAAAAATGTATCTGGATGTAACCTTACTGGCTTCAAGGTTTGC
TGCAAACACAAGGAGAGGTGCTCAGAGAATGAAAGATGACTTGCCAAAGTTACAGAAGCCCGATGAACCC

GACTTTTCCATTGAGCCGGATAGATAA

>*LcCNGC4* Chr04:42360506-42365299 protein CNGC15b-like

ATGGCTGCTTCTCGTAGATGGAGATCAGTCACAGTCAGATTCCAAGAGGAGGAGCTCGAAAGCGGAGAAG
AAGAATCGTGGAGAGTGTTTCCGGAGGATTACGACGATGGAATGGTGTTGGATCCAAGGATGGCGATATTG
AACAGATGGAACAGGGTTTTTCTGGTGGCTTGTTTGGTGTCGCTGTTCGTGGACCCGGTGTTCTTCTTTCTG
CCGGTGGTGAAGGCGGAGGAGGGGTGCGTGGAAATGGGCGGAGGGCTCGCGGTGGCGCTGACGGTGGT
GCGGTCGGCGGCGGATGCGTTCTACATAACGCAGATTCTGGTGAGGTTTAGGACGGCTTACGTGGCTCCGT
CGTCGAGGATCTTTGGGAAAGGGGAGCTCGTCATTGATCCTTCCAAGATTGCTGCTAATTATCTTGTTTTCCA
CTTTTGGCTTGATTTTGCTGCTGCATTGCCACTTCCTCAGGCATTCATATGGGTAGCAATCCCAAACATCAGG
GGTTCAAACATGACCCCATGGTGTTACTTCCTTCGCTTCTCCATCCTCTTCCAATACCTCTTGAGGCTCTATCTC
ATCTTCCCTTTATGTGACCAAATCATTAATGCCACTGGAGTTTTGATGAAAACAGCATGGGCTGGAGCTGTTT
ATAATCTAATGCTCTTTATGCTAGCAAGCCACGTTTTGGGTTCGTGTTGGTACCTGTTATCCATTGGACGACAA
ATGGAATGTTGGAAAAAGGTTTGCCATTTGGGGCACTTGGATTGTCAATATGAGTTTTTTGATTGTAAAGCT
GTGGGAAATCCAAATAGAGCTGCTTGGTTTGAAGCAAGCAATATCTCAAATCTTTGCCATCCAACTGCCACC
ACTTTCTTCCATTTTGGGATCTTTTCTGATTCTTTTGCCTCCACTTCTTCCACCTTCTTCAGTAGGTACTTCTATT
GCTTTTGGTGGGGCTTGAGGAATTTAAGCTCTTTGGGACAAAATTTGCTGACGAGTAGCAACGTGGCGGA
AATCAACTTCGCGATTGTGATAGCGATCGTGGGATTGGTGCTTTTCGCACTTCTAATCGGAAATATGCAAACT
TATCTTCAATCGACAACTTTGAGGCTGGAAGAGTGGAGGGTGAGGAGAACGGACACAGAGCGATGGATGC
ACCACAGGCAGCTGCCGTATGAGCTGAAGGAAAGCGTTAGGAAATACGACCAATTCAGATGGATTGCGACT
CGTGGGGTTGATGAAGAAGACATTCTTAAAGGCCTTCCTATGGACCTTAGACGAGACATCAAACGCCATCTT
TGCCTCGACCTCGTTCGTCAGGTGCCACTGTTCAACGAGATGGAGGAGAGAATGTTGGACGCAATATGCGA
AAGATTGAGACCCTGTTTAAGCACGTCCAACACCTGCTTGGTCCGCGAAGGCGACCCAGTCAACGAGATGC
TCTTTATTATCCGCGGCCATCTCGACTCCCACACCACCAACGGCGGCCGCACCGGCTTCTTCAACTCCAGCCG
CCTCGGCCCCACCGACTTCTGCGGCGAGGAGCTTCTCCCCTGGGCACTCGACCCCCGCCCCGCCGCCGTCC
TCCCCTCCTCCACTCGCACCGTCAAGGCCATCACCGAAGTCGAGGCCTTCGCCCTCATCGCCGACGACCTCA
AGTTCGTCGCCGCCCAGTTCCGCCGCCTCCACAGCAAGCAGCTCAGGTACACTTTCAGGTTCCACTCCCACC
AGTGGCGGACCTGGGCCGCCTGTTTTATTCAGGCCGCGTGGTTCAGCTACAAGCGGCGGAAGGAGGCGGC
GGAGGTGAAGAAGGAGATTGTTGTTGGAAGGAAGTATGCGGCCAATTGTAGGAGGGTTGTCGGCCGTGG
TGGGAGTGGGCGGCGTTGCGATGGTGGTGAGATGAATGCCGTTCATGGGTCGCTGTTGAAGCCTATTGAAC
CTGACTTCTCGGTTGAAGAAAGATGA

>*LcCNGC5* Chr06:250105-272944 PREDICTED: probable cyclic nucleotide-gated ion channel 20, chloroplastic isoform X1

ATGAGTATGATCTATGAGCGTCAACTACAAAGAACAGTTGACGAGACAAAGGAACATCGACGGAAGGCTCC
TTTGGCTGGAAGACAAAATAATAACGAGAATGTTCAAAGTCTTCGCAGTCGCTGCGTCGCCTTGGTTAGCTC
TCTTACTATTACAATATCGTCTTCCCCTCGCAATGCAGTTGCAGACTTCTTCATCTGTTCACACTTTCACTTTCT
CCTCCTCTCTGCTGCCCTGGATTTCGCTTCGGATTCGAGTACGGAGTCTAGATACTTAGAAATGGCTGCTTTT
GAAAAAGACGATATACCAATGCTATCAAACACTGATCCACCATTGTTGGATGAAGAAGTGGATTCTTATTTTC
CTTCATATGCATCATTGGGGCGGAGTTCATCGCTATCAATTCCAACAACTTCTTCTGGGATGTATGGTAGTGAA
GCAAATCTTGTTGGTTATACTGGTCCCCTACGAAGTGAAAGAAAAAGTTCATTCATAGTGAATGGTTCAAAAT
ATACTGGCCATAAATCTGAAAAACTTTCACAGTCAAATCCAGTTGTGACTGAAAGCAAAACTGCAGAGCAAT
TGGCAGACAAATTTCCTTCTTCCAAGACAAAGGATGAGCCTGATTGGTACATTCATAACTATGCTGGAAGAA
ATGAACACTTAATAAGGTCTGGCCAGCTGGGAGTGTGCAATGATCCTTTCTGTATAACTTGCCCAACATATAA

TTTCAAAGCATTGCAACAGAAAAGCTCAAGAACGGCCGGCATATTTGACCCTGTGTTTCATAACGATCTCTAT
GGAGAAGGGAAAGGTTGGGCAGGGAAGTTTCGGTCCTTCTGGTGCTTCTTGCTTCCATATATTCCTGGAGT
TATGAATCCTCATGCAAAAGTTGTTCAGCAATGGAACAAGTTTTTCGTCATCTCTTGCTTAGTGGCGATCTTCT
TGGACCCATTGTTTTTCTTCTTGCTTGCTGTCCAAGAGGAAAATAAGTGCATATTTATCGATCGGACAATGAC
CACAACATTGGTGGTTTTCCGAAGTGTGACTGATTTTATCTATTTCCTACACATGCTTCTCCAGTTTAGGTTGG
CTTACGTGGCACCAGAAACCAGAGTTGTTGGTGCAGGCGAATTAGTTGACCATCCGAAGAAAATTGCTATG
AATTACCTTAAAGGAAATTTTTTTATAGATTTGCTTGTTGTTTTGCCGCTGCCTCAGATAATAGTTCTTCTAATC
CTGCCAAAGTCTTTGGGGTCATCTGGAGCAAATTATGCAAAAAATCTATTACGCACTGTAGTTTTGGTTCAAT
ATATTCCCAGATTGTATAGGTTTCTACCTCTTCTTGCTGGCCAGTCTCCAAGTGGCTTTGTATTTGAGACAGCA
TGGGCAAATTTTGTCATCAATCTTCTCACCTTTATGTTGGCTGGTCATATTGTTGGGTCTTTGTGGTATCTCTTT
GGGCTTCAGGGGGTGAATCGATGTTTTCGACGTGCCTGCAATGACACTGGAACTACGAGCTGCTTAGAATAT
ATAGATTGTGGGCATGGGTACGAAGATGAGAGTCAAAATGAGCTTCCACTGCGGAAAGCATGGAAAGAAA
ATCCAAATGCTACGGCATGTTTTGCTAAAGAAGGTTTTGATTATGGAATCTACCTTCAGGCTGTAAATCTTACC
ACAGAAAATAGTATAATCACGAGATATACTTACTCATTATTTTGGGGATTCCAGCAAATCAGTACTCTGGCTGG
GAACCAAGTTCCAAGCTATTATGTCTATGAAGTCCTTTTCACTATGGGTATTATTGGGCTTGGTCTATTGCTGT
TTGCCCTTCTCATCGGTAATATGCAGAACTTTCTTCAGTCTCTTGGAAGAAGGAGGTTAGAAATGTCACTAAG
ACGTCGTGATGTTGAGCAATGGATGGAACATCGTCGCTTGCCGGAGAATTTAAGAAGGCAAGTACGACAGG
CTGAAAGGTACAATTGGGCTTCTACAAGAGGTGTAAATGAAGAAAGGATTTTCGAAAATTTACCTGAGGAC
CTTCAAAGAAACATAAGACGACATCTCTTTAAATTTGTCAGCAATGTTCGAATTTTTGCACTGATGGACTATG
AGCCTATTTTAGATGCAATACGTGAGAGACTGCGTCAAAAGACATACATTGAAGGAAGTGAAGTTTTTTCTG
CTGGAGATATCATCGAGAAGATGGTTTTCATTGTACGGGGGAAAATGGAGAGCCGTGTAGATGGGACTGGG
ATTGTGGTTCCCTTAATTGAAGGAGATGTCTGCGGTGAGGAACTTCTAACATGGTGTCTTGAGCATTCCTCCA
TAAACAGAGATATGAAGCGACCTCAAATTCCAGCACAAAGATTGGTCAGCAACCGGACCGTAAAGTGCTTAT
CAAATGTTGAAGCATTCTCTCTCCGGGCTGCGGACCTTGAAGAAGTGACCAGTATGTTTTCTAGATTCTTGC
GAAATCCACGCGTTCAAGGAGCCATAAGGTACGAGTCACCCTACTGGAGGTACCTAGCGGCAACGCGTATT
CAAGTAGCTTGGAGATACAGGAAAAGGCGTCTATCTCGAGCTCAAACCTCTCAATCTAACGACTAA

>*LcCNGC6* Chr07:45284867-45293470 PREDICTED: putative cyclic nucleotide-gated ion channel 15 isoform X1

ATGGACCCAGTTTCTGTTTTGGACTGTCTCTCTGTCGCTCTCGTTCCGTCTCTTTCTCATACTCATGGAAGCGC
TTCTTCTGGGTTGATTCCATTAGAACTGCAGCCAAAGGTTTCATTTGGGAAGATCTTTATGGCTTATGGTAGT
TCTAGATCCGTAAGATTCCAAGATGATCTTGAATCATCTACCCTGCCTACTATCAATGGTGGTGGTGTGAAGAA
GATCATATACAACATTGATGGCTCACAAATACCGGAATCGAGCGGCAAGAGAACTGAGGTTTCTGGGAAGT
CTGGAAGATCCTTGAGAGCGAAAGTTTTGTCTAGAGTTTTCTCTGAGGATTATGAGAGAGTGCAGAGAAAA
ATATTGGATCCTAGGGGACAAGCCATAAGGCGATGGAATAAGATATTTTTAGTAGCTTGTTTAGTCTCTTTGT
TTGTAGACCCGCTTTTCTTTTACTTGCCTGTGGTCCGGAATGAGGTCTGCATCGATATAGGAGTAGGTCTCGA
AGTCGTCCTCACAACTATAAGATCAATAGCTGATGTTTTCTACACAATTCAGATTTTCATTAAGTTCCGTACAG
CTTATGTTGCGCCCTCTTCTCGTGTATTTGGGAGAGGAGAGCTTGTTATAGATCCTTGTAAGATAGCAATAAG
GTATCTACGCCATGGTTTCTGGATTGATTTCATTGCAGCCGTGCCAGTTCCTCAGGTACTGATCTGGATTGTCA
TCCCCAACCTTAGTGGTTCAACAATGACAAACACCAAGAACTTCCTAAGGTTCTTTCTCATATTCCAGTACCT
GCCGAGGCTTTTTCTCATATTTCCTCTCTCAACACAAATTGTTAAGGCCACTGGACTTGTGACACAAACAGCA
TGGGCTGGAGCTGCTTATAACCTGATTCTTTATATGTTGGCAAGCCATGTTTTAGGAGCCTGCTGGTACCTTC
TTTCAATCGAGCGACAAGAAGCTTGCTGGAGAAGGCTTTGCAAGTTTGACAAATCTTGTAAAGATGGATTC
TTCGACTGTCATAAGGCCGATGACCCTCAAAGGGACTCATGGTTTAAGACAAGTAACATTACGAGTTCCTGC

AATCCAAATGATCCCTTTTATCAATTTGGTATATACGGTGATGCGATAACTTTTGATGTCACAACCTCACCATTT
TTCAACAAGTACTTCTACTGTCTTTGGTGGGGCCTGAAAAATCTGAGTTCCTTGGGGCAAAATCTAGCCACA
AGCACTTTTGTTGGGGAAATAATATTTGCCATAATAGTGGCAACTCTTGGACTGGTTTTGTTTGCATTGCTCA
TTGGCAATATGCAGAAATACCTCCAATCGACGACTGTTCGGTTAGAAGAGTGGAGAATAAGGAGAACTGAT
ACAGAACAATGGATGCATCACAGGCAACTACCTCCAGAGCTCAGACAATCTGTCCGCAGATATGATCAGTAC
AAGTGGGTAGCAACTCGAGGAGTCGATGAAGAAGCCCTGCTTAGAAGCCTTCCATTGGACCTACGAAGAG
ACATAAAACGTCATCTTTGCCTCGACCTCGTTCGAAGGGTTCCATTGTTTGATCAAATGGATGAAAGAATGCT
AGACGCAATATGCGAAAGGCTCAAACCTGCCTTGAGCACAGAAGGCACATTTCTCGTGCGAGAAGGCGAC
CCGGTCAATGAAATGCTATTCATAATCAGAGGTCATCTCGATTCATACACCACTAATGGCGGTAGAACTGGAT
TTTTCAACTCCTGTCGTATCGGCCCAGGTGATTTCTGTGGTGAGGAACTGCTGACTTGGGCTCTCGATCCTCG
TCCAAGCGTTGTACTTCCTTCTTCCACTCGGACGGTCAAAGCCATATCTGAAGTAGAAGCATTTGCTCTCATA
GCAGAGGACTTGAAATTTGTATCCTCCCAGTTCAGAAGACTCCACAGCAAACAACTCAGACACAAGTTCCG
ATTCTACTCACATCAATGGCGAACATGGGCTGCGTGCTTTGTCCAAGCAGCGTGGCGGCGATATAAGAGACG
TAAGGAAAGAGCTGAGCTCAGGGCTAGAGAGAGCTATCCAGCTACGAAGCTCGAGTCCCCACTTTCACCAC
AGACTGCATCAAACGTGGATGCACGTTCAGGAAACGCTAGAAGAGGCATGAATAAGCGTTGTGCGTCGGAT
GCTGGAGTCGTAAGCTCGTTGCAGAAGCCAGAAGAGCCTGACTTCTCCATTGTTGAGGAATGA

>*LcCNGC7* Chr08:43879866-43903445 probable cyclic nucleotide-gated ion channel 5

ATGTTTGATTGTGGTTACAAGTCACAACTGATGGGTGGTCAACGAGAAAAGTTCGTGAGGTTGGATGACTT
GGACTCTAGATTGTCATCCCCATCAGATAGTGGAATGAGAAGATGTGGTTTTAATATAGATGGATTTAACCGT
GCAGTCCATGGAAGTGATAAGCCATCTGGATCCTTTAAGAGAGGAATGAGAAAAGGGTCCGAAGGACTTAA
ATCAATCGGCCGATCACTCAAGTTTGGGGTTTCTCGAGCTGTATTTCCAGAGGATCTTAAAGAGTCAAAGAA
GAAGATCTTTGATCCTCAAGACAAATTGCTCTTATTTTTGAATAAACTTTTTGTCATATCATGTATTTTGGCAAT
ATCTGTGGACCCACTATTCTTTTATGTTCCTGTCATTAACCAAAACTCAAACTGTCTTGGCATTGATAGAAAGC
TGGCAATTACAGTGACAACATTGCGGACAATTATTGATGTTTTCTATCTTATTCATATGGCTCTCCAATTCCGGA
CAGCTTATATTGCTCCATCATCTCGGGTGTTTGGGAGAGGTGAACTTGTGATTGATCCTGCACAAATAGCAAA
GCGATACTTGCGGCGGTATTTCATCATTGATTTGGTGTCTGTGCTGCCTCTTCCACAGATTGTGGTTTGGAGA
TTTCTTCAGAGGTCAAAAGGTTCAGATGTATATGTGACTAAGCAGGCCCTTCTTTTAATTGTATTCCTTCAATA
TATCCCAAGATTTCTTAGAGCGTTTCCATTGGCTTCAGAATTGAAAAGGACAACAGGCGTATTTGCAGAAAC
TGCTTGGGCAGGTGCTGCATATTATTTGCTATTATATATGCTTGCAAGTCATATAGTTGGAGCACTATGGTACTT
ATTGGCTGTGGAACGCAATGATTCATGTTGGCAGAAGTATTGTATAGCCCCGAGTTGCAGGAAAGATTTCTT
ATATTGTGGTAACCAAGACATGGAAGGTTATGCAACTTGGAACAGGACAAGTGTGGAGTCAAGTTGCAAAC
CTGCTGATGATAATCAACTGTTTGATTTTGGAATCTTTCAACAGGCTTTGTCCTCTGGCATTGCAGCATCCAA
GGATTTTATCAACAAATATTGTTACTGTCTATGGTGGGGCCTACAAAACTTAAGTACGCTTGGGCAGGGGCTT
CAAACAAGCACGTATCCGTGGGAAGTTATCTTTTCCATAGCATTGGCGGTACTTGGTCTCATTCTCTTTGCGC
TTCTTATTGGCAACATGCAGACTTATCTTCAATCTCTAACTATAAGGCTTGAGGAAATGAGGGTGAAAAGACG
TGATTCGGAGCAGTGGATGCATCATCGCTTGCTGCCACAAGAACTTCGGGAGAGAGTCAGGCGTTATGATC
AGTACAAGTGGTTGGAGACCCGTGGGGTGGACGAACAGAGTTTGGTTCAAACTCTACCTAAGGATCTTAGG
AGAGATATTAAACGGCATCTTTGTCTGGCTCTAGTAAGAAGGGTTCCTCTGTTTGAAAGCATGGACGAGAG
GCTGCTTGATGCTATCTGTGAGCGGCTGAAACCATGTTTGTTTACGGAGTACACTTACATAGTGCGTGAAGG
TGATCCAGTTGATGAGATGCTTTTCATTATACGTGGTCGGCTCGAGAGTGTGACTACGGATGGTGGGCGGAG
TGGATTTTTCAACCGTACCTTTCTGAAAGAAGGTGATTTTTGTGGTGAAGAGCTTTTGACCTGGGCTCTGGA
TCCAAAATCTGGTTCGAATCTTCCATCTTCTACAAGGACTGTAAAGGCTATTACAGAGGTTGAGGCTTTTGCC

CTTGTTGCCGAAGAGTTGAAATTTGTGGCCAGTCAGTTCAGGCGCCTTCACAGTAGACAGGTGCAACATAC
CTTCCGTTTCTACTCTCAGCAATGGAGAACCTGGGCTGCTTGCTTCATCCAAGCGGCATGGCGTCGGTATTCC
AAGAGAAAGTCTATGGAACTTCGTCAAAAGGAAGAAGTTGCAGCCGAAGAATCTGAGGGATCTCAAACTG
CCACAAGCGGAGGCTCTTATAGTATTCGGGCAACTTTCTTAGCCTCCAAATTTGCTGCCAATGCTCTTCGGGG
CGTTCAGCGATATCGGAATGCAAAGAGTGCTCAGGAATTGATAAAACTACAGAAGCCTCCAGAGCCAGATT
TTAGCGCTGATGATGCCGATTGA

>*LcCNGC8* Chr09:2996960-3002381 cyclic nucleotide-gated ion channel 2
ATGCCTTCTCTCCCCAGTCTCCCCTTCTCCTCTCCCATGTGGACCCAGCTTTGCCTGAGGCGGACCTCCCCTC
CTCTCAATGCCCACCACGACCTTAACCTCACCGCCGCCGCCCCCGATGACAGCCCCATCACCACCACCGTCG
AGTGCTACGCCTGCACCCAAGTCGGCGTCCCGGCCTTCCACTCCACCAGCTGCGACCACGCCCACCAGCAG
CCCCAATGGGAGGCCTCCGCCGGCTCTTCCCTCGTCCCCATTCAGCCCACCAAGGCCTCCCCTCGCCGCCCC
GCCCCGCACCATCCTTCCGGCCGCTTCGGCACCGTTCTCGACCCGAGAACCAAGCGGGTCCAGAGATGGAA
CCGGGCTCTGCTATTGGCCCGTGGAATGGCCCTCGCCGTCGACCCTCTCTACTTCTACGCGCTCTCCATCGGA
CGCGGTGGGTCGCCCTGCCTCTACATGGACGGCGGATTGGCCGCCGTCGTCACCGTCCTCCGCACCTGCCTC
GATTTCGTGCACTTGTGGCACGTTTGGCTCCAATTCCGCCTCGCCTACGTGTCCAAGGAGAGCATGGTGATT
GGCTGCGGCAAACTCGTGTGGGACGCACGTGCCATCGCCTCTCATTACGTTCGTTCCTTCAAAGGCTTCTGG
TTCGATGCCTTTGTCATCCTCCCAGTTCCTCAGATTGTTTATTGGTTGGTTTTACCAAAACTAATCAGAGAAGA
GAGAATCAAACTTATAATGACAGTAATCTTATTAATGTTCTTGTTCCAATTCCTCCCAAAAGTCTACCACTCCAT
TATTCTAATGAGAAGAATGCAGAAGGTCACTGGATACATCTTTGGCACCATTTGGTGGGGTTTTGGCCTCAA
TCTCATTGCCTATTTCATTGCCTCTCATGTTGCTGGGGGTTGCTGGTATGTTCTTGCAATACAGCGAGTCGCTT
CTTGCATCCAGCAACATTGTGAGAGAAACAAGTGCAACCTATCTCTGTCTTGCGCCGAGGAGGTCTGTTATC
AGTTTCTCTCATCGGCTACGACGATTGGAAATTCGTGTGGTCGTAATTCGACTACTACATTTAGGAAGCCACT
GTGCTTGGATGTTAATGGTCCATTCGCCTATGGCATCTACAAATGGGCTCTTCCAGTCATTTCTAGCAATTCAA
TTGCTGTCAAAATCCTCTATCCCATCTTTTGGGGCTTAATGACTCTCAGCACCTTTGGCAATGATCTCGAGCCC
ACGAGTAATTGGCTGGAAGTATGCTTCAGTATTTGCACAGTGCTCAGTGGACTGCTGCTTTTCACTCTTTTGA
TTGGTAATATCCAGGTTCTTTTGCACGCCGTCATGGCAAGGAGGCGAAAAATGCAGCTGAGATGTCGAGATT
TGGAGTGGTGGATGAGGAGACGACAATTGCCGTCTCGTTTGAGACAACGAGTTCGACACTATGAACACCAG
AGATGGGCAGCCATGGGCGGGGAAGATGAGATGGAGCTAATCAATGATTTGCCTGAAGGTCTTAGAAGAG
ACATTAAACGTCATCTTTGTGTTGATCTCATCAGAAAGGTGCCTCTGTTCCAAAACCTGGATGAGCTTATTCTC
GACAACATCTGCGATCGAGTCAAGCCACTTGTATTCTCTAAAGATGAAAAGATCATCAGAGAAGGAGATCCT
GTTCCAAGGATGTTATTCATAGTGTGCGGCCGTATAAAACGTAGCCAAAGCCTGAGCAAGGGCATGACAGC
AACAAGTTTGATTGAACCAGGAGGATTTCTCGGCGACGAGCTGCTATCGTGGTGTCTCCGGCGCCCATTTCT
CGAGAGGCTTCCAGCTTCATCAGCTACATTTGTTTGCATTGAACCAACAGAAGCATTTGCCCTGAAAGCAGA
CCATCTGAAATACATAACCGATCACTTCCGCTACAAATTCGCCAACGAGCGACTTAAGAGAACAGCAAGATA
CTACTCTTCAAACTGGAGAACATGGGCTGCTGTGAACATACAATTTGCTTGGCGTCGCTACCGAAAGAGGAT
GAGACGTCCAGTGATAGCTGTGGTGGAAAATGGAAGCAATGAACGTCGGCTTCTGCAGTATGCTGCAATGT
TCATGTCATTCAGGCCACATGACCATCTTGAATAG

>*LcCNGC9* Chr09:41407813-41413401 PREDICTED: putative cyclic nucleotide-gated ion channel 8 isoform X1
ATGTTCGATTGTGGTGGATACAAGTCGCAGTACATCGGCGGTCACAAGGAGAAATTCGTGAGGTACGTTCTC
GAATTCCTCAACTCGATTAACATTCAATTTCTTCGTATCCATTCAAGAGATTTTTCATTCCATGGACCTCGCAG
GTTGGATGATTTGGATTCAAATTTGTCAGTGCCCTCTGGATCAAGCAAGATGAAGAAATGCCGATTCAACCT

AGAAGGCCTCCCACTGCCATTCATCAGCCGGGCGAAGCCTCGAAACGCGTCGAAATCGTTCCGAAAGGGA
GTCCAAATGAGCTCCGACGGAATCATGACCCTAGGCCGGTCGCTCCGCTCCGGCGTGTCCAGAGTCATCTTC
CCCGAGGATCTGAAAGTATCGGACCAAAAGATCTTCGATCCGCAGGACAAATCGCTCCTCTTCTGGAACAAG
CTGTTCGTCTTGTGCTGTATCCTCGCCGTCTCCGTCGATCCGCTGTTCTTCTACCTTCCGGTCTTCAACCATGC
CTCTTACTGCCTCGGAATGGACACTCAGCTCGCCGTCACCACCACCACCCTCCGTACGGCCATCGATGTGTTC
TACTTGATTCGGATGGGGTTTCAGTTCCGGACGGCTTACGTTGCGCCGTCGTCGAGGGTGTTTGGTCGCGG
CGAGCTTGTGATCGATCCGGCGGACATCGCTAGGCGGTATCTTCAGCGCTATTTCGTCGCGGATTTTCTCTCT
GTTTTGCCTCTCCCTCAGGTGGTGGTGTGGAGGTTTCTTCACAGGTCAAAAGGTTCAGAAGTGTTGGCAAC
CAAACAAGCATTGTTAAACATAGTGTTTCTTCAGTATATTCCCAGATTCATTAGATTTATACCTTTGAACATTGA
GCTTAAGAAGACGGCAGGTGTTTTTGCTGAAAGTGCTTGGGCTGGTGCTGCTTATTATCTCCTCTTGTACAT
GCTTGCCAGTCACATAGCGGGAGCATTTTGGTACTTGCTGGCAGTGGAGAGGAACGATGCATGTTGGCGAC
AAGCATGCAAAAGCAGTGGGAAGTGCAACATTAACTTTTTGTACTGTGGGAATAAGCACATGTCTGGATATA
AGGAGTGGAGAAATATTAGCGTTGATGTTCTCACAAAGAAATGCACTGCTATTGGAGACAATTTGCCATTCA
ACTATGGCATTTACACTCAGGCCATTGCCTCTGGCATTGTTCAGTCAAGGACTTTCTTCTCCAAGTTCTGCTAC
TGCTTGTGGTGGGGCTTGCAAAATTTAAGTACACTTGGTCAAGGGTTGCTCACCAGTACCTATCCTGGAGAG
GTTATCTTTTCGATACTAATTGCAATTTCTGGGCTCCTCCTCTTTGCACTCTTGATTGGAAACATGCAAACGTAT
CTCCAATCTCTAACAGTCCGGCTGGAAGAGATGCGCATCAAGAGGAGAGACTCTGAGCAGTGGATGCACCA
TCGGCTGCTTCCGCCGGACTTGAGAGAAAAAGTTAGGCGCTACGATCAGTACAAATGGCTTGAAACTAGAG
GAGTCGATGAAGAGAGCTTGGTTCAAAGTCTGCCTAAAGATCTTAGGAGAGACATTAAACGACATCTCTGTC
TCAATTTGGTCAGAAGGGTGCCTCTATTTGCAAACATGGACGAGCGTCTACTTGACGCGATATGCGAGAGGC
TAAAGCCAACACTATACACAGAGAATACATACATCGTCCGAGAAGGCGACCCAGTTGACGAAATGCTCTTCA
TAATCCGCGGCCGTCTCGAGAGCGTCACCACTGACGGCGGCCGGAGTGGCTTCTTCAACCGCGGCGTTTTG
AAAGAAGGCGACTTCTGCGGCGAGGAGCTTCTGACATGGGCCCTCGACCCCAAGTCCGGCGCCAACCTCC
CCTCCTCCACCCGTACCGTCCACGCCTTGACGGAGGTCGAGGCCTTCGCCCTGGAGGCCGAGGAGCTCAAG
TTCGTCGCCAGCCAGTTCCGACGGCTCCACAGCCGGCAGGTCCAGCACACGTTCAGATTCTACTCGCAGCA
GTGGCGGACGTGGGCGTCGTGTTTTATACAGGCGGCGTGGCGGCGGTACATAAAGCGGAAGATGGCCGAG
CTGCGGCGGAAGGAGGAGGAGGAGGAGGATGCGGCGGCTGCGGCGGCGTATAGCTCGTCGAGGCTTGG
GGCGACGATTTTGGCGTCGAGATTTGCGGCGAATGCGCTTAGAGGGCATCGGATGAGGAATGTGAGCAGC
GGGAAGAGCTTGATTAACTTGCAGAAGCCGTCGGAGCCTGATTTTTCTGTGTATAAAGGAGAGTGA

>*LcCNGC10* Chr09:43242303-43247104 PREDICTED: putative cyclic nucleotide-gated ion channel 18

ATGAATAGAATCATCCACTCGCCGGCGTCCACATTCCGCCCCTTTCGGCGAAACCTAATCGGCTCATCCTCCG
ATTCCGCCACGACCGCCGTAGCCGCCGCGGCCGCCGATGAACACCCGAACTACATCCTCCTCCGCTACCAAA
TCCTCGACCCCGACAGCGACATTGTAGCGCAATGGAACCGCGTGTTTCTAATCACATGCCTGATCGCTCTGTT
CATCGATCCGCTGTATTTCTACACGTCGTCGGTCAGCGGGCCGGCCTGCCTGACCACCCAGATGAATCTGGC
AGTGGTGATCACGTTCTTCAGAACGGTGACGGATCTGTTCTTCTTGTTGCACATGGTGCTCAAGTTTCGAAC
GGCGTATGTGGCTCCGAGTTCTAGGGTTTTTGGAAGAGGAGAGCTGGTTATGGATGCCAGAGCCATTGCTA
CGCGATATTTGAAGTCTGATTTTGTGATTGATCTCGCCGCTACTCTCCCCCTCCCACAAATAGTGATGTGGTTG
GTGATTCCAGCAACAAGACACTCCAGAGCAGATCATGCCAACAACACAATCGCTCTCCTTGTTCTGCTCCAAT
ATGTTCCAAGGCTGTTCCTCATTTTCCCTCTCAACCAACGTATCGTCAAAACTACTGGTGTTGTTGCCAAAACT
GCCTGGGCTGGTGCTGCCTACAATCTCATCCTCTACATGCTTGCCAGTCATGTGTTGGGATCTACGTGGTATTT
GTTGTCCATAGGGCGACAGTTCTCTTGCTGGAGTTCAGAGTGTGCAAAGGAGAATGCCTCTAAGGTTCTAA
CTTGCCTTCCCATCTACTTGGATTGTGCCAGTTTGAATAACACAGAAAGACAATATTGGCTTAATATCACTCAA

GTTACTTCCAAATGCGATCCGAGGAATAAAAATATAAAGTTCAAGTTTGGAATGTTCTCAGATGCTTTTACCA
ATGATGTTGCCTCCTCCCACTTCTTTGCAAAGTACTTCTATTGTCTTTGGTGGGGCCTCAGAAATTTAAGTTCA
TATGGACAAACTTTGGATACAACAACGTATATTGGCGAGACATTATTTTGCATATCAACCTGCATATTTGGCTT
GATACTATTTTCACAACTCATTGGCAACATGCAGACATGTTTGCAATCAATGACGGTGAGACTCGAAGAATG
GAGGATAAAGCGAAGGGACACCGAAGAATGGATGAGACGTCGCCAATTACCTCCAGATTTGCAAGAACGA
GTTCGTCGATTCGTTCAATATAAATGGGTGGCTACAAGAGGAGTTAATGAAGAATCCATATTGCGTTCGTTGC
CTATCGACCTTCGTCGTGAGATTCAACAACATTTGTGTTTATCACTCGTTCGTCGTGTTCCTTTCTTCTCACAAA
TGGATGATCAACTTCTTGATGCTATATGTGAACGTCTTGTCTCGTCCTTGTGTACCCAAGGAACCTACATTGTG
CGAGAGGATGATCCAGTTAACGAAATGTTGTTTATTATAAGAGGACAACTAGAGAGCTCGACAACAAATGG
CGGTCGATCAGGGTTCTTCAACTCCATCACACTAAAACCAGGTGATTTCTGTGGTGAAGAACTATTGACATG
GGCCTTGATGCCAAGTTCCAATTTAAATATGCCTTCTTCTACTCGAACTGTCCGAGCGCTCACAGAAGTTGAA
GCTTTCGCTCTTCGAGCCGAGGACCTCAAGTTTGTCGCTGGTCAATTCAAACGCCTACATAGCAAGAAACTA
CAGCATGCCTTTAGGTATTATTCCCATCAATGGAGGACATGGGGTGCCTGCTTGATTCAAGTAGCTTGGAGG
AGGCTTCAGAAGAGAAAATTGGCAAAGAAATTAGCATTGAGAGAGAGCTTATGCTACATAGATTCAATAGA
ACAAGATAACGAGTACGACATCGAGCTAGCAGAAGAAGACTATGACGACGACAGCGACGAAGATATGTCAT
CTACAGACTACATGAACAAAACTCAAAATCTTGGGGCCACACTTTTGGCTTCAAAGTTTGCTGCCAACACTA
GAAGAGGAATTAATCAAAAGGGGCAAACATCCAAGCCTTCCAGTTTGAAAATGCCAAAGTTGTTTAAACCA
GATGAACCTGATTTTTCTATGGATGGATGA

>*LcCNGC11* Chr10:45735353-45746103 cyclic nucleotide-gated ion channel 1-like
ATGGCCAAGTGTATGGGTTCCTGGAAACCAAAATCTGAAAGGTTTCCATATCCACCTGGTTTCTTTGTCAAAT
ATGAAGCTAAGCAAAAGAAAAGCAGACTGTCGCCAATATTGGAGAGCATCTCTATTGCTTGGAAGAAAACT
CTTGATCCACAGAAGCCATTTCTTCAGCAATGGAACAAGATATTTGTGCTCTCCTGTGTCATAGCAGTAGCTG
TGGACCCTCTCTTCTTTTACATCCCAGTGTTTGATGGACAGCATCAATGCCTAAGCATGGACCAACCCCTTAC
AATCGTTGCTTGCGTTCTTCGTTCATTCATTGACATATTTTATCTACTTCACATGATCTTTGAATTTCGAACGGG
TTTTCTGCCCTCTGGCTCTCATACATCAGCATTATTAGGAAGTGCTGACTTAATTGAAAATCTAGCAGCTATAG
CAAAGAGATACCTCTTTTCAAATTTCACGATTGATATCTTATCCATTCTCCCAGTGCCTCAGCTTTTGATATTAG
TTATCGTACCTGCATTGAAAGGCACGATTCCATTGAAGACAAAGAACTTTGTAAAGATTGCAGTTCTTTTGCA
ATACATTCCAAGGCTTTTTCGAATTTATCCATTATACACAGAAGCAACCAAAACTTCTGGCATATTGACAGAAA
GAGCCTGGAGTGGAGCTGCCTTCAATCTCCTTATTTATATGCTAGCTAGTCATGTAGTTGGTGCCCTTTGGTAT
TTGTTTTCAGTAGAACAACAAGCAAGATGCTGGCTCGAGGCATGTAAGAAAAATAACTGTACTAGCAAATTT
CTGTACTGTGGAGATCACAGCAGACAGGCATACCCGTTTATAGATGAGTATTGCCCTCATAAAGAGACTGAA
GATGACAATGTCTTCAACTTTGGGATGTATGTTGAAGCACTTAAGTTTCATTTAACTGAGACAACGAGTTTTC
GCCGCAAATTCATTTATTCCTTTTGGTGGGCCCTGCGAAACGTCGGCTCTAGTGGACAAAACCTTCAAGTAA
GCAATCACATGGGGGAGGTTTTTTTTGCTGTCTTCATTGCCATCCTTGGTTTGGTTTTGTTTGCCTTTCTCATA
AGCAATATTCAGAAATATTTGCAGTCAGCAACTGTTAAAATAGAGCAGATGAGAATTAACCGAAGAGACGCC
GAGCATTGGATGGCACATCGTATGCTTCCGGAGGACTTGAGGCAGCGTATTAGACGATACGATCAATACAAA
TGGCAGCTAAACAGGGGAGTTAAGGAAGAGGAACTCATTAGTAACCTTCCTAAAGACCTTAGGAGAGACAT
CAAACGCCATCTTTGCTTGGCCCTTCTCAAGAAAGTGCCACTTTTTAGCAGCATGAATAAGCAACTGCTAGA
CTCAGTGTGCGAATATCTCAAGCCTGTGTTATTCACAGAGAAGAGCTTCATTCTGCAAGAGGGTGACCAAAT
TGATGTGATGCTATTTATCATGAAAGGAAATCTAGCTGCCATTACTACTAATGGTTGGAAAAACGACCCTTGG
TCGGTCACTCTTAAGGCTGGCGACTTCTGCGGTGAAGAGCTAGTACAATGGGCAATGGATCCCACCTCTACT
TCTCTTCCCATTTCAACCAGAAAAGTTAAATCTCTAACTGAAGTTGAAGCCTTTGCTCTAAAGGCTAATGAGC
TGAAATCTGTAACTAGTCAGTTCCACTTCCAGCGCCTCAATAGCAAGCAATTCCAACTCTCAGTGAGGTTCTA

CTCTCATCAATGGAAAGTTTGGGCAGCCTATAAGATACAAGAAGCTTGGCATGATTACCGTGAAAGAAAGAA
GAGAGGGGGTGGAAATGGCAGGTTTCAAGATGCATTGGCTGAAACAGTTGGCGCCTCGGCGAGCTTCGG
CGCCACTCTTTATGCATCCATATTCATATCCCATCTACTGCAAGCCGTGCAGCGAGATCAACACCATCAAACTA
CCCAACTCACTCGAGTGATGACGCTACCGCCGCCTCCAAAGCCAGACGACGAGCAAGACAGGCCAAACTTC
ACCATCTTGAATCTTTAG

>*LcCNGC12* Chr11:777524-780531 cyclic nucleotide-gated ion channel 1-like isoform X2

ATGAAGAAGGAGCTAGCTATGAATGCTGAAGAAGATGATTCCATACGGATTCAATCTTGCTCTCGGGAGCGT
GAGATCGAGAAACTTTCCTCCCATGGGAGCAACTGCTCAGTGAAATCCAAGGGTACAGTGGGATCGGTTCC
CAAAAGATTTCTATCTTCTACTGATAAGCTTACGAGTTTTGGAAACATTCAGTTTGATGAAGAAGTCAACTGT
AAAGTCTTAAGCTCCATAAAAGCATATATTGAAGGAAACATGATAATCCTTCATCTTTGGAATGATTTACTTGT
CATATTGTGCGTAATGGCTACCATACTGGACCCTTTGTTTTGTTACACCCTAGTAGTTAACGAAGAGAGAAAC
TGTATTGGGTTTGACAAGAAGTTGAGGGTGATAGTTGTAGTTCTGCGTTCACTCATTGATTTTGGCTACATGA
TTATGATAATCTTTCATTTTCATATCGGTTATACAGCATCACATGATGCAAAAAGTAGAAGACTTTGTTCAACTG
CAAGGAGATATCTTTTATCTTACTCTACAGTTGACATTCTTACACTTCTCCCTATCCCACAGGTGATGGTTTTAT
TAGTCATTCCAGGATCAAAAGGTTCTCATTTTACAGCTGCAATTAAGTCGATGAAATTTGTTTTCATAATTCAA
TACTTGCCCAGAGTCTTTCGAGTACAATCATTCTTAAAGAAAGTTAGATGGAGTTCTGGCATTTTCCTCAATA
CTACTGGGATCAAAGCAATATTCAATCTCTTCCTGTATGTGCTTGCAAGTCATGTCTTTGGAGCCTTTTGGTAC
TTGTTTTCTGTTGAGCGCAGAGCAACTTGCTTGCAAGTAAGGTGTCATAGTCATCCATATTGCCCTAAAATGT
ACAACGACAACTCTGTTGAGAGCTTCTGTACGGATGCTTGCTCTGCAAAAGCATTATCAAATGACACAGCGG
CTTTCAATTTTGGAATATTTGATGATGCCTTTAAGTCTGGTGTTGTCTATACACCAGATTTCATATGGAAAATCT
CTTACTGTTATTGGTGGGGTCTGCAGAATCTAAGTTCTTTGGGCCAAGGCCTCAAAACTAGCAAGTATATATG
GGAAATTTACTTTGCAAGCACCCTCACTATGTCTGGCTTGGTATTGTTTGCATTTTTGGTTGGAAATTTGCAG
ACAATTCTACAGGCAACATTTGCAAGAGTTGAGGAATTGAGATCAAAGGGACACGATATTGAAATGTGGAT
GGCCTACCATTCGCTCCCACGCAATCTAAAGAAGCGGATCAAACGATTCGAGAAGTATAGATGGCATAAAAC
CCGAGGTGTTGATGTTGAGAACATTCTCCAAAATCTTCCTAGAGATCTTAGAAGGGACACAACAAGACATCT
TTGCTTAGGTGTATTAGCAAGTGTCTCAATGTTTCAAAACATGGACGAGAAGTTCTTGGATGCAGTCTTTGG
TTTTCTGAAGCCAATGCTATACATTGAGCATAACTTCATTGTTCGCGAAGGCGAGCCGTTAGACGAGATGATC
TTCATTGTTCAAGGCAAATTATGGATCTATTCCAAATCCAGTAAGGATGATGAAACATCATGTTCCTGGCCGG
GCACTCAGAGCCTTCAAAAGGGTGATTTCTTTGGAGAAGAGCTATTGAATTGGGTACTGCAAGATCCATTTT
TATCCACTGTCCCCATATCCACAAAAACTGTCGCTGCACACACCAAAGTAGAAGCATTTGTCTTAAGCGCAAA
TGATCTGCAGATCGTCGTCTCCAAATTCTGGTGGCTTTTCAGCCGAGAGTTCAGAAACGATCCGTTGTTTAA
AGAGCGATGGGCACCTTGGGCTGCTCTCGTCTTGCAAGCTGCGTGGCGCCGTTACTTCAAGAACAAGCGCG
AAAAAGAGAAAGAGAAATCTCAGTTAGGCTTGGTCACTAAAAGTGGGAATCAACCTTCAGTTACCACCGCT
GTTCATGTGGCTAAATTCGTGGTTGGTGCATTACATGCTTTGAATCGAAGACGAAAAGGAAAAGAGCGAAG
CAACAAGTTGCTAGAATCTAGTAAAGGCAATGGCTTGCCAGAACCCAGCAACGTTTAA

>*LcCNGC13* Chr11:799059-802200 cyclic nucleotide-gated ion channel 1-like isoform X6

ATGAATGGAGAAGATGATGCCATACGGATTCAGTCTTGCTCTACTGAACACAAGACCGAGGGACTTTCCTCC
CATGGGGAGATACGCTCGGCGAATTCCATGGGTACAGTGGGACCGGTCCCAAGAAGATTTGAATCTTCTTCT
AACATACTTACCAGTTTTGGAAACATTCATTTTGATGAAGAAGTCAAGAGTAAAGGCTTAAGCTCCATAAAA
AAGTCTATTGAAGGAAACCTGCTATTCCTTCATCTCTGGAATGATGTGCTTGTCATGTTCTGTGTAATTGCAAC

CTTACTGGACCCTTTGTTCTGTTACATCCTAGTAGTTGAAGAAGAGAAAAATTGTATTGGGTTTGACAAGAA
GTTGAGGATCACAGCAGTAGTTCTGCGTTCGCTCATTGATTTTGGCTACATACTTCTCATTGTCTTCCATTTTC
GAATTGGTTATACTGCGCCGAATGATGCAAATGATGGAAGACTGCTCACAATAGTTGGGAGATATCTTTTTTC
GTACTTTACAATTGACATTCTTGCCGTTCTTCCTCTCCCACAGGTGGTGATATTATTAGTCATTCAAGCAACGA
AAGGTTCTCACTTTACAGTCGCTATTAGGTCATTGAAATTTGTCCTTATAATTCAATATTTGCCAAGGGTCTTTC
GAGTATACTCATTCTTAAAGAAAGTTAGATGGAGTTCCGGCATTTTGCCCGATTCTGCTGGGGCCAAAGCTAT
ATTCAATCTCTTCCTGTATATGCTTGCAAGTCACGTCTTTGGAGCCTTTTGGTACTTGTTTTCCATTGAGCGCA
AAGCAACTTGCTTGCAAGTAAGGTGTCATAGTCATCCATATTGCCCTCGGAATTATAACACCTCTGCTGAGAG
ATTATGTATAGATAATTGCTCTGGAAAAGCATCATCCAATGACACACCAGCTTTCAATTTTGGAATATTTGAAG
ATGCCTTTAATTATGGCGTTGTCAGTTCGACTGATTTCATATGGAAATTCTCTTATTGTTACTGGTGGGGTCTG
CAGAATCTAAGTTCTCTGGGTCAAGGCCTCAAAACTAGTAAGCATATATGGGAAATTTACTTTGCAGTCTCCA
TAACCATTGCTGGCTTGGTATTATTTGCACTTCTAATAGGAAATTTGCAGACATTTCTACAGGCAACAATTGCA
AGACTGGAGGAAATGAGATTGAAGGGGCAAGATATTGAACTATGGATGGCCTACCATTCACTCCCACGTGAT
CTAAGGAAGCGGATCAAACAATATGAGAAGTATAAATGGCGGAAAACTAGAGGTGTTGATGTTGCAAACAT
TCTCCACAATCTTCCTAGAGACCTTAGAAGGGACACAACAAGACACCTTTGCTTACGTGCAATCAAAAGTGT
CTCAATGTTTCAAAACACGGACGAGAAGTTCTTGGATGCAGTTTGTGGTTATCTGAAGCCAATGCTATACATC
GAGCGTAACTTCATCGTTCGCGAAGGAGAGCCGCTGGACGAGATGATCTTCATAATCCATGGCAAGTTATGG
ATCTATTCCAACTCCAACAGAGGTGGTGAAACATCAGGTTCGTCTGAGAGCCTTCAAAAAGGCGATTTCTTT
GGAGAAGATCTTTTAAAATGGGTGTTGAAAGATCCAGTTTTATCCACTGTCCCCATATCAACAAAAACTGTCT
CTACACACACCAAAGTGGAAGCATTTGTTCTAAGTGCAAATGATTTGAAGAATGTCGTCTCCAAATTCTGGT
GGCTTTTCAGTCGAGAGCTCAGAAACGATCCCAACTTTAAAGAGCGATGGGCGCCATGGGCTGCTCTCGTC
TTGCAAGCTGCGTGGCGTCGTTACTTCAGGAGTAAGCGTGAAAGAGAGAGATCTCAGTTAAGCTTAGCCAC
TGAAAGTGGGAATTCACAACCTTTGGTCACAACAACCATTCATGCTTCTAGGTTCATTGCTCGTGCATTACAT
GCTTTGAATCAAAGACGAAAAACAAGAAATGGAAGCAATGATATGCCGGGACCTAGCAACAGCAAAGACT
TGCCAAAACATAGCAACGTCTGA

>*LcCNGC14* Chr11:807730-812348 cyclic nucleotide-gated ion channel 1-like isoform X2

ATGGAAGTCCAGAAGAGTCCAGACGGTTTGTATCAAAAGCGACTTTCGTCTGCTGGAGAAACACATTCACG
GGATTCACTGAGATCATTGAGATCGGTTTCAACAAACTTTGCCGCTAGTTTTGATAGGCTTACGAGTTTTGG
AAACATATATTTTGAACAAGAAGTTAGGAGTAAAGGCTTGGGATATCTTAAAAGTATTGTTGATAAAAACCCA
CTATTCCTTCATTTGTGGAACGAAATACTTGTCATGTTGTGTGTGATTGCTACCTCATTGGATCCTTTATTCTGT
TACATCCTGTTGGTTGATGAAGGCAAAAGATGTGTTGGACTTGACAAGAAGTTGAGGACAGTAGCTGTAATT
ATTCGCTCAATCATAGACTTCCTCTACATAATTCTCATTTTCTTTCATTTTCAATTTGGATATTCCTCATTTTATAA
TGCAAATCGTGACAATGGTGACAATTCTGATGATGGTGTTTGCACAAGAGCATGGAGATTCCTTTTATCCTAC
TTCACAGTTGACGTTCTTGCAGTTCTTCCACTCCCCCAGGTGGTGGTTTTAATTCTCATTCCAAGTTTGAAAG
GACACGATTTTATATATGCAGTGAGGTCATTGAAATATATTCTCCTCGTTCAATATTTGCCAAGGGTCTTTCGA
ATCTACTCATTCTTAAAGAAAGTTAGATGGACTTCCAACATTCTCCCAGAAACTGCTGGGGCTAAAGCTGCAT
TCAATCTCTTCCTTTATATGCTTGCAAGCCATGTTTTTGGAGCATTTTGGTACTTATTCGCTATAGAGCGAAAA
ACAACTTGCTGGGAAGGAAGCTGTCAACATTGCCCTCTAAACTGTAATTACGTTTTAGGCAATTTCAGTGCT
GACAGTGACAGTTTTTGCTCTGCAAAAGCTGAAAATGGGTCCAAAGCTTTTGATTTTGGAATATTCAAAGAT
GCTTTTCGTGTAGCGGAGTCGAGAGATTTCACCAGAAAATTTTCTTACTGTTATTGGTGGGGTCTGCAGAAA
CTGAGCTCTTTGGGACAAGACCTCAAAACTAGTGACCATTTGTGGGAAATCTACTTTGCAGTCACCGTTACC
ATTTCTGGCTTGATATTATTTGCGCTTCTTGTCGGAAATTTGCAGACATATTTGCAGTCAACCATTGCAAGATT

GGAGGAAATGAGATTGAAGGGGCAAGATATTGAGTTGTGGATGGCTTACCATTCTCTCCCATCAAATTTGAA
GAAGAAGATTAAAAAGTATGAAAGGTACAAGTGGCGAGAAACGAAAGGCGTTGACGTCGAGCTACTTCTT
CATAATCTGCCTAGAGACCTAAGAAGGGACACAAAACGACATCTTTGCTCCACGCCACTAAAAAAAGTTTCA
AATCTTCAAAACATGGACGAAAAGCTACGGGATGCAATCTGTGATTACCTGAAGCCAGTGTTGTACATAGAG
CGCAACTATATTGTTAAAGAAGGAGAGCCACTGGATGAAATGGTCTTCATCATTCGAGGCAAAGTAATGGTC
TATTCCAAAAGAGATGGTGAAGCTGCAGCTGGTTCCTCAGGCTCCAAGTGGCTTAGCAAAGATGACTTCTAT
GGAGAGGACCTTCTAGAGTGGGCACTACGCAATCCTACTTCGACCACCGTCCCCATATCTACCAAAACCATCC
GAGCGCACTCGAAAGTCGAAGCGTTTGTCCTCATGGCAAACGATTTGAAGACCGTGGTCTCGAAATTTTGG
TGGCTTTTCAGCAGAAACTCTCCAAGTTTGAAAGCAATATGGGCGCCATGGGCAGCTTTAGCTTTGCAGTTG
GCATGGCGCCGATACCATAAAAGCAAGAACGAGAAGAACAAGTCTGAATTGGCCATTGAAAGAAGGAATA
CTCAGGCTAATTTAACTGCTCCACTTCTTACCACTACACATATTGCCCGTGCTCTTCGTGCTTTCAAGCTAAAT
GGCAAAAAAGCACAAGCAGAATCCAGCAGAGTCTGA

>*LcCNGC15* Chr11:817566-823121 cyclic nucleotide-gated ion channel 1-like
ATGAATTCAGCTCTCCTCAGAGGGTTGGCATCTAGTTCTGATGGAACTAGAAACACTGAGAAGCTATTGAAA
TCTTCTTCCTCTAATAATCGCATGCCTGAAGGCTTGCACTCAGGGAAGAAAATTCTTGATCCACAAGGGCCTT
TTCTTCAAAGTTGGAACAAGATGTTTGTGCTGTCTTGTGTGATTGCTGTATCGTTGGATCCTCTGTTCTTTTAT
GTTCCTGTGATTGATAATAGAAGGAAGTGTCTTAGGTTAGATGAGAAAGTGGAGACTGTAGTCTGTATATTGC
GATTATTTACAGACTTATTCTATGTTGTGCATATTGTTTTTCAGTTTCGTACAGGATTTATTGCTCCCTCTTCCCG
AGTATTTGGAAGAGGTGTTTTAGTAGAAGATTATCGGGCGATAGCAATGAGGTATCTGTCATCATACTTTCTA
ATTGACATTTCTTCAGTCCTCCCACTTCCACAGGTGGTGATTCTGATAATCGTCCCTGTAATGGGAAGTTCGA
GACCGTCGATTACGAAGGACTTGCTGAAATTTGCTGTATTATGTCAACTTGTGCCAAGGTTTCTTCGAATCTA
TCCACTGTATAAAGAAGTTACAAGGACCTCGGGCATACTCATCGAAACTGCTTGGGCAGGAGCTGCATTTAA
TCTATTGCTGTACATGCTAGCCGGTCATATATTTGGAGCAACCTGGTACTTATGTTCCATAGAACGAGAAGCCC
AGTGCTGGCACGATGCTTGTAGCAAGCATCCTGGCTGTAACTCTACTTCCTTGCACTGTGATTACAATTCCAG
TGTTGGAGGGAACCTATTCCTCAATGTTTCTTGCCCAATTGAAAAGCCAAATGTAGGACATTTTAACTTCGGA
ATATTCCTTCAAGCCCTTCGATCTGATATTGTGGAGTCAGATTTTCCGAAGAAGTTCCTTCACTGCTTTTGGTG
GGGCTTAAGGAATCTAAGTTCTTTGGGTCAAAATCTCACTACAAGTACTTGCACATGGGAAAACTGCTTCGC
CATTTTGGTTTGCATCTCTGGCTTAGTGTTGTTTGCGTCTCTCCTAGGCAACGTCCAGATGTATTGGCGATCCA
CAAACACAAGGGTGGAGGAGATGAGAGTGAGAAGGAGAGATGTTGAACAATGGATGTCGCATCGCTTACT
CCCTGAGAATATGAGGGAGCGGGTTCGAAGGTATAAACATTACACGTGGCTAGAAACCAGAGGTGTTGATG
AACACAATCTGCTGCTTAATCTGCCTAGAGACCTCCAACGAGATATCAAGCGCCATCTTTGCTTGCCTTTGCTT
ATGCGAGTTCCAATGTTTGAAAAGATGGATGAACAATTGTTGGACGCGATGTGTGCCCGTCTCAAGCCAGTG
TTGTACACACAAGAAAGCTGCGTTGTTCGGGAAGGCGATCCAGTTGACGAGATGCTCTTCATCATGCGAGG
CAAGCTCCTAACCATGACTACAAATGGTGGGAGAACTGGTTTCTTCAACTCTGATTTCCTCATGGCTGGTGAT
TTCTGTGGAGAAGAGCTACTGACTTGGGCTCTAGACCCTCATTCCTCAACCAATCTCCCCATTTCAACTCGAA
CCGTCCGATCCCTGACAGAAGTCGAAGCATTTGCTTTTAAACCCGATGACTTGAAAGTTGTAGCCTCTCAATA
CCGCAGGCTTCACAGCAAGCAGCTGCGCCAGATATTCAGATTCTACTCGCACCAATGGAGAACCTGGGCAG
CTTGCTTTGTACAAGCAGCATGGCGGCGCCATCGAAGAAAGCAGCTAAGAGAGTCTCTCAGGGAAGAAGA
GAGTAGGTTGAAAGACGCATTGGCTTGTCTCGAGGGGCGGTCGCCAAGTTTAGGCGCCACCATTTATGCAT
CTAGATTCGCTTCTAACATGCTTCGCGCCATGAGAAGGAATGGTACTAGGAAAGCTAGGATGTCCATGCTGC
TTCAGAAGCCAGCAGAGCCTGATTTTACCTTGGAAGATAACAACACATAG

>*LcCNGC16* Chr11:2989382-2993545 cyclic nucleotide-gated ion channel 2-like

ATGATGCCCACAAAATTCAATACCACCCATGCCGAATTAGATGAATTCCAAGATGACGACCACCACCAAAATG
GCTCCTTCGCCGGCTCTTCACGGCGGCGCGTGCTCGACCCGCGCTGCGCATGCGTGCAGCGGTGGAACCG
CGTGGTTCTGCTGGCGCGTGCGGCATCTGTGGCGGTTGACCCGCTCTTCTTTTACGTCATTTTTCTGACCAA
GGCCGGCCCGCCGTGCTTCTACCGGGATGCGGCTTTCGCCGCCGTCGTCACCGCCGTGCGCACGTGCGTGG
ACATGGTGCACGTGTGCCACGTGTGGATGCAGTTTCGGCTGGCGTTTGTTTCGACGGCGTCGTTGGTGGTC
GGGAGTGGGCGGCTCGTGTGGGATGCACGTGCCATTGCTTCTCACTATTTGAGATCCTTCAAAGGCTTCTG
GCTCGATTTGTTTGTGATTATTCCTATTCCTCAGGTTGTGACATGGTTGATAGTGCCAAAACTATTGAGAGAA
GAAGAGATAAAAAAGATGATGAAAGTGGTGTTGGTGAGCTACCTATTTCAGTTGCTTCCAAAGCTTTACCAC
AGCATTTACTTGATGAAGAAGCTCCAAAAAGTCACTGGCTTCATCTTTGGCTGCATTTGGTGGCGCTTTAAC
CTCAATGTCTTTGCATATTTGATTGCTTCTCATGTTGCAGGAGGATGTTGGTATCTCCTCGCAACACAACGACT
AATCTCGTGCCTCGAACAACAATGCGAAAGAAGAAAAATGTGCAAACTTGCATTGTCTTGCTCTTCCAACTC
TCCCACATCAGGCTACTACAAGGGGGCAGGAAAATTTGGAGCTCCCCATGGTAATAACTTGACAAAGATCAA
GTCCTTATGCTTGGAGGTCAATGGACCCTTCAGCTATGGAATTTATGAGCCTGTTCTTTTGGTATTCTCTAGCA
ATTCTTTAGCTGTTAGAATTCTCTATCCAGTCTTCTGGGGATTATTGAACCTGAGTTCCTTTGGCAATGAACTT
GATCCCACTAGTAATTTGGTTGAAGTGATATTCAGTAGCTGCATCACACTTGCAGGGTTGGTGCTGTTTGTTA
CCTTAATTGGAAATATACAGATTTTCTTGCAAACTGTCATGGCGAGTGAGGAGAATATGCAAATACGGTTTCG
AGATATGAAGTGGTGGATGAGGAGGCGACAATTGCCCATTCATTTGAGGGAAAGGGTTTGCAATTTTGAAT
ATCAAAGGTGGGTAGCCATGGGAGGACTAGATGAGACTGAGCTCATCAAAGGACTGCCCGATGGCCTCCGC
CGAGACATCAAACGCTACCTTTGTTTAGACCTCGTTAAGAAGGTACCTTTCTTCCACATGCTAGATGATCTCAT
ACTCGATAACATTTGTGATCGCGTTAAACCGCTCTTGTACGCCGAAGGTGAAAAGATAGTAAGGGAGGGAG
ACCCTGTGCAAAGGATGGTGTTCATTGTTGAAGGATGCGTAGAGCGCAGCCAAGGCTTTGTAGGCACAAGT
TTGCTAAACTCCGGTGGCTTTTTCGGCGAGGAGCTACTCTCGTGGTGTCTCCGACTCCGACACCGTCGACAT
TTCGATGAGCTTCCAACCTCCTTGGCAACATTTTCATGTGTACAGCCAGTAGAAGCATATGGGCTCGACGCA
CTCGATCTCAAGTACATTACCGAACACTTTCGGTACAAGTTTGCGAGTGAAAAGATCAAGCGAACGATGCGC
TATTACTCTTCGAATTGGCGAACATGGGGAGCTGTGATCATTCAGTCTTCATGGCGTCGCCACCGGATACGAA
CTCGAGGTTACACGACACGAGACCGAACAGCTGACAATCGACTGAGGCAGTATGCTGCAGTTTTCTTGTCA
ATGAAGCCGAACGATCATCTCGACTAG

>*LcCNGC17* Chr11:15061686-15069510 cyclic nucleotide-gated ion channel 4

ATGGCCACCACCCCTTCCCATTCCCACCACCACACCGCCACCACCTCCGACGAAGAAGAAGAAGAAAAAGA
TTTAGAAGACGACCAATCCAACGGCGCTGCCTTCTGCCGGAACCTTTACGGAGTCGGGTCGGTTCTCGACC
CGAGAACCAAATGGGTCCGCGAATGGAACCGGGTCTTCCTCCTGGTCTGCGCGGGCGGGCTGTTCGTGGA
CCCTCTCTTCCTCTACACGCTCTCGATAAGCGAGTCCTGGATGTGCGTGTTCGTGGACGGCTGGCTCGCCATC
ACCGTCACCGTCCTCCGCTGCATGGCCGACGCCTTGCACCTCTGGAACATCTGGCTCCAGCTCAAGACCGCC
ACGAGATCTTCCTTCGCCGCCGCCGACAACACACGCCTCACTGATACAACCCCACGCGCCGTCGCTCTCCGC
TACTTCAAGTCCAAGAAAGGCTTCTTCTTCGATCTCTTTGTCATTCTTCCTCTTCCTCAGGTTCTATTATGGGTT
GTAATTCCTTGGATAATGAAAGAAGGATCAGTGACACTGGTGATGACAGTGTTGTTGATAGTATTTCTGTTTC
AATATTTGCCCAAACTCTACCACTCGGTTTGCCTCTTGCGGCGCCTCCAAAACCTCTCTGGCTACATCTTCGG
CACTGTTTGGTGGGGCATTGCTCTCAATCTCATTGCTTACTTTGTTGCTGCTCATGCTGCAGGAGCATGCTGG
TACCTGCTGGGGATACAAAGGGCAGCCAAGTGCCTGAAAGAGCAATGCAGGGCAACGAGCAGCTGTGGA
CTGAGATCGTTGTCCTGCAAAGACCCAATATTCTATGGGGCAACCGACATGAACATGGGCAGAGACAGAGC
AAGATTTGATTGGGCGAACAACAGGCTACCGAAATTCATGTGTTTGGACACTGCAGACAACTTTGATTATGG
AGCTTACAAATGGACTGTTCAGCTTGTTGTCAATCAGAGTCGCTTGGAGAAGATCCTTTTCCCCATCTTTTGG
GGCCTCATGACTCTTAGTACATTTGGCAACTTGGAGAGCACCACGGAATGGCTCGAAGTAGTCTTCAATATC

ATTGTTCTCACCAGTGGACTTCTCTTGGTCACCATGTTGATTGGGAATATCAAGGTGTTTCTGCATGCAACAA
CGTCAAAGAAACAAGGAATGCAGCTGAAGATGAGGAACCTCGAGTGGTGGATGAGGAAGCGACGCCTGC
CACAAGGCTTCCGACAGCGCGTTCGCAACTACGAGCGGCAGCGGTGGGCAGCGATGCGCGGTGTCGACG
AGTGCGAGATGATCAGAAACCTTCCGGAAGGGCTCCGGCGAGACATCAAGTACCACCTTTGCTTGGATCTG
GTCAGACAGGTTCCTTTGTTTCAACATATGGATGAACTTGTTCTTGAGAACATATGCGACCGTGTCAAGTCCC
TCATCTTCACCAAAGGAGAAACTATAACGAGAGAAGGAGACCCAGTGCAGAGAATGCTATTCGTGGTGCGA
GGCCACCTCCAAAGCAGCCAAGTCCTACGCGACGGCGTCAAGAGCTGCTGCATGTTGGGCCCAGGCAACT
TCAGCGGCGACGAGCTTCTGTCCTGGTGCCTCCGCCGCCCCTTCATAGAGCGCCTGCCGCCCTCCTCCTGCA
CTCTCGTCACTCTCGAGACCACCGAGGCCTTCGGCTTGGAGGCCGACGACGTCAAGTACGTAACCCAGCAC
TTCCGCTACACATTCGTCAACGACAAGGTCAAGCGCAGCGCCCGTTACTACTCCCCCGGCTGGCGCACTTGG
GCCGCCGTCGCCATCCAGCTCGCCTGGCGCCGATACCGCCACCGTCTCACTCTCACGTCCTTGTCGTTTATTC
GCCCTCGGCGGCCGCTCTCGCGGTGCTCTTCGCTGGGCGAGGATCGCCTCCGCCTCTATACGGCGTTGCTCA
CTTCTCCAAAGCCCAATCAGGATCACTTTGATTTTTGA

>*LcCNGC18* Chr12:44065281-44067927 PREDICTED: probable cyclic nucleotide-gated ion channel 14 isoform X1

ATGATATGGTTTATAATGCCAGCAATTAGAAGCTCTCATGCAGACCATACAAACAATACTTTGGTGTTAGTAGT
CCTGCTTCAGTATATTCCAAGATTTTACCTGATCTTCCCATTGAGTTCCCACATTATCAAAACTACTGGTGTTGT
CACAAAGACTGCTTGGGCAGGGGCTGCATATAACCTTGTATTGTATATGCTAGCTAGTCATATCTTAGGAGCA
GCATGGTATTTACTGTCTGTGGAGCGCCATGCCATGTGCTGGAAGTTCACCTGCAGGAGAGAATTCAGTCCT
ATGAAATGCCTTCTTGACTATCTGGATTGTGGTACTTTAGATTATGTAGACCGCAGGATATGGGAAGTAAATAC
TACTGTGTTTAGCCAATGTTCTCCGGACGAAGATGTCGTTTTCAATTACGGGATATTTGCCGATGCGATCACG
AAAAACGTCATCTCCTCCGGGTTCGTTCAGAAGTATTTCTACTGTCTATGGTGGGGATTACAGAACTTGAGTT
CCTATGGTCAGGGTTTGGAGACAACTACCTTCATAGGAGAGACTTTATTTGCTATCCTCATTGCTATAATGGGT
TTGGTCCTGTTTGCGCATTTGATCGGAAATATGCAGACCTATTTGCAATCCATCACTGTGAGACTGGAGGAGT
GGAGGGTCAAGCGTCGAGACACTGAGGAATGGATGAAACATCGACAGCTCCCTCAAGATTTACAAGAACG
TGTGAGGAGATTTGTTCAATATAAGTGGCTTGCAACACGAGGAGTCGACGAAGAGTCGATCTTACAAGGTC
TTCCAACAGATCTTCGTCGAGATATTCAGCGTCATCTATGCCTCGACCTTGTTCGACGTGTTCCTTTTTTCGCT
CAGATGGATGATCAACTACTTGACGCGATCTGCGAGCGATTGGCGTCGTCCTTATGTACACAAGGTACTTACA
TTGTTCGTGAGGGAGATCCTGTTACCGAGATGCTCTTTATCATCCGAGGGATGCTTGAGAGTTCAACAACAG
ATGGAGGACGGTCGGGTTTCTTCAATTCGATAACCTTAAGACCGGGAGATTTTTGTGGGGAAGAGCTTCTC
GCTTGGGCATTGCTTCCAAAATCTTCAATCAGCTTGCCATCTTCTACTAGAACAGTTAGAGCAATCACTGAAG
TCGAAGCCTTTGCTCTGCGAGCAGAGGATCTCAAATTCGTCGCTAATCAGTTTCGACGTCTCCATAGCAAGA
AGCTTCAACACACCTTCCGGTTTTACTCTTATCATTGGAGAACCTGGGCAGCTTGCTTCATTCAAGCAGCATG
GCGTCGCTTCAAAAGGAGAATCATAGCAAAGTCGCTCAGTATGCAGGAGTCTTTCTCACTGACTCCTGAGAA
GCCAGCGGCTGAAGAAGCCGAACAAGAAGAGGAAGAACATAACACTCCGCGCTCCAATTACTCTCAAGCA
AAACAAAACCTTGGTGTCACGATATTGGCTTCAAGGTTTGCTGCAAACACACGTCGAGGAGCACAGAAGCT
TAAGGATGTCAACTTATCGAAGCTTCGAAAGCCTGATGAGCCAGACTTTTCAGAAGAACCAGATGATTAA

>*LcCNGC19* Chr13:34786898-34793306 cyclic nucleotide-gated ion channel 1-like

ATGGTAGGACCAGACTCGACATTGTTTAGGATTATTGTTGGTCGAAAACGAAAACTGCATTCTAAGAATGGA
AGACTTGCGATGTTGTCTGTCATTGCAACCTCAGTGGATCCTTTATTCTTTTACATACTGTTTGTTAATGAAGAT
AGAAGGTGCATTGGATTCCACGACAAGTGGAAGACAACAGCTGTAATTCTTCGCTCAGTCATTGACTTTCTC
TACGCGATTCTTACTGCCTATTATTTTCATGATGGTTATCCCTTACTTAACGATGAAACTGACGGCGTTCTCGTG

AGAGATTGTAAATTCCTTCTGTCCTCCTCTCGTGTTGATTTTATTGCACTTGTTCCCCTCCCTCAGGTTGTTGG
AGCATTTTGGTACCTGTCTACCATAGAGCGAAAAACAATGTGCTGGCATGAACGCTGCCAGTATTGTCCTTTG
AACTGTAATTACGTTCATATTGCAAATAATGCCGACGATTTTTGTTCTGTACAAGCTGAAAATGGATCAAAAA
CTTATGACTTTGGAGTATTCAAAGATGTCGTTCCTATTGTGAATTCAAGAGATTTCATTTGGAAAATCTCTTAT
TGCTTTTGGTGGAGTCTGCAGAAATTGAGGTTTTTTTTAGTCATCTCGTTGTCTCTGATAAACTTATACCCTTG
CTATGTGATAATAGTTTTTCTTGCGAAAACCAGTGGACATTTGTTAGAAATCTACTTTGCAATCACCATTACCA
TTTCTGGCTTGGTATTATTTGCACTTCTTATAGGAAATTTGCAGACATATTTGCAGTCAACCATTAGAAGCAAG
GAGAAGATTAGATTGAAGGAGCAAGATATTGAGAAGTGCATGACTAACTATTCTATCCCAGAAAAGTTGAAG
ACGCAAGTTAGAAAATGTAAATTGTACAAGCAGCGAGAAGCCAAAGATGTCGGCGTTGAGCAGCTTCTCCA
AAATCTTCCCAAGCATATAAGTAGGGATAGAAAAGAACACCTTTGCTCTACTTCACTTTTCAAGGTGCTCTTA
AATGTTGATAACATGGATAAGATATTGTCGGATGCAATCTATGATTTGCTAAAGCCAAAGCGATATATTGAACA
AAAGTTCATTGTTCAAGAAGGAGGGGCAGTAGATGAAGTGGCCTTCATAATTCAAGGCAAGGTATTGGTCA
GTTCCAAAAAAGATAGCAAGCCAGAAGAGTTGAACCGGCTTACCAAAGGATCCGAGTAA

>*LcCNGC20* Chr13:42524448-42526976 cyclic nucleotide-gated ion channel 1-like

ATGGTAGGACGAGACTCGACATTGTTTAGAATTATTGTTGGTCGAAAACGAAAACTCCATTCTAAGAATGGA
AGACTTGCGATGTTGTCTGTCATTGCAACCTCAGTGGATCCTTTATTCTTTTACATATTGTTTGTTAATGAAGAT
AGAAGGTGCATTGGATTCCACGACGAGTGGAAGACAGCAGCGGTAATTCTTCGCTCAGTCATTGACTTTCTC
TACGCAATTCTTACTGCCTATTATTTTCATGATGGTTATCCGTTACTTAACGATGAAACTGACGGCGTTCTCGTG
AGAGCTCGTAAATTCCTTCTGTCCTCCTCTCGTGTTGATCTTCTTGCACTTCTTCCCCTCCCTCAGTTGCTGGC
TTTATTAGTCATTCCAAGATCCAAAGGACTCCATTTTATACATGCAATAAGGGCACTGAATTATGTTGTCCTTG
TTCAATATTTGTCAAGGGTCATTCGAATCTACTCTTTCCTAATGAAATCTGGATGGAGCACCATTCTCCCACAA
GCTGCAGAGGCTAGAGCTACATTCAATCTCATCCTCTATATGCTTGCAACTCATGTTGTTGGAGCATTTTGGTA
CCTGTCTACCATAGAGCGAAAAACAATGTGTTGGCATGGACGCTGCCATTATTGTCCTTTGAACTGTAATTAC
GTTCTTATTGCAAATAATGCCGACGATTTTTGTTCTGAACAAGCGGAAAATGGATCAAAAACTTATGACTTTG
GGGTATTCAAAGATGCCCTTCCTATTGTGAATTCAAGAGATTTCATTTGGAAAATCTCTTATTGCTTTTGGTGG
AGTCTGCAGAAATTGAGCTCTTTTGGTCAAGACCTGAAAACCAGTGGACATTTGTTAGAAATCTACTTTGCA
ATCACCATTACCATTTCTGGCTTGGTATTATTTGCACTTCTTATAGGAAATTTGCAGACATATTTGCAGTCAACC
ATTAGAAGCAAGGAGAAGATTAGATTGAAGGAGCAAGATATTGAGAACTGCATGACTAACTATTCTATCCCA
GATAAGTTGAAGACGCAAGTTAGAAAATGTAAATTGTACAAGCAGCGAGAAGCCAAAGGTGTCGGCGTTG
AGCAGCTTCTCCAAAATCTTCCCAACCATATAAGTAGGGATATAAAAGAACACCTTTGCTCTACTTCACTTTTC
AATGTGCTCTTAAAGCTTGATAACATGGACAAGATATTGTCGGATGCAATCTATGATTTGCTAAAGCCAAAGC
GATATATTGAACAAACGTTCATTGTTCAAGAAGGAGGACCAGTAGATGAAGTGGTCTTCATAATTCAAGGCA
AGGTATTGGTCAGTTCCAAAAAAGATAGCAAGCCAGAAGAGTTGAACCGGCTTACCAAAGGTCACTTTTAT
GGAGAGGAACTTGTAGATTAG
