# Supplementary material for: Genome-Wide Identification of the Cyclic Nucleotide-Gated Ion Channel Gene Family and Expression Profiles Under Low-Temperature Stress in Luffa cylindrica L
Source: Int J Mol Sci. 2024 Oct 21;25(20):11330. doi: 10.3390/ijms252011330 (PMC11508470; doi:10.3390/ijms252011330)
Supplement: Supplementary file 1 [file ijms-25-11330-s001.zip › Supplementary File S2.pdf]

>LcCNGC1

MPLFGMLGRLCSTLPYSGLSYLKFSNCFPLFFRSSLKKGVSTKEVFGLYDITIQIV
IMTYLQEKIVRFQDWSSDKTSRGLYSADNTLNTGKNGTRADLVSEKPLKELET
GSYRINRIKKSLKSSSFNKFMSRGFGTGKKVLDPQGPFLQKWNKIFVLSCVIAVS
LDPLFFYVPVIDDEKKCLGLDKKMEITASVLRSFTDIFYILHIVFQFRTGFIAPSSR
VFGRGVLVEDAWAIAKRYLSSYFLIDILAVLPLPQVVILIIIPNMKGSRSLNTKDL
LKFVVFFQYVPRFIRIYPLYKEVTRTSVLTETAWAGAAFNLFLYMLASHVFGAF
WYLFSIERETTCWQRACHNHTGCVSNSLYCDVSPGNNAFLNTSCPTVGGDNPP
FDFGIFLDALNSGVVESMDFPQKFFYCFWWGLRNLSSLGQNLQTSTYVWEICF
AVFISISGLVLFSFLIGNMQTYLQSTTTRLEEMRVRRRDAEQWMSHRLLPESLRE
RIRRYEQYKWQETRGVDEENLVRNLPKDLRRDIKRHLCLSLLMRVPIFEKMDE
QLLDAMCDRLKPVLYTEESYIVREGDPVDEMIFIMRGKLLSVTTNGGRTGFFNS
EH**LKAGDFCGEELLTWALDPHSSSNLPISTRTVRTLSEVEAFAL**KADDLKFV
ASQFRRLHSKQLRHTFRFYSQ**QWRTWAACFIQAAWRRY**RRKKHEQALLEEE
NRLKDALAKTGGNSPSLGATIYASRFAANILRTIRRTSSRKARIPERIPPLLLQKPA
EPDFTSEENS

>LcCNGC2

MHNIAYSRLGGLQKSLSLYRKVPWWDQILEPDSDFVIRWNRIFLVTCLIALFVDP
LYFYVSVIGGPACMRFHIELCIVVTFFRSVVDLFSLFHILMKFRTAFVAPNSRVFG
RGELVKEPRDIAMRYLKKDFVIDLAATLPLPQIVIWFVIPALKNPTATHANHTLA
LIVLIQYAPRLFVIFPLNRQINKTTGAIAKTAWAGAAYNLLLYLLASHVIGSAWY
VASIQRQDECWKLQCRKEMNTTHSPSCSPLFLDCESLNDPERQAWLRVTSVLTN
CDTLNDEKSFEFGMFADAFTDEVASANFFEKYFYCLWWGLKSLSAYGQNLTTS
TYVGEILFSILICSAGLVLFSHLIGQVQSYLQSTTARLEQWRVKRRDTEEWMTH
RQLPLHLQERVRRFVQYKWIATRGVDEESILRSLPLDLRRQIQRHLSLALVRRVP
FFAQMDAQLLDAICERLVSSLNTKDTFITREGDPVNEMLFIIRGQLESSTTNGGR
SGFFNSIT**LRPGDFCGEELLTWALVPSPSLSFPSSTRTVKSLTEVEAFAL**RAED
LKFVASQFKRLHSKKLQHAFRYYSH**QWRTWGSCFIQAAWRRY**VKRKLAMEL
ARQEELFYTTILDQEQSHGSEMEDQEDGQTSGSKPKIANKPKHLGITMLASKFA
ANTRRGIHQKLSALESDATSLKMPKLFKPDEPDFSAFQHGS

>LcCNGC3

MTERWHSTTSKAPSHQHSVARRSMKRVKSQEGSSQKSKIILKVGTGGGAAPFY
RRLEGGSSKVDGWWKSLKFMRKLAALKPILRELNFGVLGDTLFREWEQDFSIL
CSYGQNLETTTFIGETLFAVLIAILGLVLFAHLIGNMQTYLQSLTVRLEEWRLKR
RDTEEWMRHRQLPEDLKRRVRRFVQYKWVATRGVDEEAILQSLPADLRRDIQC
HLCLDLVRRVPFFAQMDDQLLDAMCERLVSSLSTEGTYIVREGDPVTEMLFIIR
GRLESSTTNGGRSGFFNSIM**LRPGDFCGEELLSWALHPKSTTNLPSSTRTVRA**
LNEVEAFALRAEDLKFVANQFRRLHSKKLQHTFRFYSH**HWRTWAACFIQAA**
WRRHKKRMMAKDLLMKESFTLSEEVADETGQGEGEQFSVVSNPSQTKMYLD
VTLLASRFAANTRRGAQRMKDDLPKLQKPDEPDFSIEPDR

>LcCNGC4

MAASRRWRSVTVRFQEEELESGEEESWRVFPEDYDDGMVLDPRMAILNRWNR
VFLVACLVSLFVDPVFFFLPVVKAEEGCVEMGGGLAVALTVVRSAADAFYITQIL
VRFRTAYVAPSSRIFGKGELVIDPSKIAANYLVFHFWLDFAAALPLPQAFIWVAIP

NIRGSNMTPWCYFLRFSILFQYLLRLYLIFPLCDQIINATGVLMKTAWAGAVYNL
MLFMLASHVLGSCWYLLSIGRQMECWKKVCHLGHLDCQYEFFDCKAVGNPN
RAAWFEASNISNLCHPTATTFFHFGIFSDSFASTSSTFFSRYFYCFWWGLRNLSSL
GQNLLTSSNVAEINFAIVIAIVGLVLFALLIGNMQTYLQSTTLRLEEWRVRRTDTE
RWMHHRQLPYELKESVRKYDQFRWIATRGVDEEDILKGLPMDLRRDIKRHLCL
DLVRQVPLFNEMEERMLDAICERLRPCLSTSNTCLVREGDPVNEMLFIIRGHLD
SHTTNGGRTGFFNSSR**LGPTDFCGEELLPWALDPRPAAVLPSSTRTVKAITEV**
EAFALIADDLKFVAAQFRRLHSKQLRYTFRFHSH**QWRTWAACFIQAAWFSY**K
RRKEAAEVKKEIVVGRKYAANCRRVVGRGGSGRRCDGGEMNAVHGSLLKPIE
PDFSVEER

>LcCNGC5

MSMIYERQLQRTVDETKEHRRKAPLAGRQNNNENVQSLRSRCVALVSSLTITIS
SSPRNAVADFFICSHFHFLLLSAALDFASDSSTESRYLEMAAFEKDDIPMLSNTDP
PLLDEEVDSYFPSYASLGRSSSLSIPTTSSGMYGSEANLVGYTGPLRSERKSSFIV
NGSKYTGHKSEKLSQSNPVVTESKTAEQLADKFPSSKTKDEPDWYIHNYAGRN
EHLIRSGQLGVCNDPFCITCPTYNFKALQQKSSRTAGIFDPVFHNDLYGEGKGW
AGKFRSFWCFLLPYIPGVMNPHAKVVQQWNKFFVISCLVAIFLDPLFFFLLAVQ
EENKCIFIDRTMTTTLVVFRSVTDFIYFLHMLLQFRLAYVAPETRVVGAGELVDH
PKKIAMNYLKGNFFIDLLVVLPLPQIIVLLILPKSLGSSGANYAKNLLRTVVLVQ
YIPRLYRFLPLLAGQSPSGFVFETAWANFVINLLTFMLAGHIVGSLWYLFGLQGV
NRCFRRACNDTGTTSCLEYIDCGHGYEDESQNELPLRKAWKENPNATACFAKE
GFDYGIYLQAVNLTTENSIITRYTYSLFWGFQQISTLAGNQVPSYYVYEVLFTM
GIIGLGLLLFALLIGNMQNFLQSLGRRRLEMSLRRRDVEQWMEHRRLPENLRR
QVRQAERYNWASTRGVNEERIFENLPEDLQRNIRRHLFKFVSNVRIFALMDYEP
ILDAIRERLRQKTYIEGSEVFSAGDIIEKMVFIVRGKMESRVDGTGIVVP**LIEGD**
VCGEELLTWCLEHSSINRDMKRPQIPAQRLVSNRTVKCLSNVEAFSLRAAD
LEEVTSMFSRFLRNPRVQGAIRYESP**YWRYLAATRIQVAWRYR**KRRLSRAQTS
QSND

>LcCNGC6

MDPVSVLDCLSVALVPSLSHTHGSASSGLIPLELQPKVSFGKIFMAYGSSRSVRF
QDDLESSTLPTINGGGVKKIIYNIDGSQIPESSGKRTEVSGKSGRSLRAKVLSRVF
SEDYERVQRKILDPRGQAIRRWNKIFLVACLVSLFVDPLFFYLPVVRNEVCIDIG
VGLEVVLTTIRSIADVFYTIQIFIKFRTAYVAPSSRVFGRGELVIDPCKIAIRYLRHG
FWIDFIAAVPVPQVLIWIVIPNLSGSTMTNTKNFLRFFLIFQYLPRLFLIFPLSTQIV
KATGLVTQTAWAGAAYNLILYMLASHVLGACWYLLSIERQEACWRRLCKFDKS
CKDGFFDCHKADDPQRDSWFKTSNITSSCNPNDPFYQFGIYGDAITFDVTTSPFF
NKYFYCLWWGLKNLSSLGQNLATSTFVGEIIFAIIVATLGLVLFALLIGNMQKYL
QSTTVRLEEWRIRRTDTEQWMHHRQLPPELRQSVRRYDQYKWVATRGVDEEA
LLRSLPLDLRRDIKRHLCLDLVRRVPLFDQMDERMLDAICERLKPALSTEGTFLV
REGDPVNEMLFIIRGHLDSYTTNGGRTGFFNSCR**IGPGDFCGEELLTWALDPR**
PSVVLPSSTRTVKAISEVEAFALIAEDLKFVSSQFRRLHSKQLRHKFRFYSH**QW**
RTWAACFVQAAWRRYKRRKERAELRARESYPATKLESPLSPQTASNVDARSG
NARRGMNKRCASDAGVVSSLQKPEEPDFSIVEE

>LcCNGC7

MFDCGYKSQLMGGQREKFVRLDDLDSRLSSPSDSGMRRCGFNIDGFNRAVHG
SDKPSGSFKRGMRKGSEGLKSIGRSLKFGVSRAVFPEDLKESKKKIFDPQDKLL
LFLNKLFVISCILAISVDPLFFYVPVINQNSNCLGIDRKLAITVTTLRTIIDVFYLIH
MALQFRTAYIAPSSRVFGRGELVIDPAQIAKRYLRRYFIIDLVSVLPLPQIVVWRF
LQRSKGSDVYVTKQALLLIVFLQYIPRFLRAFPLASELKRTTGVFAETAWAGAA
YYLLLYMLASHIVGALWYLLAVERNDSCWQKYCIAPSCRKDFLYCGNQDMEG
YATWNRTSVESSCKPADDNQLFDFGIFQQALSSGIAASKDFINKYCYCLWWGLQ
NLSTLGQGLQTSTYPWEVIFSIALAVLGLILFALLIGNMQTYLQSLTIRLEEMRVK
RRDSEQWMHHRLLPQELRERVRRYDQYKWLETRGVDEQSLVQTLPKDLRRDI
KRHLCLALVRRVPLFESMDERLLDAICERLKPCLFTEYTYIVREGDPVDEMLFII
RGRLESVTTDGGRSGFFNRTF**LKEGDFCGEELLTWALDPKSGSNLPSSTRTV
KAITEVEAFAL**VAEELKFVASQFRRLHSRQVQHTFRFYSQ**QWRTWAACFIQA
AWRRY**SKRKSMELRQKEEVAAEESEGSQTATSGGSYSIRATFLASKFAANALRG
VQRYRNAKSAQELIKLQKPPEPDFSADDAD

>LcCNGC8

MPSLPSLPFSSPMWTQLCLRRTSPPLNAHHDLNLTAAAPDDSPITTTVECYACTQ
VGVPAFHSTSCDHAHQQPQWEASAGSSLVPIQPTKASPRRPAPHHPSGRFGTVL
DPRTKRVQRWNRALLLARGMALAVDPLYFYALSIGRGGSPCLYMDGGLAAVVT
VLRTCLDFVHLWHVWLQFRLAYVSKESMVIGCGKLVWDARAIASHYVRSFKG
FWFDAFVILPVPQIVYWLVLPKLIREERIKLIMTVILLMFLFQFLPKVYHSIILMR
RMQKVTGYIFGTIWWGFGLNLIAYFIASHVAGGCWYVLAIQRVASCIQQHCERN
KCNLSLSCAEEVCYQFLSSATTIGNSCGRNSTTTFRKPLCLDVNGPFAYGIYKWA
LPVISSNSIAVKILYPIFWGLMTLSTFGNDLEPTSNWLEVCFSICTVLSGLLLFTLL
IGNIQVLLHAVMARRRKMQLRCRDLEWWMRRRQLPSRLRQRVRHYEHQRWA
AMGGEDEMELINDLPEGLRRDIKRHLCVDLIRKVPLFQNLDELILDNICDRVKPL
VFSKDEKIIREGDPVPRMLFIVCGRIKRSQSLSKGMTATSLI**IEPGGFLGDELLSW
CLRRPFLERLPASSATFVCIEPTEAFAL**KADHLKYITDHFRYKFANERLKRTAR
YYSS**NWRTWAAVNIQFAWRRY**RKRMRRPVIAVVENGSNERRLLQYAAMFMS
FRPHDHLE

>LcCNGC9

MFDCGGYKSQYIGGHKEKFVRYVLEFLNSINIQFLRIHSRDFSFHGPRRLDDLDS
NLSVPSGSSKMKKCRFNLEGLPLPFISRAKPRNASKSFRKGVQMSSDGIMTLGR
SLRSGVSRVIFPEDLKVSDQKIFDPQDKSLLFWNKLFVLCCILAVSVDPLFFYLP
VFNHASYCLGMDTQLAVTTTTLRTAIDVFYLIRMGFQFRTAYVAPSSRVFGRGEL
VIDPADIARRYLQRYFVADFLSVLPLPQVVVWRFLHRSKGSEVLATKQALLNIV
FLQYIPRFIRFIPLNIELKKTAGVFAESAWAGAAYYLLLYMLASHIAGAFWYLLA
VERNDACWRQACKSSGKCNINFLYCGNKHMSGYKEWRNISVDVLTKKCTAIG
DNLPFNYGIYTQAIASGIVQSRTFFSKFCYCLWWGLQNLSTLGQGLLTSTYPGE
VIFSILIAISGLLLFALLIGNMQTYLQSLTVRLEEMRIKRRDSEQWMHHRLLPPDL
REKVRRYDQYKWLETRGVDEESLVQSLPKDLRRDIKRHLCLNLVRRVPLFANM
DERLLDAICERLKPTLYTENTYIVREGDPVDEMLFIIRGRLESVTTDGGRSGFFN
RGV**LKEGDFCGEELLTWALDPKSGANLPSSTRTVHALTEVEAFAL**EAEELK
FVASQFRRLHSRQVQHTFRFYSQ**QWRTWASCFIQAAWRRY**IKRKMAELRRKE
EEEDAAAAAAYSSSRLGATILASRFAANALRGHRMRNVSSGKSLINLQKPSEP

DFSVYKGE

>LcCNGC10

MNRIIHSPASTFRPFRRNLIGSSSDSATTAVAAAAADEHPNYILLRYQILDPDSDIV
AQWNRVFLITCLIALFIDPLYFYTSSVSGPACLTTQMNLAVVITFFRTVTDLFFLL
HMVLKFRTAYVAPSSRVFGRGELVMDARAIATRYLKSDFVIDLAATLPLPQIVM
WLVIPATRHSRADHANNTIALLVLLQYVPRLFLIFPLNQRIVKTTGVVAKTAWAG
AAYNLILYMLASHVLGSTWYLLSIGRQFSCWSSECAKENASKVLTCLPIYLDCA
SLNNTERQYWLNITQVTSKCDPRNKNIKFKFGMFSDAFTNDVASSHFFAKYFY
CLWWGLRNLSSYGQTLDTTTYIGETLFCISTCIFGLILFSQLIGNMQTCLQSMTV
RLEEWRIKRRDTEEWMRRRQLPPDLQERVRRFVQYKWVATRGVNEESILRSLPI
DLRREIQQHLCLSLVRRVPFFSQMDDQLLDAICERLVSSLCTQGTYIVREDDPVN
EMLFIIRGQLESSTTNGGRSGFFNSIT**LKPGDFCGEELLTWALMPSSNLNMPSS**
TRTVRALTEVEAFALRAEDLKFVAGQFKRLHSKKLQHAFRYYSH**QWRTWGA**
CLIQVAWRRLQKRKLAKKLALRESLCYIDSIEQDNEYDIELAEEDYDDDSDED
MSSTDYMNKTQNLGATLLASKFAANTRRGINQKGQTSKPSSLKMPKLFKPDEP
DFSMDG

>LcCNGC11

MAKCMGSWKPKSERFPYPPGFFVKYEAKQKKSRLSPILESISIAWKKTLDPQKP
FLQQWNKIFVLSCVIAVAVDPLFFYIPVFDGQHQCLSMDQPLTIVACVLRSFIDIF
YLLHMIFEFRTGFLPSGSHTSALLGSADLIENLAAIAKRYLFSNFTIDILSILPVPQ
LLILVIVPALKGTIPLKTKNFVKIAVLLQYIPRLFRIYPLYTEATKTSGILTERAWSG
AAFNLLIYMLASHVVGALWYLFSVEQQARCWLEACKKNNCTSKFLYCGDHSR
QAYPFIDEYCPHKETEDDNVFNFGMYVEALKFHLTETTSFRRKFIYSFWWALRN
VGSSGQNLQVSNHMGEVFFAVFIAILGLVLFAFLISNIQKYLQSATVKIEQMRINR
RDAEHWMAHRMLPEDLRQRIRRYDQYKWQLNRGVKEEELISNLPKDLRRDIK
RHLCLALLKKVPLFSSMNKQLLDSVCEYLKPVLFTEKSFILQEGDQIDVMLFIM
KGNLAAITTNGWKNDPWSVT**LKAGDFCGEELVQWAMDPTSTSLPISTRKVK**
SLTEVEAFALKANELKSVTSQFHFQRLNSKQFQLSVRFYSH**QWKVWAAYKIQ**
EAWHDYRERKKRGGGNGRFQDALAETVGASASFGATLYASIFISHLLQAVQRD
QHHQTTQLTRVMTLPPPPKPDDEQDRPNFTILNL

>LcCNGC12

MKKELAMNAEEDDSIRIQSCSREREIEKLSSHGSNCSVKSKGTVGSVPKRFLSST
DKLTSFGNIQFDEEVNCKVLSSIKAYIEGNMIILHLWNDLLVILCVMATILDPLFC
YTLVVNEERNCIGFDKKLRVIVVVLRSLIDFGYMIMIIFHFHIGYTASHDAKSRR
LCSTARRYLLSYSTVDILTLLPIPQVMVLLVIPGSKGSHFTAAIKSMKFVFIIQYLP
RVFRVQSFLKKVRWSSGIFLNTTGIKAIFNLFLYVLASHVFGAFWYLFSVERRAT
CLQVRCHSHPYCPKMYNDNSVESFCTDACSAKALSNDTAAFNFGIFDDAFKSG
VVYTPDFIWKISYCYWWGLQNLSSLGQGLKTSKYIWEIYFASTLTMSGLVLFAF
LVGNLQTILQATFARVEELRSKGHDIEMWMAYHSLPRNLKKRIKRFEKYRWHK
TRGVDVENILQNLPRDLRRDTTRHLCLGVLASVSMFQNMDEKFLDAVFGFLKP
MLYIEHNFIVREGEPLDEMIFIVQGKLWIYSKSSKDDETSCSWPGTQS**LQKGDF**
FGEELLNWVLQDPFLSTVPISTKTVAAHTKVEAFVLSANDLQIVVSKFWWL
FSREFRNDPLFKE**RWAPWAALVLQAAWRRY**FKNKREKEKEKSQLGLVTKSGN
QPSVTTAVHVAKFVVGALHALNRRRKGKERSNKLLESSKGNGLPEPSNV

>LcCNGC13
MNGEDDAIRIQSCSTEHKTEGLSSHGEIRSANSMGTVGPVPRRFESSSNILTSFG
NIHFDEEVKSKGLSSIKKSIEGNLLFLHLWNDVLVMFCVIATLLDPLFCYILVVEE
EKNCIGFDKKLRITAVVLRSLIDFGYILLIVFHFRIGYTAPNDANDGRLLTIVGRY
LFSYFTIDILAVLPLPQVVILLVIQATKGSHFTVAIRSLKFVLIIQYLPRVFRVYSFL
KKVRWSSGILPDSAGAKAIFNLFLYMLASHVFGAFWYLFSIERKATCLQVRCHS
HPYCPRNYNTSAERLCIDNCSGKASSNDTPAFNFGIFEDAFNYGVVSSTDFIWK
FSYCYWWGLQNLSSLGQGLKTSKHIWEIYFAVSITIAGLVLFALLIGNLQTFLQA
TIARLEEMRLKGQDIELWMAYHSLPRDLRKRIKQYEKYKWRKTRGVDVANILH
NLPRDLRRDTTRHLCLRAIKSVSMFQNTDEKFLDAVCGYLKPMLYIERNFIVRE
GEPLDEMIFIIHGKLWIYSNSNRGGETSGSSES**LQKGDFFGEDLLKWVLKDPV**
LSTVPISTKTVSTHTKVEAFVLSANDLKNVVSKFWWLFSRELRNDPNFKE**RW**
APWAALVLQAAWRRYFRSKRERERSQLSLATESGNSQPLVTTTIHASRFIARAL
HALNQRRKTRNGSNDMPGPSNSKDLPKHSNV
>LcCNGC14
MEVQKSPDGLYQKRLSSAGETHSRDSLRSLRSVSTNFAASFDRLTSFGNIYFEQE
VRSKGLGYLKSIVDKNPLFLHLWNEILVMLCVIATSLDPLFCYILLVDEGKRCVG
LDKKLRTVAVIIRSIIDFLYIILIFFHFQFGYSSFYNANRDNGDNSDDGVCTRAWR
FLLSYFTVDVLAVLPLPQVVVLILIPSLKGHDFIYAVRSLKYILLVQYLPRVFRIYS
FLKKVRWTSNILPETAGAKAAFNLFLYMLASHVFGAFWYLFAIERKTTCWEGS
CQHCPLNCNYVLGNFSADSDSFCSAKAENGSKAFDFGIFKDAFRVAESRDFTRK
FSYCYWWGLQKLSSLGQDLKTSDHLWEIYFAVTVTISGLILFALLVGNLQTYLQ
STIARLEEMRLKGQDIELWMAYHSLPSNLKKKIKKYERYKWRETKGVDVELLL
HNLPRDLRRDTKRHLCSTPLKKVSNLQNMDEKLRDAICDYLKPVLYIERNYIV
KEGEPLDEMVFIIRGKVMVYSKRDGEAAAGSSGSKW**LSKDDFYGEDLLEWA**
LRNPTSTTVPISTKTIRAHSKVEAFVLMANDLKTVVSKFWWLFSRNSPSLKA**I**
WAPWAALALQLAWRRYHKSKNEKNKSELAIERRNTQANLTAPLLTTTHIARA
LRAFKLNGKKAQAESSRV.
>LcCNGC15
MNSALLRGLASSSDGTRNTEKLLKSSSSNNRMPEGLHSGKKILDPQGPFLQSW
NKMFVLSCVIAVSLDPLFFYVPVIDNRRKCLRLDEKVETVVCILRLFTDLFYVV
HIVFQFRTGFIAPSSRVFGRGVLVEDYRAIAMRYLSSYFLIDISSVLPLPQVVILIIV
PVMGSSRPSITKDLLKFAVLCQLVPRFLRIYPLYKEVTRTSGILIETAWAGAAFNL
LLYMLAGHIFGATWYLCSIEREAQCWHDACSKHPGCNSTSLHCDYNSSVGGNL
FLNVSCPIEKPNVGHFNFGIFLQALRSDIVESDFPKKFLHCFWWGLRNLSSLGQ
NLTTSTCTWENCFAILVCISGLVLFASLLGNVQMYWRSTNTRVEEMRVRRRDVE
QWMSHRLLPENMRERVRRYKHYTWLETRGVDEHNLLLNLPRDLQRDIKRHLC
LPLLMRVPMFEKMDEQLLDAMCARLKPVLYTQESCVVREGDPVDEMLFIMRG
KLLTMTTNGGRTGFFNSDF**LMAGDFCGEELLTWALDPHSSTNLPISTRTVRS**
LTEVEAFAFKPDDLKVVASQYRRLHSKQLRQIFRFYSH**QWRTWAACFVQAA**
WRRHRRKQLRESLREEESRLKDALACLEGRSPSLGATIYASRFASNMLRAMRR
NGTRKARMSMLLQKPAEPDFTLEDNNT
>LcCNGC16
MMPTKFNTTHAELDEFQDDDHHQNGSFAGSSRRRVLDPRCACVQRWNRVVLL

ARAASVAVDPLFFYVIFLTKAGPPCFYRDAAFAAVVTAVRTCVDMVHVCHVWM
QFRLAFVSTASLVVGSGRLVWDARAIASHYLRSFKGFWLDLFVIIPIPQVVTWLI
VPKLLREEEIKKMMKVVLVSYLFQLLPKLYHSIYLMKKLQKVTGFIFGCIWWR
FNLNVFAYLIASHVAGGCWYLLATQRLISCLEQQCERRKMCKLALSCSSNSPTS
GYYKGAGKFGAPHGNNLTKIKSLCLEVNGPFSYGIYEPVLLVFSSNSLAVRILYP
VFWGLLNLSSFGNELDPTSNLVEVIFSSCITLAGLVLFVTLIGNIQIFLQTVMASE
ENMQIRFRDMKWWMRRRQLPIHLRERVCNFEYQRWVAMGGLDETELIKGLPD
GLRRDIKRYLCLDLVKKVPFFHMLDDLILDNICDRVKPLLYAEGEKIVREGDPV
QRMVFIVEGCVERSQGFVGTSL**LNSGGFFGEELLSWCLRLRHRRHFDELPTS**
LATFSCVQPVEAYGLDALDLKYITEHFRYKFASEKIKRTMRYYSS**NWRTWGA**
VIIQSSWRRHRIRTRGYTTRDRTADNRLRQYAAVFLSMKPNDHLD

>LcCNGC17

MATTPSHSHHHTATTSDEEEEEKDLEDDQSNGAAFCRNLYGVGSVLDPRTKWV
REWNRVFLLVCAGGLFVDPLFLYTLSISESWMCVFVDGWLAITVTVLRCMADA
LHLWNIWLQLKTATRSSFAAADNTRLTDTTPRAVALRYFKSKKGFFFDLFVILPL
PQVLLWVVIPWIMKEGSVTLVMTVLLIVFLFQYLPKLYHSVCLLRRLQNLSGYI
FGTVWWGIALNLIAYFVAAHAAGACWYLLGIQRAAKCLKEQCRATSSCGLRSL
SCKDPIFYGATDMNMGRDRARFDWANNRLPKFMCLDTADNFDYGAYKWTVQ
LVVNQSRLEKILFPIFWGLMTLSTFGNLESTTEWLEVVFNIIVLTSGLLLVTMLIG
NIKVFLHATTSKKQGMQLKMRNLEWWMRKRRLPQGFRQRVRNYERQRWAA
MRGVDECEMIRNLPEGLRRDIKYHLCLDLVRQVPLFQHMDELVLENICDRVKS
LIFTKGETITREGDPVQRMLFVVRGHLQSSQVLRDGVKSCCM**LGPGNFSGDEL**
LSWCLRRPFIERLPPSSCTLVTLETTEAFGLEADDVKYVTQHFRYTFVNDKV
KRSARYYSP**GWRTWAAVAIQLAWRRY**RHRLTLTSLSFIRPRRPLSRCSSLGEDR
LRLYTALLTSPKPNQDHFDF

>LcCNGC18

MIWFIMPAIRSSHADHTNNTLVLVVLLQYIPRFYLIFPLSSHIIKTTGVVTKTAWA
GAAYNLVLYMLASHILGAAWYLLSVERHAMCWKFTCRREFSPMKCLLDYLDC
GTLDYVDRRIWEVNTTVFSQCSPDEDVVFNYGIFADAITKNVISSGFVQKYFYC
LWWGLQNLSSYGQGLETTTFIGETLFAILIAIMGLVLFAHLIGNMQTYLQSITVR
LEEWRVKRRDTEEWMKHRQLPQDLQERVRRFVQYKWLATRGVDEESILQGLP
TDLRRDIQRHLCLDLVRRVPFFAQMDDQLLDAICERLASSLCTQGTYIVREGDP
VTEMLFIIRGMLESSTTDGGRSGFFNSIT**LRPGDFCGEELLAWALLPKSSISLPS**
STRTVRAITEVEAFALRAEDLKFVANQFRRLHSKKLQHTFRFYSY**HWRTWAA**
CFIQAAWRRFKRRIIAKSLSMQESFSLTPEKPAAEEAEQEEEEHNTPRSNYSQA
KQNLGVTILASRFAANTRRGAQKLKDVNLSKLRKPDEPDFSEEPDD

>LcCNGC19

MVGPDSTLFRIIVGRKRKLHSKNGRLAMLSVIATSVDPLFFYILFVNEDRRCIGF
HDKWKTTAVILRSVIDFLYAILTAYYFHDGYPLLNDETDGVLVRDCKFLLSSSRV
DFIALVPLPQVVGAFWYLSTIERKTMCWHERCQYCPLNCNYVHIANNADDFCS
VQAENGSKTYDFGVFKDVVPIVNSRDFIWKISYCFWWSLQKLRFFLVISLSLINL
YPCYVIIVFLAKTSGHLLEIYFAITITISGLVLFALLIGNLQTYLQSTIRSKEKIRLK
EQDIEKCMTNYSIPEKLKTQVRKCKLYKQREAKDVGVEQLLQNLPKHISRDRK
EHLCSTSLFKVLLNVDNMDKILSDAIYDLLKPKRYIEQKFIVQEGGAVDEVAFII

QGKVLVSSKKDSKPEELNR**LTKGSE**

>LcCNGC20

MVGRDSTLFRIIVGRKRKLHSKNGRLAMLSVIATSVDPLFFYILFVNEDRRCIGF
HDEWKTAAVILRSVIDFLYAILTAYYFHDGYPLLNDETDGVLVRARKFLLSSSRV
DLLALLPLPQLLALLVIPRSKGLHFIHAIRALNYVVLVQYLSRVIRIYSFLMKSG
WSTILPQAAEARATFNLILYMLATHVVGAFWYLSTIERKTMCWHGRCHYCPLN
CNYVLIANNADDFCSEQAENGSKTYDFGVFKDALPIVNSRDFIWKISYCFWWS
LQKLSSFGQDLKTSGHLLEIYFAITITISGLVLFALLIGNLQTYLQSTIRSKEKIRLK
EQDIENCMTNYSIPDKLKTQVRKCKLYKQREAKGVGVEQLLQNLPNHISRDIKE
HLCSTSLFNVLLKLDNMDKILSDAIYDLLKPKRYIEQTFIVQEGGPVDEVVFIIQ
GKVLVSSKKDSKPEELNR**LTKGHFYGEELVD**
