# Supplementary material for: Genome-Wide Identification of the Cyclic Nucleotide-Gated Ion Channel Gene Family and Expression Profiles Under Low-Temperature Stress in Luffa cylindrica L
Source: Int J Mol Sci. 2024 Oct 21;25(20):11330. doi: 10.3390/ijms252011330 (PMC11508470; doi:10.3390/ijms252011330)
Supplement: Supplementary file 1 [file ijms-25-11330-s001.zip › Supplementary File S3.pdf]

Table S1 Duplicated *LcCNGC* genes in *L. cylindrica*.

Duplicate gene pair	*Ka*	*Ks*	*Ka*/*Ks*
LcCNGC12/*LcCNGC13*	0.4179	0.3400	0.4349
