# Supplementary material for: Genome-Wide Identification of the Cyclic Nucleotide-Gated Ion Channel Gene Family and Expression Profiles Under Low-Temperature Stress in Luffa cylindrica L
Source: Int J Mol Sci. 2024 Oct 21;25(20):11330. doi: 10.3390/ijms252011330 (PMC11508470; doi:10.3390/ijms252011330)
Supplement: Supplementary file 1 [file ijms-25-11330-s001.zip › Supplementary File S4.pdf]

>LcCNGC1 + Up_Stream_Len 2000
aaataaaaaataaaaaaacttttttttcttttatcaaaaaatggtccctcgttattatta
ttatttagtacaaaggagcagaaaaaaacagaaaaaacgaatggatcacccattagtgag
aatatactttaaaaaaaaaaaaaaaaaaaagttcaatccttcatgggacgaggcaatgat
ggaatgacttttctgtccttgtccctacccctgcgaaaaatattatctatttagtttatt
tattaaaaatattcaatccaatggttaactttccatcaaaaaaacttcatttaagtggat
aattttgcaagaaaatttatatttaaagacaaatttcttaaactttcaaagagaaataaa
ataatattacatatcaataatgtaattaaatggagaacaaggtgaagattgagaatatat
tatttattcctatctaagaaaattttcctccatttcgtacctaaacgacggaggaagaaa
gatgaacattcctacttctacatttgttcattttgatatttatacttttgccttttgtcc
gttttgacaattttgccttcacaacatccacgttgataccttagagggaatgaattcaat
ccatgatgaccacctacctaagatttaatatcttacgtgtttcctaacacccaaatgcag
acggttgtccatgagattagttcagaggaatgttttggacaatttattttttcatatctt
tctcattacaattttcaacaatttcttgaaatactttcttagttttatattaaaaaaatt
tctttatgaaaaaataaattgtatcgtaggatttctattgtatggtacttgtgtcagtag
aattatgcaaacagataccaaaatagatgatttaaaattatattgacaaaaatggacaaa
aattgaaagtacaaaaattaaaatggacaggaaaatagaaaaacgtacacaacccaaaat
aggataaaaaaaaaccttcaatttttttttttttttttgagaaaaacaaaaaaaattattt
aaatatctttacaagtacgtatatataattaaatatagatataatgtaatatatatatat
acatacataatttttgtagtaaaaagaaaattggaaaccgttttttgaaatttttttaaa
taatatagtattttcaaaatttcggttttttttttttttttttgggtctttgtcgtcgtc
gccgtcagtgttggagagacccaactgcaccaatttggactcatctctgatctttcttcc
catatggaaaaataattaaaattaacccaaaaaaaaaaaaaaaaaattggttcttttacg
tgaattatttataaatgtgaacaaaaatccaagatgaagaagtctcgaggaagtttcgta
gtataaagtggaagaagtcgcatttctattgctggattcaatcaatttctttctgccaaa
aacttttttgtttatttgatttaatacccaacaatcaatcatcccagtttcttcttcctt
ccccccctcctcattttggtatggtttcttctccttttccctcaaaggcccgtgtttttt
ttttttccttcagttacttttgattttacagttctagttcgtgggtctcttcgctttcat
tttgggtgtttgagttctggtttttgtgttcttcctgcttggttttactgaatttggtgt
ttatttcagttgtcttgtttgtttctctctgaacttgctttgagatatgagaagtgaaat
tggatgtcgaagtttctttgtttcgattgtattttccccttttaagttggttttcagggc
ttttatttgcataaactggactgctgtggataatcggttgagaacctttggatgtttggg
gggtatttaaagtatttggacatttggatgaatgagtatcttgtttgattttttcaaaac
cccatttgatttttgatcagttttatggtcaattgagcaatttagattctggttttgtca
tttgggatctattgtcagtc
>LcCNGC2 - Up_Stream_Len 2000
gaataatataggttaattataatgatagtaacttagactcgtttttttttttagttcaac
aacatttgagattgagggatttgaacttctgatttcttagttgaagatatacatgtcaat
cattattgagctatatatgcccactttggtaatttaggcttgtttgtaattatactttta
atttttgtttgtatgatatgtttatattaattatgggaagaagaagaaaagatatacaat
aaatagagaatattaaaaccacttaaaaacctagggaggaaccattaagaaacaaaatta
aatttatatgcaaaggatctatgggtttggccttattattgattttggaaatcccaagtt
actttaattaaaatatgtataattttatgggacaagtaggtttaaagttagcttaatttc
tttatatctaatacaatttaaacccttcattttgcttttgaatcagttgttactttttca

ttaatttaatatatatatatatatatatatatatttaaaataatcattaatttagtttaat
atattttatgtttcaaaagaaaaattgatgtttgaattgagaatcttctaaagaaaaatt
agtttaccctaatatattaaaattaagatcgaactaaataaaaaaattgtcaatttgggt
atgtggttgaaatggctagacatcattttcaaaaaatttataaattttacttcattacaa
atttattatctgaattcgaatggtctaaattaaatgatgaacttagtgaaagtcaattcc
aataagccatttttttaatgggtttgtttgaatggttatcatttttttagtttattgaaatc
ttgtaaaagaataatcaatataattacaaaatttgtttactaatattcatatattaaatt
ttaatattggagttttgtatctgtttttatgggtgtgtttgtttgcaatattttattttt
taaagttaaattacaaatttagtcctgaacttttaaggttatgtcaatttggtcattgaa
ctttaaaaagtgtcaaataagtcctcaaatgttcaattttgtgttcaataggttcttaaa
ctttaaaaagtatctgataaaactttaaacattcatttttttgtccaataagtccttcaa
ctttcaattttttttttttctgtatctaattagactttgacccattcgatagtttttaaaa
ttcacaagcctactagttacagggtcccaatagacataaaatccaattttatatttgata
gattggttaatttttaaaaaatcttgaatatgacaaagacgtattagacacgaacattca
agaatctattagatacaaaattgaagctcaattttgtgttcaataggtttctatacttta
aaaagtatatgatagatccttaaactttcacctttttttttttagttcaaactttcactt
tttttgtccaataattcattcaaatttcaattttttttttttaatctaattaggctttgac
ctattcgatagtttttaaaattcataagcctactagatacatggtctcaataaacataaa
attcaattttatattccataaaaattggttaatttttttaaaaatttcaaatacgacagag
acctattagacacacattgaaaattgaaggatctattagacacaaaattgaaggttgaaa
tatttattagacactttctaaagttaaagtggagttccaatttaaggcaaacaataagga
aaatttcccaccaaccttaaagaaatgtagactaaaattcaaatgttaggtaaagtagtt
aaattttggcgcccaaatcccaatattaatatccatagaatcaataaacggccattttcg
gaagcctgtagagagagaaagggctaaaaataatcttctggctccccgaatttctctgtt
cttcttcgagaaaagaaaaaaaaatccttgaattctctctgcatgatcatgatcatgttc
ttcgtctttgcaatggaaaa
>LcCNGC3 - Up_Stream_Len 2000
cctaactcaattgggattaaatcataagagaaaaaactaaatggggaactggatccctat
gaattctccgcaggaaatctttgtctcgattcccgcaaaatttaacaagaaattttggag
ggatgggaatttaaatgaggggtagagacagggacagaaaagccattcttgtctcctccc
cataaacatccctaaccttaactaattgaactttacttaggttgatagaattaacttctt
ctcggtagatactaagaaccacaaccctcgtagtaacattggcaaattaaagtcgacatc
tagaggggtccaaatctccttatttccacttgatctcatccaaaaacatattttcatcat
cttgttttattttattatttactttttagagatttatcatcttagtaggtgatctaacat
ataagtacgtaggaaacaaaagggtcaaaataaaggcccagagggctcaggactggtggg
cctcttaacaaacgagttgggtggctaccctagaaaaagtgaatggcccaaactaaccat
tcagcccatcctttaaggaaagtcgtctagcttaacagttttttttttttttttaataa
ttttttaaatacttttaattgtaattaaaattattattattattattattttttttttttg
ttcacccaaatttgtccttttagtcgagtttcatcgaactattatcaagaagttttaaat
agattgtttttttattcatatataatctatacgtacatttatttaatttcactaatgact
ctttgctcaatgatgtttacaaatgacaattcctctcccaagatttggggtagatgctat
cataagctctaagttagtctcaccagtaatttattaaacatctggttgggggggaaataac
attatcacatggcggcttgaatttatttatttatttttttttttttttttgaaaggcatgg
cggcttgaattttcccataaaatataactaataattaataacttctcacggcaaagcaag

agaacagcaaattatgccatatataatataaatataaaatagcaagtgtctgaaaaggca
cgcaattagaattggattgttcattcaattttcctcacgagtctcctattattaagaaaa
tcagattctttcaaatttacaaccatcaaaaactcttacaacttacaagcattactatta
tacatatttttaaaaaatgaaaataaactatttaaaaaatattcctagtaaataagcatt
actatggttttaagctttttaaaaaagaaaaaaaaaatgaagggagactaatggtctgga
ctggaggtggaggggacgtgtttgggagatccgcaatacaggaaaaagtgaaaaagaaac
acagaaattcagcagaaaagagagattaaaaaaaatgaaaaagaaacagtggatgtccac
ttgaattttaaagtgctattttggatatcactttacaagatacaaaataacttcaacagc
tacattattaatattaatccaaaaagaaatgatactgcaagatctgaacataacatttcc
tattcatcgaacatgtcaaatcattattgcagccaaaaatttaagtagatagattagata
aaatttaatactttaatacattctcaatatatattaatctaaatttatttgttaaataga
aacgattgttcttattgtatgggccatcaaattaaaataaggagggaagaccgtagaata
tgagataatgatttgacaattgcagaatgtttgtttggtgacaacaggctgattgaggag
aacccacatgacaaagataagaaaattcatctttttcttaaaataattatgatatttcct
taaaactttatcaaagcgagctttaagcaggaaagcagccaacttcacactcaaagttga
atgaatccatttccattttggatgaatttttatcaccaaaatcaaaatgagtagtaaagt
aatgttttgctttttagaaa
>LcCNGC4   -   Up_Stream_Len 2000
agactttaatgacaattgaaattattgttgataaaccattttatcaataacagtagtcta
tcgctgatggccggaatttttctttttttccttccaaaaaaaaaaacccaagatacttttttt
ttgacaatatatatgtggggtgggagattcaaaccacagatctcgtggtcatcaatacaa
ctttatgtcagttgagctatgctcttgttggcaaaacccaagatactttgacttaccttt
attagtttaaaaatatgagtgcaatatgtcatgatctcacttctttgaaattgtttaaca
cttgctctcatctttgagcaagtcaactcaaatttgttggtgccacaacgaacctatctc
gcaactcccagttcacctttacttttgaaaacaaagttagagaaaaatgttttgtaaatc
gtttgaaaaaacatggttatagattagaaagtaaataaaataaacaatgtatttgatagt
atatattacaaccaatgtagaaaggatgacaactagtcttaccttgcgcaatacattttg
aagaagatttcttttgtcaatagtaaacatacaaaatgacaaggaatacatcgatcattc
acttacataaatctaaggaaacaaaactagtatgtaaaacgaaagacatatacaatgaaa
ataggatgaaaaaaaggttgttatacttgggtatgcaaccttggacaacatgttctggac
aagtatgtaatataaacaaactaatatatatatatatatatatatatatataaaagaatc
caagttttatcttctttaaattatccaatcccgttatattttaatattaagggtattgct
ttagaacgtagtgatattttcactcctccattttcatccctttaaatgcaaagtaattcg
tgaatgtcaaattccaatgatgggttttagtgagtgggcaattttattccaactccaata
aaacttcatatgtccttttgcccattattaaatttggccaaattgagaacaattgatttt
ttccccctaactatcctttaaaagggagtgggtttaagagtagccaaatttcattggatg
ttcaaataaatctagacaaagtgataattaatacatcattgaaaattttgaactacaaca
ctcattaggtcacaatacataacatctacattacaacatgctaaaaacttgaacattgtt
tttgaaaaagagtattcactactacaaacgttaagaaaaatgtaaaagaaaaaaaacatt
ataataagttataatactaagaaccaacctgaacataactcaactgattaaagtatatgc
cctcaactaaaaggtcagaggtttaaatccttcccatcaagtttgagtgaattgtttttt
ttacgacaagttttactagatgtacggggaagcgtcaagttcgatacaacatttcaaaaa
cgtcaactaagacccttttttttttttataatgacttaaaattcttagcatgttatagtg
ttggaattcacttatgttttgaaaggtcttctctttctccagcttaccttcacacctcaa

aaaccagctgtgcttccccaaaacccaactgctttttctctaacacatgaaatatcaaaa
aacatgtgaagtgaagaagtacttgtgttcaaaacacttcaaagccaaaccccagctgcc
catttatttgatttcacaaggttttcacctaagacaaagagaaagagagagaaattcggc
ttttagagtcacaaaagttggaaaaacacgttttgatcttcacatgatactcacaaaaat
cagtacggaaacaaaaattaaagagcctcagttcatatatggtccagtttcttcaaccca
tgaaccttaaaacctcgccaacaatggacgggtccaaacgagccaagtctcaccaatcga
ataaactcctcatatcactatatagccatttcatttccagtacttctctgtaaaaaaaat
tctcaacaagtcccacagcc
>LcCNGC5 - Up_Stream_Len 2000
agaaccgagatcatttctgcccgttgaacatccctacgtgcaacagatggagtttgtgtt
tgtccatcgagaaatgaatgctcgatcggcttgcaaaacatttcttcatctgcccgctca
gagcattgggctcatgacttttctccactgcttcatttcgctgcctaaaatgttttctca
atctcatcctcggtttttctctttgagatttcattatttaccccaaataattttttaaat
taacttttttccatataaaataaagactaattaatgtaggtaaacttaaacatatagtta
ttataatttaagatatatatactatcaactcagtgatcataagtttaaatctctcgaccc
tcatatgatgtgaactaaaaggagtagtagtgtagaaatgtgtaaccattgtcttttttt
tttttttttttaacgacacgatctaaattagcatctagtgacaattttttttctttcgaa
aagcaccattatttattgaaaactttatgtatgcaatgatggatcctaaatgttattgtt
agatgatctatcattagagaggaggctcgactttaattaaataatagttttaattgcgtc
aatgcaaaatatatatttatgtatacacataacattccaattaaatgaatagcaattatt
aaactttagcccaccaattataccaacatgagcatagctaagctaattgacatctatgct
cattttgggtatgaagttcatgcttccatatttgttgtaataaaaagtaccaattgatta
tatgtattgtaatgagtagcaatttaaggtttgtcgtttgcaaatacaataacttaaaaa
aaaaaaaaaaaagttccaaatatgacaaatctatcaatgatagacttcaatcacaatgca
tgtaatccgtcagcaataattgcaaccatgattaaagtttattgttggtaaatttctatc
acggttagaatttgatattattatatatgtagataattttggaaaatatataagtgatag
atagtgtgatagacatctatcattgataaacttcaatcacaatgcatgtaatctatgagc
aataattacatttgtgattgaagtccatcgctgacagacttctatcactaatagacttaa
actttgctatatttgcaaatagttttagatgttgttatatttgtaattattttagtctat
tttactatttgtgcaacaaacccttgagtatatatataacacatacatacattaacttaa
agaatgcctcattaataggaactagacgtggggctacatctagctgtgattagatgatgg
agttaacttgaaatatttgatggctaacatgagcataactcaactaatataaagttgtac
taatgaccaaaaggtttgtgtttgaatccccactaatgaccaaaaggtttgtgtttgaat
ccccacccttcacatgcagtaaaaaaaaggaatatttaatgggtttttttttataaatta
tatatatatttaacataaaaaagaaaaataaattcatagaccaagtcaagaattttggat
tttcgagataggacaaataacagagtaaaaataattacaaactccaaaatcacttaaaat
ttgttaaagtattttaagttaataatttagctattgcttatgaacaacagctcttttttt
ttttctttcaaagttcactgtaaattatatgagttagtccttttcatgtaggatattctt
ttattaatgaatatcctgtttcaaaaaaaaaaaaaaaaaaaaagaaaaggaaaaagaaaa
aaaaaagaaaaaaaaggaaaaggatggggataaagtattagatgaagcggacaaaacaga
gtccaaaaccaggcatgtgggcgtagggaagcggaataaacaaagtaacaactttcattt
aataaacattattttgaaaggttggtttaattttggttgggtaaagaagaagaagtaatg
ctatttttgcaaatgggcgt
>LcCNGC6 - Up_Stream_Len 2000

aaattattgtatagttaaaactcgttattaaaccattctttttagaaataagtagcaaaa
acttttatcaaatatgttttctttttttttttatttttatttttcaacatcagaaggg
tgtagttaacaagcatgtcaggcttcaaaatttaaatttcagaaaaaaaagaaaaaagaa
aaaagaaaaaggagagagaatttgaattttgtttctaaaaaatagaaaacaaaaaatcaa
aaacaggaaaaaataaaaatgagtacttattgtaagttgtattgatagatagatgacatg
acactaaactacaaaaattcattgtttcgaaaataagtcaccaacttgtacgcgcacaca
atatctcgaaatttatttttatcatctaaagaaatatatatgagaataaataaatgaagg
tttatgcaaatattatatatatatcaaatcacattggtactcaattaatttcatttttgt
tctccttttatttctatcttcattcacccaaatgtttaatttcaatctatatatttttaa
tccatgacctccaacatgcccaaactcccgaagccagtatacgcagttctagattaattt
aatcccaaatgaaatactttaaattaccatacacaacactaaaaataacataaatagtga
aacaaaagtatcaaaactcaaaactctaatctaacatattcaatcaaactttcaagaaga
taacaattctcagcagtcttaatgtactgaacattttaaaatttttgtgggtacgaaaca
tgaatcatgatcggtgcctgagttccaatctctccgcacttgaaccacgatccaatgtta
cacccaacagtcaagaaatagtaacaatccattatatacaaatcttcagaaaaaaaacat
gtttaatacaacatattttaatgaagatcatagagcatcaagaaacatatataaaaagaa
aaatgcatgaatgcaagaaaagacaagctaagtggcctgattcaaaattattcaatggag
ctatatggttgtctattatagacgttggatgtagatttaaaatccatatgccggtcctta
ataactacgggatacagatacaaagtacttttataaatttttctctcatacataaccaac
aatattaattgttatgggtgaatttggacctaattctaagaaggtcagaatgataagaat
caaacaagtccaaggtgggtctgaatcaatctctagggttattctctctcctaaaggaa
gggaaaaccctaattcgactataaatagaagaagctaaacccaagctatggggactcaac
ttggtatttattatcttcctcttcattgttaatttacttgcttcacttatgatctcccaa
ctgacttaagcatcggagtcggtgtagcaagcactacaccggtgcacagtttttttcttgt
tttgctagtcacgtctttccctttcaaacaaatttactgttgtcacgtgaaggtcagatg
agttttctctatccaaattttggtatcaacaagtataaagttttcagccacattgtaact
ggtttcttcaaataatttctcgcatgcgtggagaacgaaagacgtagacggagagagaga
gagagttaaaaaaggacccattgccaaatcagaacagtctgtaaggcaatgcaattagag
gagaaacaggaagaaatgggtgttataaatgcttgcataaggggtgtctgagattatggg
tccatttcttgtccctcttttgtactttgccatttctcactccacaatcttcatttgctt
acttcaaccatttctttattttgtgttttaattaaaaagtcatatgatatctaggatcta
gcactcacctagcttttcgactttcgatggagtttaggagacagtaacttattaaattaa
tggaggtgaatattatacggaaaatgtacgtttttttaataaaaaaaaaattaaaatgca
attggattgaatttggttaa
>LcCNGC7   +   Up_Stream_Len 2000
cctaacagaatagacctaaaataaagcccaaaacggaaatttacatatctaatttctata
cataatttagcttagggggcgttgccttcgaacccccaccaaacgctttgcccctggacc
ccgactcggttttgaaaaactaaaaaaactagtccttaaaaacttgtttatgtttttgaa
atttggccaaaaatccaaacgtgtcctcaacaaagatgaaaacaaaggtagaaaatttga
gagaagacaagtataattttcaaaaacaaaaaactaaaaccaaaatggttatgagacgtg
acctgctttttttagtttttacaacttaattcaatttttttaaaacatgagaagaaagtag
acaacaaaacaaaatttttttataagtagaagtagtgtttataagcttaattttcaaaaa
taaaaaaccacacaacaaatggttatcaacccgagtttaactatttagcctcaaattaac
tatttattattccataaaaaagaaactatttattacataaaaatagaaatttcctctttt

atgtatgtaaccgataactctactatacagttttcctttattattatttattttttacat
atattaattttaaaaacccctactatacagttttttttgtttttttttttctttttaaa
actatctactctacagttattctcacacaaaagaaaactgaacttgtaagttgtaccgtt
gtactttactttttttcaagtttccaatatatttacgcctcttgtagaaaaagaaaaagaa
aaaaaaaaagaaaaaaaaagaaaaaattattgactcagtcagccgtggaagggtactatag
taattatccgctcgaagatgtcaatgatcgtcgtcggcttcaaaagaggcgctggctgtt
gctcaccagtcctccgttttcctccgctttacgcgttccagccttttccgaagatttagt
tttgtgtttcttgtatccacctgagaatccactgtcaattccatcttccactggtttgtt
ttgatttcatctctctctctctctgtgcgccatggactgagctaacgatttccggttgtg
gtgagtgtctctttcgttgactaacttaagcaccacgcgcaatccaatcccagttactcg
attgatcgattgtttgttgattgctttagagcctatctgttgtatgaattggatgctgga
gttgggaagtgggggggttttgtttttgcttgcttgacatgcatgactttgtgtaattgac
tgtttgaatggtgtcggatgacattgggaggattgggaattgtgtatgggctttgaatgg
attggaagaaaggaagtgattgtgtttttgaggttttagtttatggagagtgttgatttt
gttgtttcttatgtagattatcattattattatcttgatatcacctgagaaaccaggaag
ctttggaattgggcttttggaaatgatttttctctcagcgttcgttgagagtcccatggt
taagatcttagttctttggtttttatgtttggtaaaagttgtgaagaagtgatgggtatg
gaagtgtttcttcttctaccctgaaaaatgtatcgatctaatgggttgtaatttgtgggt
tttacctttctagaaattgaagtgcaatttggaagcaaaccggttagagtgtggagcgcc
taggaaaattgcttattaacattaaataagatacgatagacaatttattattacaattgg
tgttctgacatgctaaactcctatggtttatgttattattttccaatcttgttcttcact
ctggcgagttgatcaatttttttataagttgtacttcaacatataagtttttggaaattg
tagtttcacattctttttgttcaaagcatgggtggtgggcttctccagtagccagtgacc
gtttattatattagtggccaatgtgaatagtatctaacttacattttttttttctgcatg
agttcagatgcttatagaaa
>LcCNGC8   +   Up_Stream_Len 2000
gtggctttaacgatcacgtacgtactcaatctgttggtagttttcaattatagagactat
ttataaagttttatccgaccataaagacagaattctaactttaaaattataaagactaag
tttctcccaaatatggaatataaatctatagtttaatgaaaaacttaatagtgcataccc
tattggcaagtgagtgataaaatatttttctttttatgagtgaatgttaggcctatttcat
caagttaaacacatggaacactttttactttgaactcaattaatagaatgtattttaata
ttacaacatattatcttgttcattggatttaatctgtgatatggctattcagttttctaa
tttaactttctttcatatgaaatatgcatatttctaggaatagaaataggatagcaaact
tattaaccaaggatgcgttagctaaggatagattcatgctatggctatcagattttccat
catggttattatcagtgggtttgtcaactcattgcaaatgtacgttgtattgtgactgtt
tgtgttaatgataatatgtgttgtttctcaaagaaaaaaaaaggagaggatggaaacccg
aaacttttgattgaaattatacgttttaactatcttcttttttttcctttttttagagcaa
cgcaactatcttagttatatgtctaaatgatttcatccatacaagtaacattatttaaat
tgtgtcaatcccagtagttcaatggataatacattcattatcattaaaaaaattcatggt
tgaatctctactcacttcccttaatttattgaacttaaaaagaacatattattgaaagac
aatatttggctgcacaaccatatatatagaatattcactattaaaatcttgacaaaaaga
ttaaaaaaaaaatcattttctacctttctcttctttagttgtcaacattttaatttttaa
ttttctatcattctctttttttaattgtcaacattttaatggataagatttttctacggat
gatcctctaaaatatgtggcaatgataaatgtcacacattttgaaaacaatgatatatgt

cattttgcttgcataagcaacttattaaaattactattttattctctactctaacctcgc
tccttctcactactctcgctctctctccaatctctcactttctctctctaatctcttgct
ctccctccaatctcgctctcactctccaatttctctctcccatcttttactctctctatt
ttctctctccaatctcgctcttaaatgcgcgttttcatgtagggagtaaaatggtaattt
tgggatatatgacgaatttgattgggaagttcagtggcaaaaagaaggagattcattagc
tcgttatttagtcagacttggtgagatgacggaagccataaaaattattcaacaagcttt
ggaaggaattccaagggggccttatgaaaatttagaaatacgatctttttttgctaagacc
cgcatagaaatatgctactgatgtgagtaaagctaaatcaacagtagtatttatatcatt
tgtgggtacggctaactctccgtgaggtaactgtatgagtttccaaggtaaaagagcccc
tgaccaattcaaaacaaaaataaatagaaacatagttccgataactaaacaaaatgggtc
atccacccaattagttcttaatggataggtagaacggatatggagctaagcatttttgtt
aattgaagtgtgctgagaatagtaatggagagatacgggagaatagtattcgattctgca
gacaaaaaatgaaagccccaccaccgcctccctctgcatatcctctccccctttagcctt
taccttgcgtgattattcctcaacctcttcttcttcacaactcaatactctcttccatta
ttattatttctcgtgtcccttcttcttcttcttcttcttccaatctctcccatggtgctt
ctctctccttccttctcccc

>LcCNGC9 + Up_Stream_Len 2000

aattattaaagtctctataatttaatttaaatctctccaaacatctttgtgattatcatc
aacaattttcatacgtagcatattttatagacaagtattgagagatttaatcgaatcata
gagattattacgaagtttctaaatcatagagattataacaaagttttcaagaccaaaatg
attatagcttagtttttaaatcatagggattttaacaaaatctttagaaccaaaatgaaa
tcaaacttagaataatacatactaaaaatgataatataacaaatttatttttttaaaaaa
attgataatataacaattctttgactttggcatgtgagatcgcacatgtaggcataccaa
tggacttgggaaaaggcaacaagatgaagaccatcagtgccatgttctttaaatgatgaa
catttcagagaattcaagattcaaaatggatagccaccagattctggcgaaacagaaact
gggcttctctgcttcattccgagaagccttcaaaatcatctttcatcgtccaaacttcat
tactctcatcatcatcatctacatccctctcttcacctctctcctcgcccaccaaattct
ccttcacccaaccttcattcaactcctcaagcttctatctcaggaaaatccttttgatcc
atctcatgtgtacgtcatcaggtgtcaattaggtgatggcagagactgcttctcacagcg
ctcttctgataacaatagcttccaagcaactctctcccacaggtttttatctcaaccct
tctctctttaggaatcatcttcattcttgaccttctctccacaatctcaacggtctccat
atcatcagcattatatggaggaaactcccaaatgggctttaaagagatgatagttgaagt
cagaaagctggtggctctaagactgagagggtcattggcaacatctctatatgcccttttt
gctagcttcactcactcagttgggtttggttgctttgtcaacaaacatgattttgatgcc
aaaaagctcattgatctttgggccaatatttgcatttttgttgacaaaatacatagagtg
gagtgctgtttggaacatgggaattgttctctcaatcttggataagaaccagggctacat
agcaattggagtggcagcatatctaagcagaggcagcaggaagctagggttcagtttgat
gctggttttctttgctttgaaggtggcttttgggctgccatgtctctatgctttgtggaa
tgaggggagttgtggggttttggggaatgtggtgtctgtgagtttgaagtgtgtgggggga
tgttgtaatgtggattgttcttatggtttatttttatgactgcaaaaggcagtttttgga
caagaagatcgatttggagaataatgggaaagtcatcgaggctactcaaccataaattga
atcaaatagaattgtgtatttgaatcacgtactatcaaataagttcaagagatgagctca
ttattattcttgttattatttcatttgcaaaagttaacagtagtcaagaaacataaattg
gtttctccttcacctgctcaatcctccatggaagcctgcaaattggtgttaagaagctct

gttccaattcatttgtagaagaaagagaaacctcccacctgccattatagaaacattgga
gtataattagtatataaaaaggattgaattaattgcaaataatcagtgtaattttgagta
aattcaatctcacccgtcatcaaaacgccctatttttagcaaatgttcagacaaaaccgc
catggccaagcatcaaagttcttggctattacacatccacttggcccctcccccatttcg
catgcaccaacccaattatattctcttttcctcctaagcttctcatcttctttcttcctc
ctcctctctccatcaaccctgtctctctctcacatttctcgccggggaactcgacaaaac
cccggaatccgccatggaag
>LcCNGC10 - Up_Stream_Len 2000
aaattaaatttgaaaaactaaaattcaaattaattgtttttaattttcaaaatttataat
ttgaaaatatttatcataaccacccgattcacttgaaccctataaatagtggtctaaacc
aagagatggggtggagcttttgtggtgcatgaagaattaaaaaaattgcagcaaacatca
accaagtgggatgagacccattcctacttcatctttactaatgtgagtatagccttttca
tttagtgtaaatatatataatttttttcttatttctttttcaagtagactaatgtatcctt
aggagattttgttagttttatgagattttattagtttatattatattttcgtcacccta
agagtttcatatgaataagaaatatttttttgtttatttctttaatagaagcgagtacacg
tttgtgtgagttcattagtattgtctttcttattcttgaaatgggaaagaagatcgatca
ctattatcttcttttttaaaatcgatctctctccattatgcttttctccgaccttcaaa
atttttcatatatttttgagattttttagacaagatgataggaagaaaatgacattttt
atttaattagtgctcatttaagggttggattaaatataactttaattgaattattaatta
ttaattgattacaaaattaagattttggaaaatataaatgcttacctaggtaatttaaaa
gacgttatcttcaatcttaaaaaaaatgtttaagcgggtgagatttttttatatttgattt
atatctatgatattggttataaaccatttttattagaatggttcaatttggtcacataca
tatcaattttttgcaataattataaattcatgtgtatttattcatttttatattttttttg
gtacattatcaagtcatatttcaaattttcttaattaactacttatactttaaggtatta
taacacgtgcaacgcacgtagtttcaaactagttaatctaagtggcgtcatgcatcatgc
aattcaatttccattaattaacatatcacattcttctcaaaaaaaaaaaaaaaaaacatat
cacatatactaaggtcccgtttgataaccatttcgtttttggtttttggtttttgaaaat
tgaacttatttctcccaaatttcttaccattgttttcatcttgttaatgacacatttga
attcctagccaaattccaaaaacaaaaacaagtttttggaaactactttttttagttttc
aaaacttgagttggtttttgaaaatatgggaagagggtagagaataaaacaaagaaactt
attaatggaggtagtgtttataggcttaattttcaaaaaccaaaaaccaaaaacaaattg
gttatcaaacggggcctaaagatatcaaactgagaccttaactaaaataaatattattga
tgcatagggctttagttccagcttcatttgtaagtttgtaacaacctttaaatatatata
tatgtaaatactgaaatgcaataacaatcggagactgaaactcaccgttgcacagaaaag
aaaatcacagaggatcgctgtttgaatccaatcagaagcagaacccaaacgaatgtctct
atgcttctggagatggaaaatcaaaagacttggcgagaagaaacaagaaaacacacagac
aaatttctattttctaaatatagaaacgcaatcctgctgcgagaaaattggatctccaat
ggaagaaacacacaaaaaaatgaagcacaaacaattccctcccaccgcatgcgaccatttc
aaaccgtacctatttcctctctccggaatcccaaccgagaaaaattttccacaaacagtg
aggtttcgatttgcagtaatcggaaaccagaatgatctaattccataacagaacggcaga
gatttgaagcgcgatcgaattggcacagtttgaatatctcggcgatcaactgcggcatct
atgttctccattgccacaaa
>LcCNGC11 + Up_Stream_Len 2000
tatataggtttgaatatccacatctctttgtcaacgatggagctaaccaaaagaagagca

tgcagagaggattattgagaaatgggaagaagggacaaagcacttaaaaccatagagaat
gaaaagcatattaaaaacagaaaagtgtggattggtgaatgtaggagtatcattgatgat
agatttagagtgttattagcatgtaaaattaggattattgagactaatacatggcattat
caatcttattttgaaacatgaagaaactccgaaacatcatatgggatttgcttagttttt
gtttaatctgttatgtggtgaaaccatgagcaaatcaaattaacctttgtctttcactat
ctaaagctgttcctctaatgtaaagctgaacattgtaaaacagagagtgttgagactgag
agacttatctaaagctgtttctctgttctttggtatgggttctcttcattttgcaaaggt
tcaatgtatcacattcctttttcttgagtcatgtgataggcacaacaacactaaggtagta
aaatatatcaactaaaccaaatacaaacttcaattcaatataacatcatcaagcaatact
atgatgtttcagaagcaatactaaagacaaatggtttttgttgacacgagtttctagtca
caagttcacatatatcacctgatcgaggtaacgagccgaaacatagagacgatttgcaat
agaatgctcacatcggtatggtgttgtgtcccaacatgctctaatgcttaagttggtact
taaatcgagataatgttgagctaagctagagtgatattgagtaatttgagagcaagatgg
atcccaatattccccactgtcagatgttggttacatacatagtagcggagattttgagat
taagcaactgaagcttcaactctatgtttaccaagattggtaggcctctcctacggaccc
tcgaaccatgtttcctcgacatagtgagtccggtgagcctcctcacccggaccctgtggt
gtgggatccaagtgggagcttacatggatacataacagtttttatactaaatacctacca
gatccttaatattgaataattcaccacatagcaaatgaaatcatgttgatagcaaatgta
aattcataggagaaaagaagatataagctcattaggccactgttcacaaatgttatggtt
tctggatgagatgagtcaggacaggaacaaataattgagaagggtgggccatttcttttt
cttttgcactaaaaagttgttctcataaggtggagtttatttaaacactaaaattaccca
tttcaaaagtacctctctcttttcaaatggggttttctagcaccaaagttgagagagagt
gcctgagatacgaacttttttttttcttttttttttttttcagattgagctaagaattcc
atacacatccataacaaacacaagccccaataaagccaaagtctagttgtttataaaatg
ccataatttatttctaaaaagagacttatagagtcaaataaagattggtttaatgagaca
atttagaaactcctcttttatgccaaaattattaaaaaaagactagggtatgactaaaga
agagcccttgcttttcatcccaaatcagaagggttgtaactatgacacggtgaagtggga
gagcaaagcaactttatgaaagagcacttataacgtctaatcattttcttggagctcaca
ttggccattacttcaccagctgatagaagggccttctggtttcaattcaacgacccaagg
gaaggtttttacttcataagcttaagttccactccttgtctttcatgcaagtgaccatat
tttatatgaaaggaaggttaagttttcatgtaactgttggttgtgtatcttgtttttcac
tgaaacattttgcttctgaagctaagcagtagcataaagcactatcagagcaacagttta
gcctcacattttgtgtagtg
>LcCNGC12 - Up_Stream_Len 2000
ccaaagtcctaacaaccataattaattatattgactggtttagatatatttttatattta
atatattaaatgattgtgaagtatgaccgtgaacccaattatcatagaagaattgtatta
cttttacattatgaattcatcccaaagtcctaacaaccataatttattatattgactggt
ttcaaagtcctcatttacaaatatatatattattgcttctatctcaaatctcgaaaaacc
atgatttaatatatggttaatttgagtataagtcaactcatttagatatcttgttaataa
aaaaaattaaaggcttatactcaactgattaagatatatgtttgaccaaaagatcaaagg
tttgaattctccattctcacatgtggttgaaccataatttatattcattgatcttgaagt
cacgagttaaaacaaaagttatttttatccttgaaaaaatagaaagctgattttttttta
acttttataaataataatatattttagaaatagtcacgtgctaaaaattaacaatattaa
aaataagtttggacaaaagagattaatgtccttctcaaaacagtacaaacacatttattt

ggaaactattattcggaataggtttgaagatagtagcctttcctcaaaaaaaaaaaaaaa
aaaaaaaaagtttgaagatagtagccaacactctaaaattaataattttgtgtaccttaa
aaatcaactttagattttttttttaaagtaataaattaggaatttttttttttttttttt
ttttttttgataatatgtggggaaggagattcgaaccacagatcttgtggttatcagtac
aactttatgccagttgaggtaagctcttattggcttggacaaaatttatatgaaaaaaaa
agtctcacattgattcataatagagacattacctctttttatataataacaaatattttt
cattttttttttcttgaaaatatgtttgtctaaccaactaccatttttgtccggagagtga
agatttgaactccaacaccaaaagaatacttacattctttagaatgtgttatgacgaaaa
cagtaacaacgtaacaagaatgaaagggtgaagagaaacaacacacacagatatacgtgg
ttcactaacggtgtgttagctacatccacgggcagagggagagaacgtcttattatgaga
ggagtattcagattacagagattagggggaaaataggttaagaagatttatatatatggc
accactcttaaccctaacacaataggcccaaaatagtaaagcccaaaacggagaatcata
atctaatctctatacataatttagctaacgcccccgaacccccaccgggcgcgtcgcccc
tggaccccgactcgctgacccccgtacttggtcgttcggagcgcttcatattagattcaa
ggcatatcaacagaatgaaattttgttttacgaacctaattcaattgattatgattatat
gatatatatcatcgatcgaaaggttaaagatttgtgatccatcctatcgacggaaaatat
tttattttgttttaaaagatgaaaagagcttggactcaaaaaaaaaaaaaaaaaaaaaaa
gaaaatgatgaaaagagcttgtgaaccaacccaagcctatctttttctttttttacatgc
aaatgcaaagtgcaaacagaaagaggaatctttcatttctctttcttctctatgatattt
tggagtaaagccattagagaaagaggtgataacggtcccattcaacatcgattattggtc
tgattaaaacaaaagcatctctgtttgttcatgttcatatacttttattatttggtcgat
tatggcggctcaacgctcgttccagaattggaaattgtgacataactaccggcaaaaaag
ctttaaattagcttcccggcaacagctctaaatcagcttcattcctttttttctcctacat
gtgaactcactcaatcggcc

>LcCNGC13 - Up_Stream_Len 2000

cactttttttttttttagtttgcataacatggaatttgttttgatccaaagctatgagact
gcctaggtcttgatcggtgaaatcttggattgattagtacactataacttgacgaaagga
ctagttagatataaatgatcttgatcatttaaatcatttgtctaaatcatttgtcaaccg
gttgataatttggtagagttttcaatatgaacggaacttggtgtttagggtgtgtaaatt
tatttaataggaaaaaagatgacccacacgatgtggtgcttgctataaacacttgaatgc
ttaaatcaaagaggtagaaaaaaatgtaacaccctccttgaagtaagagatagaaattgc
atttgacgaaattataagaatgtatgggtaagatgatttgaccaatggtttttggtatta
gaatatttggttgattcattcatctactcttcatgggttgcctttcgatcataaagttaa
ggacttttttttttcttgacaattaaagttaaggacttgttatattgttcatagtattta
ttgttcatagtagtcaaattaaaactatattttgacccaaaggaatttcttgttcatcta
ctcttcatgggttgcctttcgatcataaggttaaggacttttttttttttctggacaatta
aagttaaggacttgttatattgttcatagtatttcttgttcataatagtcaaattaaaac
tatattttgacccaaaggaatttcttgttcatctactcttcatgggttgcctttcgatca
taaggttaaggacttttttttttttctggacaattaaagttaaggacttgttatattgttc
atagtatttcttgttcataatagtcaaattaaaagccaaagcgagcatagctcagcggta
attggcatatacccttgaccatgaggtcagaggttcgaatccccccacccccaaatgttg
atatactcaaaaaaaaaaaaaaaatagtcaaattaaaactatattttgacccaaaggaat
tttttggaaattcgttaggatttaagacgcgggaagcttgtcgtttaccatccaaagatt
gacgtatcgtttgggggcaaaataaatacaatatcttccttgacgccaggccctttaatg

tcgtctctgtcgttgacaaatcccacaaatgcagagacaaaactcttcagtccaaaccca
ccttcttcgaccttcgccgcgggtcccaaaagcaccaaatttctcatttccgatcatcta
aaagtcacactcgccttgagtcgaccagtcaattttcagacgaaacttttatcattcata
atggcagttcggggttttccttgcaagttttatagcttcaacgagggagtcggtgttttc
tttaatttcttgctgttccctttggtgggtatggttgatttctcgtgcattacgaacttc
cctaatctgggtaatgagttttggttccgatcgtttcgttttcagtgttgtggttttgat
ttttgaagttggtttgaaatggatgcttggtgatcttactgtggctatggataatcttcc
tgtatttcctctgtgtgatttagtgggtttctgttgctttttacaatatgatgtaagcag
ttgatctttttgtacggattcttcttgtgggtggtttcatgacagtggagtttatgaggt
tgaattcatatggagagactcagaatcctcattttcaaactgacttctaattggatttct
ggattttgtttgccccttttttgtgtgcttgtttctatgagttgatttgagtgcatgtt
cattattttgaagaaatggatgctcatagcttcattgagggtagtgaaatctgcttttt
ctcttttgaatcttttcaactattggtcacttggataggaccttgatcatcctgacttct
aatcaatttgttactggtcaggtttcaagatcccaggctagagaaaacttgtggagtaga
gtttcatagtggatgagtaa
>LcCNGC14 - Up_Stream_Len 2000
gatagaactctatcacttatagaaagactttctatcagtgatagaagatttctatcaatt
aaaaaaaaaaaaaaaaaaaaaaaactgacgataaagaagaagatgcgcgcgccaatcacag
gggcaatttaggaatataaaaaattaattaaaatttttttgccatatttgcaaatattttt
aaaaattgctatatttgccattatttaccctcatgttgtcacccattacaattatccttt
tttttatcctatttttttgcaattttcacatttatattattacattgcaaatgatttgaag
agtgtcgtctccaagttcccgcggcgtttcctttcaggccagagcacaaagacgaagaga
aaaagaaccatgaacatgccgctattgttcaaggtagaagtgggaattcctaaatttcca
atggccgccattctttcctctgtaatatttgctcgttcgtcacgccaaagacggagaata
tgtagaaatggaagcaacgagttgccagaatgttgcaacaacgtctgagaagatgtcttt
cattgcatttagtaactgtattctcttgccgcgaaatagagtcgacctcgttgacaaaat
tagctgctgcaggaatccgaatcgatggcgcctactgagaattagctggctgcactgcaa
caatgcaacgacgccgacgaagccggaaaagatatcgtcggtctcgttgaaatttcaaag
gtaattcgatttcaggtttgctttatctttcaatttcatcgacattttctttgtagaatt
gagattttcccaattacacttgagaactatcagtaacaaatatttcattaaattatttgc
aaattattgacttattacaattatatagctaacagtacatgtcaacagagaattaatagt
ttaaaattctatattttactctttaaattttgaaagaaatctttgttaacgagcagatta
gtgttaatatactaggacacatgccattaagaaagaaatctttgttaccgcttgtggtgg
aatactaggacacatgccattaagaaagctatggcaatcaaggaaggtttgaaaaagtac
cttaatctcttcaaggatcgtctgcaaccgcttgtggtggaatccgactctctgtcgttg
gtgaagatcctcaacgatcaatccccagacctctcaaaagccgaaatcttggtggacgaa
atcgaggatatcacgagggagatggggaatgtgtcgtttgtctggtgcccaagatcgtgt
aatagggcagcgcatatcctggcagctaatgccgcaaaatccgtgtcgattccctctctt
ttgatcggattttcgtccaggcctgagggccatcctttttgtgtacagaacccgttttcc
tccatggaggatgagttaatttttttgtgggtctccttgtacccctctttggctggcctct
attataaatgaggaagctcttgtttgttgtggtggttgagtttgtttaatgaagtatttc
ctttttaaaaaaaaaacaaaagaaagaaagaaatctttgttaccgagcagattagtgtta
ataaaattgaaaatttataatgcattggaaacaaaattgagagtttttaagattaacgac
gagagtcgaaagagtcaacaataacatgcagaatacgttatgcaatgtcggtagtgcacc

ccatgagagataatgtttatccataccatactcctaattaatgttaggctcatggatcac
catacatttctattccttctacgtcgaccctggattgtcaaatgaaggtaacgccttgaa
taggagaggaagcatacaattatttgagtcaatgttctatgaatggaccacatggaaatg
ggacgcgttcatattcaacaaccaatcaagggggactactttaatggtggatgaagccat
ggacttgttgagtcaacgtgattactggaggatgctgttgtaaggatattcatcctgttt
ccagtaagaaagagctcgct
>LcCNGC15   -   Up_Stream_Len 2000
taatatatatatatattctaatagtataaagaagattaccaatgctttaaccatcaacga
tatgctcagataagctaagaaaaaagaaaaaaaggaccattctccatagatatattgaag
acgacattcacactcacatcttgagtaaatcaaagaaaaataatacacaaccacctagtg
tatcaactttttttgaaatgatacaaaaaaagatatggttgttcacaacttaaaaacttt
ttttatgatgaattagataatccttggaattatctcaatgaacaaaaaaaaaaaataataa
aaaaaataaaaaaaataaaaaaaattgataaatagaattcaagttctggataagggatcc
ccttactctagctcggaatgtactggaaaaaggactcaattatataatgataaaactaaa
aattaatactatttaccaaaaacatatgatatccatttttttttttcattctcaatgat
aaatgaaacttctataaaaaaaatgatagtgagagaacgtttggacaaaataagattcat
gctatctttcttaatactaattacccacaaatggaagataaattgcacatgttagactcg
gaagtaaacaaacgaatgactatttgaggagaagctaagctcgatcgagcgtctcgagca
caaggaagtttgacttttggtaaagttgaggatgagttgataaaaaaacacacaaaatta
aagggaaatttattgcattgaaaccgtcaaatgtaaatgtgattgtcttttaggcttgct
ggcatgcaaaatataaaaaaagtgatactaaaattgttgtgttctctctttctctttctc
agtgatgtgtgtgtggtaaaatatttggagttctgtgaggctcacacacagctgcagctg
catcaatgcaacaaacaaaactgcaaaactgccatgccatttcgtgtgtccacctaccat
atatcctaactctcactgctcgccacaacacaacacactatcccctccttttcttatcca
aaaatactttcctactcatcacacaataacatttgtatcactaagatcccgttcgatagt
atcgtcgtttggaacccgtttgataattattgcttgttttcttctcacaatttccatata
atggctaatttcacgtttcttaagaaattatttatattcttagtcaagttctaaagacaa
agacaagttttcaattttagtttttaaaatttggcttatattttgaaaatattggtaaaa
agtagataacaaaaaggggcctttggtttttgcgatgctaatgctgcagtttgggccact
tgatttaaggtttttgatgcttatgatgggtcttccatcacccttaattggaaggccttc
atatagttcctgtttgagtttctcatacgatcaataaagtattcccttttctaaaaaagg
gtattggttttgtattaattaccaaaagtagatgacggaacccagaggtgaaagtgtggt
tataaacttaatttacaaaaacaaaaaatcaaatggttattaaacggagcaaaattaagc
ttttatacaccacttccatctataggtttctttgtttgattatttacgttttacaaatat
tttcaaaattcatgtcaagttttggaaagtgaacacaaattgtttttgttttattggatt
taactaagaaattcaagtgtttacttctcaattggcataaatcttgtatactatttttat
tctaaatttgctatcaaacacatatcgaaaaaatatcgaaaacatgaaatataactatga
ttcttaatgaattccttgctttcaaatcaccaactaaacgcaccgatcgtagttttcgat
atttccaaaacccttcccacatgcaaaatcaaaacccctttttcatttttctctctttctc
tctgatttcagttgtttacttttacacttttcctctctccctttttctttcatttaattt
tttttggtctgtttaataat
>LcCNGC16   +   Up_Stream_Len 2000
ttcattttttaattttttttcttttttttttctgttatttctttctctttcctttttaattt
ttccctttttttcggttatttctttctctttttttttttttttttaattttagtaggttttta

atttatttattaaataaatcaattaacaagatattttatattcgccattaattgtctcct
tttctgattttacgtgtcaggatcattaactaatgagaaatacatgcatttttttaaggaa
aaaaataaattttattattctattcaagaacggtaagtatataagcttgattgtctaacc
ttaaataaatatctcactatcacaagcactaaaaaaaatctatattaatagcatgttgtg
caataaactaatatcgttgaaaatttacaaagtttgtaaataacaaattagtctaattga
actaacttatgtaaaccaacatcgttaaagataaatttgattggagtgagagacgacatt
gcaaatcaaatttaaacaacatttgaaggataaaaagttgttatttttttattttgtgttg
gagctgaataaatactataaactttgtataatagttttatatttattttaatgtatgtta
tatatattttttttataatttttgtactaaattttatttcaactaaaaaaataagaaacgag
aaacggttaataaaaaaaattatgtttttgtttttttttttagaaaaaacaagaaacaga
aatagttatcaaccatatttctatttttttattttttaaaaaataagaaacaagaaatgaa
aaaaaaaaaaaaaaaggaaaaatgagaacgtaatcaaaccggtcattactccagaaagag
atttagtatatataatatctcaccactcagctggccaattcacaaattattaaaatacac
acacacaagaacaatagtggtcgaatcttatattgatgaaagcatctcttatttctcaaa
gagatggttcattcaaatgcaatatttgcgtcttttatgtttttttttttcttttttgatt
taacaaaggtagagtaagaaaccgaactttcaacctcaaagaaagtaaccaatgtcgtgg
gaggataaatttgagattttcgtacggatgaccctctaaaataggcggaaatgataaatg
ctccacctttttcaaaacaaggatatatgtcattttgcttgtgtcagcatcctagtaaaat
tatcattttactctctactctaacctcgctctctctcgctactcttgctctagcttcttg
ctctctctccaatttctcacactcttaccaatttctctctccaatctcttactctctcac
taatctcgctctggcttctcactctcactctcactcttgctctctctaaattctccctcc
aatctcttactctctctctaatttctctctccaatctcgctctcgctccaacctctcact
cttaaatgtcttaaatgtgcgttttcatataaagagtaaaatggtaattttgctaggatg
atgacgtaagcaaaatgacatatatccttattctgaaaaggtaggacatttttcatttttg
acctctcaaaatgggtcatccatacaattgtttcgagaaatttttaggtgcttccggtgt
aatgaggtcggtagttttacacggttgaatgtaagagagagaaatagtaaaagtatctcg
attttgtattcaacaattattaggggcagacaaatttgaagatttacattcgagaagtct
cattgcaccggaaacatctaataattttggcatggggagttgcatatgttgagagaggaa
ggagaaagtggtaaattgagagggaaattgtcggtattgatgaaatctgccttgggtttg
gctctactacatcccccagtactgtaataattcagcctttgttgtcaactataccttaag
agactccaaactgacactctctcacacaacacaacaaatcacaatacgtctcttcctaaa
ccatctctccattgcccccca
>LcCNGC17 - Up_Stream_Len 2000
cttcaattttatgaacacaatggcttcatggatttgatcaaactttttgaccctttacac
tgaagaaaacttgtagaccacaccattcaataccatagcattgcttttatatgcacgaca
aataatgccaaaaaacttgtatatgctttatctgagtgtaaaattgatttttatgatcct
actccctttcttgtaggaacacatgccttatttccgacttagaacagcgaaggaaatttt
gaatttgaaggctaattgattgcatatgtgtacataaaagggaaaggaatgcgaggagct
tcgaagataagtctaactcttttgatatttttttgtaatattgtataaaatacagcctct
tggtaggtgtctttacatagaaagttcttttgtaattacagtctgctcatgattattagc
gattggcagactttttttgagatagtttttttgggaggggagttctccaccccggcctttta
ggttgttttttttttttttttttttttttgtggtaatatatcctttttcgatgtttctta
tgaaaaaaaagagatgatattttttttgcatgagtggcagcgactctaaattgtaccgttt
tgaactggttgtgagcagtgtgtaaaatttcgttcgacatagtcaacatttcgaggggga

gaagtttcgttcttcccccaatgtcgacaacatgtcattttgtgtcggtttttttttaat
tatttttaatagttttttagtttaacatttaatattaagcttttagttgcataactatc
aattgtcccagtgggtgtgtgcacgcctttttgttagttgatcaaataaaataagggaat
ttttaaacctattttttaaaattcaaggactaaaaaggtgcattttgaaaacttagggac
cttttaaacctatttctcaaaactcagggactaaaaaggtctattttgaaaattcaggag
ccaaacacacctattcctcaaaactcagggactaaaaaggtatttttccctagtttataa
ttcatcaagagaaaactatatatatataccagagatggcacaggagaatttgaacctaac
aagatacaaactttatgtatgctcatgtcgccagagacaactatttctaaccatatttgg
agttagaacttatcaaagagttaagattgcaacaatttttttttttctttgagaagaaga
ttgcaacactttaatggaagcttagggtgaaacttgatttttttttctccgattttatta
cctcaaccatggcctgcgatgtggacaagctatcagttcatcatcaattccggatccgtg
gacaaaattttatcgctgatcttgaaatttctaacctaattcgatcgaggggaggtttaa
ttttcttccttaattattgccttctgtcaggtttactcatcttccactgttgttctctct
gaatgtttgagatttttctgattgaactttagaatgatcagtaacagaaatatatcttga
acgattcgttaagtatcgacttgattacaataagaaatgggaatgattctctttgtatta
ctgtaaaaaaaaaaaaaatgttctatccataatagaaacgaatatttatgattaaatgag
tacttacatttgaattaaaatggagctgaatttcgatgtagactactcaacagacggcat
acttgttggctcttccatgggataattgttgtgcatgtaggcccttcaagaagaatctga
attaacggctcgtggattatcgtacttcttttcttgtttgtttcatctgcagcctcatcc
ttctgcgctgtaagacaaacgaggttggcccctccaatggcggaggaagcatactgtcca
ttcagtgaccgtactgtagccctcatgggtgacgttcgccaacctatgaggatggctttg
ttggcgggcgtggccatgtattttctcgagctaatgtgctcacaggaggatgttgttata
aggatagacgtcctgtatac

>LcCNGC18 - Up_Stream_Len 2000

tgataaaataaccccaaatctgagctgtgggttcttccatttgaactcatagtttttagc
gttttttcttctgccagctcttttagtgggaagtcaaccgcaaaagacaatcccccatca
ttatttctcctccatttgggcgcgtccattgcgggatcctttctcttttctctcaaaaat
gcttttcccaatgtgaatctttccgttatttgctattgtgcagtggcaaaacatttcttt
tctttctcactcgctttgcttcttcttcaagatttcaatcttctccaagttctagagaaa
tttgttgctcgtttttgagatgctgaaagcctgaaatcgagccgttcccttaatgtaatc
ggttctcgtttagttcaacagccatggttgaatcgggcatttgactgaacgaacaaaaca
agcatggagttgaagaaagggaagttcgtgaggtgatttttctctacagattcttcgatt
tctctcgtttttcttcgtttgttcttcttttcgtttgatgggttttcgtttctttaggtt
ttactccaatgagaagcagaatctcgagcttccatgggagaaaaccgatgctaagaggcg
cattgagtttccattaccagttttcaaaatgggtactgctccattatcgaaattagacat
tggggttggggataaaagtaaaattagctcagatttagggaggtcgaagatttttgtgga
gaataatggggcacaacagacgaggattcttaaccctgaaagtgaaacgattctgagatg
gaacagagtttttctcttctcttgtttgacagctcttttcgttgaccctcttttcttcta
ccttccttctgtgattcaccatggcaggtcttcttgtatgactactgatttcaatttagg
aattgttgtgactgtttttcgcacttttgccgatgtgttctatctgttgcatatggtttt
gaagttccggatggcttatgtttcacccacttcaagagtttttggcaaaggtgaactagt
cactgatccaaaaaagattgctaaacgttacttgaagtctgatttctttatagatctcat
tgcttctctgcctcttcctcaggtatttcacacttttgtttggtttgtgttcattcgatc
tttcgtgtttgaaactcagctttaaagcttaggtcccgtttgataacgatttaggctggt

tagctcttaatgtttttgcgatggttttcacctttgaaacacaaaaactacttgaattct
gaaaatactcttagaaagatgatgaaagaagataaaacaaagaaagtcatgagttgaagt
aatgtttataaggttggttttagaaaagggtgggcctttgtgagtttgtgtctagttttc
tgtgaattgtattgaaaatcacatgtttgagactttgttatcaatcaattttgaactact
gtttcagtctgtcccaatatgattggctttgagatttggagtttctttcatgtaggccca
gatttgtgtttgagctgtactgcagctgtttcatcagaagcaatatttttttttccatat
tttcttttagttccttagctttcttgtttgtgtccaataggtttttgaacattaaagcta
atagatccatgaactttcaatgttgtgtttaatgagatcctgaacttttaatttcgtgtc
tagtaggtcattgaatttcaatttgtgtctatgtcatggaatttcaattttatgtctagt
aagtcattgatctattcaacattttaaaattcatagatctattgtaatacaaaattaaa
agtatagggtttagagacaaatgtttaaggttcgacaaaaattagggacctattggacac
aaaattgaaagtttaggaaattattaaacatttttgaaagtttagggactaaacttgtaa
tctaacctttttatatccaaactgaggtcaaggatcatgttaccccttattgattctttt
aaaacttcttttggcagatt

>LcCNGC19 - Up_Stream_Len 2000

taacggcgttacatctagtggttactatttcgcagtccggtcttatgcaaactgattgca
taggatacccccacccgcatgtctcctacatggatgctttggatcattgcatctgtattg
tgaatacaaggtgggtcgtatcacatagtgtcaccaggataaggtacccagccttatccc
tatactatagaccttttaggttgttacttggacatcgatccatgtatgtccccacatact
agttcaagtttcatttaaacaaccttggatcttagtttattggatttagggtttattaat
acaaactcgattattcaataaccaaattactgaatcactcaaataatgtcttattgagat
cgattaatttgtttcatttactatctacgagttttaggacataaaacccaacacaactaa
tataaatccttacactacttacacaaatagcaaaatagactctttccatcactgatagcc
acttataaatcagagatatcaatatctatcacaaatagaattttatttgtattgaagtct
atagtaactgatagacttcttctattattttatagaatcctacctatattaaggtattga
aatgtattaatgaccatagactatagaccttttaggttgttactcggaaatcgatctccg
tatgtccccacatactagttcaagtttcatttaaacaaccttggatcttagtttattatt
ggatttagggtttattaatgcaaactcgattattcaataaccaaattactgaatcactca
aataacgccttattgagatcgattaatttgtttcatttactatctacgagttttagggca
taaaatccaacacaactaatataaatccttacgttacttatacaaatagcaaaatagact
ctttccatcactgatagccacttataaatcagagatatcaatatctatcacaaatagaat
tttatttgtattgaagtctatagtaactgatagacttcttttaattatttgatagaatcc
tacctgtattgaggtattgaaatgtattagtgaccatcgccaatgaaattaattgattta
ttaaataatacaagtctatccgtggctgtcattgttagacttctatcattgatagaattc
tatatatactgagtccatcaatgatattcacctatataattatatttgtattgaagcttt
ttagtgactatcaataatagactcaatgtaaataaaatttgtctacttgtgatagtcgtt
tgatagattttaataaataaaatttagttcagttggagtcttactaatgtttagttgata
gactatgattgtgaaagaaatctattagtgatagaaatctattgtcgatagaatttaatc
acaatattgtaatcatcagtgattaaagtctatcactaataagtaataactcaactcata
aactgtcaatactataagtgatagtcatcaatagacttgaatacaagtggaattctatat
gtgatagtcatcaatagacttgaatacaagtggaattctatatgtgatagtcattaatag
attctaatacataaaaattagttcaattacttttcatttggattcctccatctgtgtttt
tcaccgatggccatgataaaaaaaatagataaaatcctaataaatgatactagtctatca
atgactatcacagatagaattctatcagcgactatcactgatagaataaaaggcaaaacg

ggaataataaatattcatttaacatatttaccatatttgcaatttttctttttcaatttt
gctatatttgcaaataggaaaacataaattgctacccattacaattatcctttcattata
ttataaagtctcatgcatttagtttactttacttgggtgtccatctcagaggactatga
tcatgtgcaacaaagtgagaagaaagttccatggttggtggaacaatattggttgcaatt
ggcttaagagtttggggaaa

>LcCNGC20 - Up_Stream_Len 2000

ctcataacttcctcaacacttgaccaacattaatgtactataaaccaaactcttcatttt
cgaacactctacaacgtacctgaagaaatgaaaacgagattcccaaccgatttgtacaga
aaatccccgaacacagaaaacatagattcaagcttctctgctaccctctatctttctcct
gaaattaaacctatttcaagtcatttttggctcacgacttcttcatgaagtagctttcca
accatataaaatagagcttctgattcaacttgtacaccccagaatgctcaaaaaaccaga
gagaaccagagatcctcctggtttgcacgaaagttttgtgctatgttttccatttaaaaa
accattaacttatcatcaaatttaactcaagcttccttcatgaattttgttagaaaatgg
attaactttaaagaaaatcaggtctcacctcttagttcttcttgtgtagctcaaaccgaa
taaaaaataagagataggcaggttgaacccaaattctgccatggttacctttgcttgagc
tatttcctctcttcttcaacctcaaaaccctagaaatcttcttcttcttcttcctttact
ctctcccttgaatgctctgaagtttgggtggaaaatgatagaaaaatgatagaaaaatgg
ctggaaatggttgaaatgaatcatgtcgttggtgggattctccttaaaagcactagcacc
gaatttccttttttactagtttcttttcttttctttatttcatttcatttaattcttttct
tattttaaattagtaaattctaacaacttaatccaacaatgaaaccacaataacttaaat
ccaacttcctacaatatgtgacaagagaaaattatccaaggaaagtctcgggtttacaat
aaaacccaacacaactaatataaatccttacgctacttatacaaatagcaaaatagactc
tttccatcattgatagccacttataaatatgagatatcaatatctatcacaaatggaatt
ttatttgtattgaagtctatagtaactgatagacttcttctattatttgatagaatccta
cctgtattgaggtattgaaatgtattagtgaccatcgcccatgaaattaattgatttatt
aaataatacaagtctatccgtggctgtcattgatagaattctatatatactgaagtccat
caatgatattcacctatataattatatttgtattgaagtttttttagtgactatatcagta
ataaactcaatgtaaataaaatttgtctacttgtgatagtcgtttgatagattttaataa
ataaaatttagttcaattggagcattactaatgtttagttgatagactatgattgtgaaa
gaaatctattagtgatagaaacctattgtcgatagactttaatcgcaatattgtaattat
tagtgattaaagtctatcactaataagtaataactcaactcataaactttcaatactata
agtgatagtcatcaatagacttgaatacaagtggaattctatatgtgatagtcattgata
tagatttcaatacagaaaaaattagttcaattacttttcatttggagtcctccaactgtgt
ttttcaccgatggccatgataaaaaaaatagataaaattctaataaatgatattaatctat
caatgactatcacagatagaattctaccagagactatcactgatagaataaaaggcaaaa
tgggaataataaatattcatttaacatatttaccatatttgcaatttttctttttcaatt
ttgctatatttgtaaataggaaaacataaattgctacccattacaattatcctttgatta
tattataaagtctcatgcatttagtttacttttgtttgggtgtccatctcagaggactat
catgtgcaacaaagtgagaatgaaatttccatggttgctggaacaatgttggttgcaatt
ggcttaagagtttggggaaa
