# Supplementary material for: Genome-Wide Identification of the Cyclic Nucleotide-Gated Ion Channel Gene Family and Expression Profiles Under Low-Temperature Stress in Luffa cylindrica L
Source: Int J Mol Sci. 2024 Oct 21;25(20):11330. doi: 10.3390/ijms252011330 (PMC11508470; doi:10.3390/ijms252011330)
Supplement: Supplementary file 1 [file ijms-25-11330-s001.zip › Supplementary File S8.pdf]

Table S2. Primers used for RT-qPCR

Name	Sequence (5'-3')	Function
LcCNGC1-Fq	CAGTTCCGACGGCTTCACAG	qRT-PCR
LcCNGC1-Rq	CTGGATGCGTAGATGGTGGC	qRT-PCR
LcCNGC3-Fq	TGGATGAGACATCGTCAACTC	qRT-PCR
LcCNGC3-Rq	GATACTAGGCGTTCACACATTG	qRT-PCR
LcCNGC4-Fq	CGCACTTCTAATCGGAAATATGC	qRT-PCR
LcCNGC4-Rq	TGGCGTTTGATGTCTCGTC	qRT-PCR
LcCNGC6-Fq	GACAAGTAACATTACGAGTTCCTG	qRT-PCR
LcCNGC6-Rq	TGCTACCCACTTGTACTGATC	qRT-PCR
LcCNGC7-Fq	GATCAGTACAAGTGGTTGGAGA	qRT-PCR
LcCNGC7-Rq	GCTCACAGATAGCATCAAGC	qRT-PCR
LcCNGC8-Fq	GCTACGACGATTGGAAATTCG	qRT-PCR
LcCNGC8-Rq	AGTCCACTGAGCACTGTG	qRT-PCR
LcCNGC10-Fq	GACACTCCAGAGCAGATCATG	qRT-PCR
LcCNGC10-Rq	AGGCAAGTTAGAACCTTAGAGG	qRT-PCR
LcCNGC13-Fq	GATGTGCTTGTCATGTTCTGTG	qRT-PCR
LcCNGC13-Rq	GGAAGAACGGCAAGAATGTC	qRT-PCR
LcCNGC14-Fq	CAGTCACCGTTACCATTTCTG	qRT-PCR
LcCNGC14-Rq	CTTAGGTCTCTAGGCAGATTATGA	qRT-PCR
LcCNGC17-Fq	ACTCGGTTTGCCTCTTGCG	qRT-PCR
LcCNGC17-Rq	TGGCTGCCCTTTGTATCCC	qRT-PCR
Lc18s rRNA-Fq	GTGTTCTTCGGAATGACTGG	qRT-PCR
Lc18s rRNA-Rq	ATCGTTTACGGCATGGACTA	qRT-PCR
LcCNGC13-2055-F	gagctcggtacccgg**ggatcc**ATGAATGGAGAAGATGATGCCAT	subcellular location
LcCNGC13-2055-R	gcccttgctcaccat**ggatcc**GACGTTGCTATGTTTTGGCAAG	subcellular location
